# Climate Change, Humans, and the Extinction of the Woolly Mammoth

**DOI:** 10.1371/journal.pbio.0060079

**Published:** 2008-04-01

**Authors:** David Nogués-Bravo, Jesús Rodríguez, Joaquín Hortal, Persaram Batra, Miguel B Araújo

**Affiliations:** 1 Department of Biodiversity and Evolutionary Biology, National Museum of Natural Sciences, Consejo Superior de Investigaciones Científicas, Madrid, Spain; 2 National Research Center on Human Evolution, Centro Nacional De Investigación Sobre La Evolución Humana, Burgos, Spain; 3 Natural Environment Research Council Centre for Population Biology, Imperial College London, Ascot, Berkshire, United Kingdom; 4 Department of Earth and Environment, Mount Holyoke College, South Hadley, Massachusetts, United States of America; University of California, United States of America

## Abstract

Woolly mammoths inhabited Eurasia and North America from late Middle Pleistocene (300 ky BP [300,000 years before present]), surviving through different climatic cycles until they vanished in the Holocene (3.6 ky BP). The debate about why the Late Quaternary extinctions occurred has centred upon environmental and human-induced effects, or a combination of both. However, testing these two hypotheses—climatic and anthropogenic—has been hampered by the difficulty of generating quantitative estimates of the relationship between the contraction of the mammoth's geographical range and each of the two hypotheses. We combined climate envelope models and a population model with explicit treatment of woolly mammoth–human interactions to measure the extent to which a combination of climate changes and increased human pressures might have led to the extinction of the species in Eurasia. Climate conditions for woolly mammoths were measured across different time periods: 126 ky BP, 42 ky BP, 30 ky BP, 21 ky BP, and 6 ky BP. We show that suitable climate conditions for the mammoth reduced drastically between the Late Pleistocene and the Holocene, and 90% of its geographical range disappeared between 42 ky BP and 6 ky BP, with the remaining suitable areas in the mid-Holocene being mainly restricted to Arctic Siberia, which is where the latest records of woolly mammoths in continental Asia have been found. Results of the population models also show that the collapse of the climatic niche of the mammoth caused a significant drop in their population size, making woolly mammoths more vulnerable to the increasing hunting pressure from human populations. The coincidence of the disappearance of climatically suitable areas for woolly mammoths and the increase in anthropogenic impacts in the Holocene, the coup de grâce, likely set the place and time for the extinction of the woolly mammoth.

## Author Summary

What caused the woolly mammoth's extinction? Climate warming in the Holocene might have driven the extinction of this cold-adapted species, yet the species had survived previous warming periods, suggesting that the more-plausible cause was human expansion. Testing these competing hypotheses has been hampered by the difficulty in generating quantitative estimates of the relationship between the mammoth's contraction and the climatic and/or human-induced drivers of extinction. In this study, we combined paleo-climate simulations, climate envelope models (which describe the climate associated with the known distribution of a species—its envelope—and estimate that envelope's position under different climate change scenarios), and a population model that includes an explicit treatment of woolly mammoth–human interactions to measure the extent to which climate changes, increased human pressures, or a combination of both factors might have been responsible. Results show a dramatic decline in suitable climate conditions for the mammoth between the Late Pleistocene and the Holocene, with hospitable areas in the mid-Holocene being restricted mainly to Arctic Siberia, where the latest records of woolly mammoths in continental Asia have been found. The population model results also support the view that the collapse of the climatically suitable area caused a significant drop in mammoth population size, making the animals more vulnerable to increasing hunting pressure from expanding human populations. The coincidence of the collapse of climatically suitable areas and the increase in anthropogenic impacts in the Holocene are most likely to have been the “coup de grâce,” which set the place and time for the extinction of the woolly mammoth.

## Abstract 3

Climate models together with population models provide quantitative evidence that the combined effects of climate change and anthropogenic pressures can explain the extinction of the woolly mammoths.

## Introduction

The woolly mammoth, *Mammuthus primigenius*, was an herbivorous mammal that lived in the cool and dry open steppe-tundras of the Northern Hemisphere from late Middle Pleistocene (300 thousand years before presend [ky BP]), or even earlier [1]. They are thought to have finally become extinct 3.7 ky ago, on Wrangel Island, Arctic Siberia, [2]. The climate became progressively cooler and drier from the last interglacial period (126 ky BP), to the Last Glacial Maximum (21 ky BP), and then became warmer and wetter toward the mid-Holocene (6 ky BP). These profound climatic oscillations produced a transformation of the vegetation and a reduction in the geographical range of open steppe-tundra habitats, where the last woolly mammoths lived during the mid-Holocene [3]. At the same time, human populations started dispersing across northern Eurasia around 40 ky BP [4]. While data confirm the coexistence of woolly mammoths and humans [5], some authors suggest that direct evidence of woolly mammoth hunting is scarce [6]. Previous analyses have related the contraction of the mammoth's geographical range and other Late Quaternary Extinctions to both environmental [7–9] and anthropogenic factors [10,11], or a combination of both [12], but they have often been based upon qualitative or descriptive approaches (but see [13] and [14]). Although the pattern of contraction of their geographical range is known [3,15–17], progress concerning the contribution of environmental factors [18] to explain the extinction of woolly mammoths requires a more quantitative assessment of the contraction of their geographical range and the collapse of suitable climate conditions (Figure S1).

We combined a climate envelope model and a dynamic population model to investigate the extent to which the extinction of the woolly mammoth might have been driven by the collapse of its suitable climate conditions and the intensification of human hunting. The climate envelope of the woolly mammoth was characterised based on statistical associations between the fossil record and palaeo-climate simulations [19,20]. We compiled the ^14^C-dated distribution of fossil records of woolly mammoths in Eurasia for four time periods (*∼*42 ky BP, 30 ky BP, 21 ky BP, and 6 ky BP) and palaeo-climate simulations for 126 ky BP, 42 ky BP, 30 ky BP, 21 ky BP, and 6 ky BP periods to characterise and project the mammoth's climatic envelope (Figure S1). We assumed that the climate envelope of the mammoth can be reasonably described using three variables: mean temperature of the coldest month, mean temperature of the warmest month, and annual precipitation. We modelled this envelope combining data for three periods: 42 ky BP, 30 ky BP, and 21 ky BP, and we projected the distribution of the climatic conditions suitable for woolly mammoth for the 126 ky BP and the 6 ky BP periods. We used these results to estimate the decrease in number of woolly mammoths, and we modelled the hunting intensity needed to extinguish the species in four periods: 42 ky BP, 30 ky BP, 21 ky BP, and 6 ky BP. Our results show that the extent of suitable conditions for woolly mammoths in Eurasia progressively collapsed after 42 ky BP: 89% of the species' geographical range disappeared between 42 ky BP and 6 ky BP, probably causing a drop in population size and making the species vulnerable to hunting pressures from a growing human population.

## Results and Discussion

**Figure 1:**
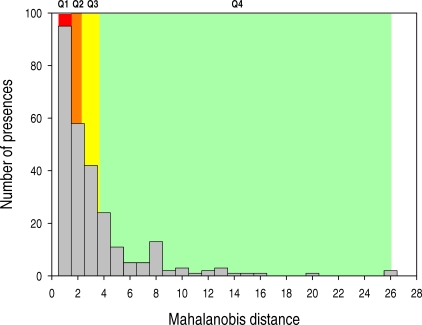
Number of Records of Mammoth Presence along the Climatic Suitability Gradient The suitable climatic conditions were split into quartiles (Q1, red; Q2, orange; Q3, yellow; Q4, green). The suitability gradient is measured using MD scores that represent the climatic difference between each of the woolly mammoth's records (radiocarbon calibrated for 42 ky BP, 30 ky BP, and 21 ky BP periods) and the average climatic conditions for the three periods combined. Increasing MDs represent decreasing suitability of the climate.

We first evaluated whether the measured climatic conditions in which the woolly mammoths were living changed during the Late Pleistocene. We found that their climatic preferences did not differ significantly during the 42 ky BP, 30 ky BP, and 21 ky BP periods (Kruskal-Wallis test, *n* = 54, *n* is the number of locations with a fossil presence of woolly mammoths, *p* = 0.186 for mean temperature of the warmest month, *p* = 0.504 for mean temperature of the coldest month, *p* = 0.536 for annual precipitation). We also found that their climatic preferences did not differ statistically when we replicated the analysis with *n* = 141. On the contrary, we found that they differ when we replicated the analysis with all the records of woolly mammoths, *n* = 270 (See Table S1. This disagreement could be the result of the effect of spatial autocorrelation in the *p*-values of the replication with all the cases, or could be because of incomplete/biased fossil records, or because of slight inaccuracies in the climate simulations). During the three periods analysed, woolly mammoths occupied areas with, on average, 240 mm precipitation per year, and temperatures ranging from −30.3 °C to 14.5 °C as the coldest and warmest months, respectively (Figure S2). We split all measured climatic suitability scores into quartiles (Figure 1) to describe different degrees of climate suitability for the mammoth. Deviation from the most suitable conditions is associated with higher Mahalanobis distance (MD) scores (see Material and Methods). Therefore, the most suitable conditions are represented by the first quartile of suitability scores (Q1), corresponding to MD scores below 1.02, and the less suitable conditions within the modelled niche (Q4) correspond to suitability scores above 3.27.

**Figure 2:**
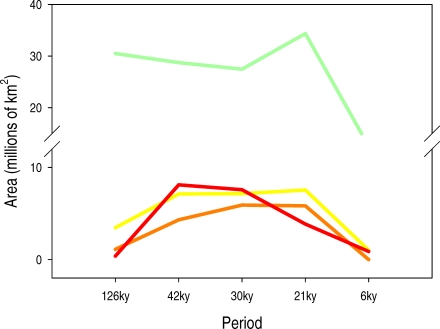
Change in the Area (%) of the Different Suitable Climatic Conditions for Woolly Mammoths The suitability of climatic conditions based on MD was split into quartiles (Q1, red; Q2, orange; Q3, yellow; Q4, green). Q1 represents the most suitable conditions and Q4 represents the less suitable. The extent of the most suitable conditions for woolly mammoths was smaller during warmer interglacial periods, 126 ky BP and 6 ky BP.

Our results show that the most suitable geographic area available to woolly mammoths (Figure 2), Q1, increased by 7.7 million km^2^ from the last interglacial, 126 ky BP, to 42 ky BP (from 0.3 to 8.1 million km^2^). There was a 0.5 million km^2^ decrease in the most suitable area between 42 ky BP and 30 ky BP periods, and then a 3.7 million km^2^ decrease between 30 ky BP and 21 ky BP (from 7.5 to 3.8 million km^2^). Finally, between 21 ky BP and 6 ky BP, there was a 2.9 million km^2^ decrease. By the 6 ky BP period, only 0.8 million km^2^ of the most suitable climatic conditions, Q1, remained (Figure 2 and Figure S3).

**Figure 3:**
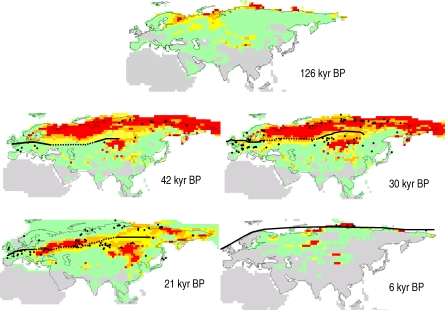
Maps of Projected Climatic Suitability for the Woolly Mammoths in the Late Pleistocene and Holocene Suitability scores are divided into four colour-scale classes (quartiles 1 [more suitable] to 4 [less suitable] of the MD), where increasing intensities of red represent increasing suitability of the climate and increasing intensities of green represent decreasing suitability. Black points are the records of mammoth presence for each of the periods. Black lines represent the northern limit of modern humans [59]. Black dotted lines indicate uncertainty in the limit of modern humans.

**Figure 4:**
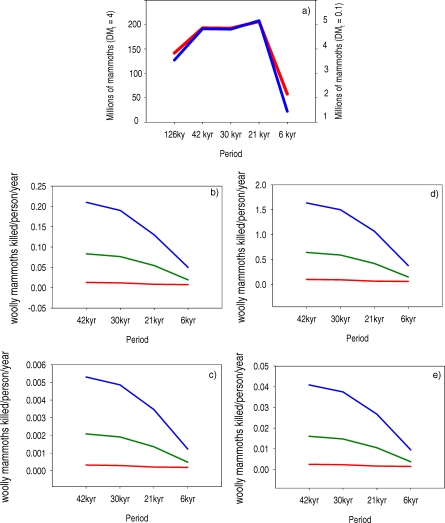
Estimated Number of Woolly Mammoths in Eurasia in Five Time Periods (A) and the Number of Woolly Mammoths Required To Be Killed per Person per Year (HI_t_) to Drive the Woolly Mammoth Population to Extinction in Their Area of Co-Existence for Four Time Periods (B–E) (A) The red line, left *y*-axis, assumes a woolly mammoth population density (*Dm_t_*) of 4 individuals/km^2^ and the blue line of 0.1 individual/km^2^, right *y*-axis. Population density is considered to be time-independent. (B) The plot considers a suboptimal woolly mammoth population (CR = 0.35) with a woolly mammoth density of 4 individuals/km^2^. (C) This plot considers suboptimal woolly mammoth population (CR = 0.35) with a woolly mammoth density of 0.1 individuals/km^2^. (D) This plot considers a vigorous woolly mammoth population (CR = 2.7) with a woolly mammoth density of 4 individuals/km^2^. (E) This plot considers vigorous woolly mammoth population (CR = 2.7) with a woolly mammoth density of 0.1 individuals/km^2^. Colours represent three different estimations of AMH population density [56]. Green lines: average value, red lines: maximum value, blue lines: minimum value.

A large reduction in the available suitable climate conditions for the species is expected to cause a reduction in its distributional range, thus contributing (Figure 3) to a reduction in woolly mammoth population sizes and therefore a potential increase in the extinction risk [21]. This hypothesis is supported, firstly, by our estimation of reduced range area and hence reduced population sizes of woolly mammoths through time (see Materials and Methods). A marked reduction in population size of the woolly mammoths is evidenced for the Holocene (6 ky BP), whatever the woolly mammoth population density value selected (Figure 4A). Secondly, the assumption is supported by the results of a model of hunting intensity (HI; the number of woolly mammoths required to be killed per person per year in order to drive the species to extinction; see Materials and Methods). Irrespective of the cull rate used (CR; the percentage of the mammoth population that must be killed to drive the species to extinction), HI clearly varies through time; the number of woolly mammoths that need to be killed per person per year in order to drive the species into extinction is fairly similar for the 42 ky BP and the 30 ky BP periods, starts to decrease by the 21 ky BP period, and becomes very low in the 6 ky BP period (Figure 4B–4E and Table S2). According to our analyses, even a high density (4 individuals/km^2^) and vigorous (CR = 2.7%; see Materials and Methods) woolly mammoth population would have been driven to extinction with an HI of 0.37 individuals killed per person per year in the 6 ky period. In other words, for these optimistic parameters, one woolly mammoth killed every three years by each human being inhabiting its distribution range would be sufficient to lead the species to extinction. With a low density (0.1 individuals/km^2^) and suboptimal woolly mammoth population (CR = 0.35%; see Materials and Methods), the HI value drops down to 0.0049 woolly mammoths killed per person per year; this is roughly one mammoth killed by each person every 200 years. These results support the view that the synergy between the collapse of suitable climatic conditions for the woolly mammoths and northward increase in human population densities during the Holocene set the place and time of the woolly mammoth's extinction.

The last nonislands records of the woolly mammoth in the Holocene [22] (dated after 11 ky BP) were found around the Tamyr Peninsula, Bikada and Nizhnaya Taimyra rivers, and the Pronchishchev Coast (Figure 3), coinciding with areas classified by our models as highly suitable for woolly mammoths at 6 ky BP (scores of MD below 0.52; Figure S4). The youngest remains of woolly mammoth found on Wrangel Island are located within the less suitable (Q4) regions (MD score of 6.03). The quality of our projections is further supported by the high spatial agreement between our climate suitability model for the woolly mammoth and the line delimiting the forest [23] and open tundra habitats in the 6 ky BP period (Figure S4 and Protocol S1). This correspondence provides an independent evaluation [24] of the accuracy with which our climate envelope models infer the environmental conditions that would have affected the survival of woolly mammoths in Eurasia.

Theories about species extinctions rely on two different paradigms [25], which consider either the factors contributing to the general decline of species before their populations become rare—the declining-species paradigm [26,27]—or the genetic and demographic factors promoting the extinction of small populations—the small-population paradigm [28]. Most debate about the extinction of the woolly mammoth has focused on trying to separate the contributions of humans [29,30] and environmental changes [7–9] toward the extinction of the species. Our results support both perspectives. We suggest that the final extinction of the mammoth might have been the result of the combined effects of climate change and human impacts involving both extinction paradigms within the common framework of metapopulation dynamics [31]. By quantifying the magnitude of the impacts of climate change on woolly mammoth distributions for different periods of time, we show that climate change posed serious challenges for the survival of the species and those areas with suitable climate conditions for the woolly mammoth became severely reduced at 6 ky BP. In the absence of human hunting, however, mammoth populations might have been able to survive in small pockets of suitable habitat and use suboptimal habitats outside the core of their climate envelope, as must have happened at 126 ky BP (Figure 3). Our analyses suggest that the humans applied the coup de grâce and that size of the suitable climatic area available in the mid-Holocene was too small to host populations able to withstand increased human hunting pressure.

Our envelope model for the 6 ky BP period also projected the existence of highly suitable conditions for the occurrence of woolly mammoths outside the High Arctic Siberia (Figure S4) in places where no records have been found, such as in the Ob River basin (60 °N – 75 °E) or southward just within Mongolia (49 °N – 95 °E). The predicted suitable conditions for woolly mammoths in Mongolia, for example, coincide with the Uvs Nuur Basin, a UNESCO World Heritage Centre, that currently represents one of the best-preserved natural steppe landscapes of Eurasia. If these areas had the potential to host core populations, understanding what happened there would enlighten our knowledge about the last days of the mammoth. To contribute to this debate, new surveys in these areas should be undertaken to (a) determine whether populations remained there during mid-Holocene and (b) examine why woolly mammoths disappeared and were excluded from these regions. Our results suggest that climate change and human impacts progressively cornered the mammoth in the northernmost land masses of Arctic Siberia and some arctic islands, leaving them with nowhere to run away from extinction.

## Materials and Methods

### Mammoth data.

Records of presence for woolly mammoths were obtained from printed sources and public online databases (Dataset S1). Two different types of radiometrically dated occurrences were accepted: directly dated mammoth fossils and dates obtained from other materials in the mammoth bearing layer. In direct datings, it is assumed that different ages for the same locality represent different individuals that died at different times (unless evidence exists to the contrary). In indirect datings, when several dates were available for a single layer, we computed an age interval for the layer, taking the upper and lower confidence limits of the oldest and youngest dates, respectively, and eliminating occurrences with incoherent or widely varying age estimates. Radiocarbon dates (uncalibrate ^14^C dates) were calibrated into calendar years (including 95% confidence intervals) using the CalPal 2005 SFCP calibration curve [32]. We assumed a 6 ky time interval (i.e., 3 ky above and below interval date) as an arbitrary temporal window. Woolly mammoth occurrences for each time interval were defined as those having their calibrated 95% confidence intervals within these time intervals (42 ± 3 ky BP, 30 ± 3 ky BP, 21 ± 3 ky BP, and 6 ± 3 ky BP, respectively), resulting in 270 records being included. We estimated the climatic conditions for the locations with woolly mammoth records from the global climate models (GCMs) outputs.

### Palaeoclimatic simulations.

We estimated the climatic conditions for the locations with woolly mammoth records from GCM simulations. Palaeoclimatic simulations were performed with the GENESIS 2 GCM [33]. Five simulations were used: one for the Eemian (∼126 ky BP), two for Oxygen Isotope Stage 3 (OIS 3), one for the Last Glacial Maximum (LGM; ∼21 ky BP), and one for the mid-Holocene (∼6 ky BP). The OIS 3 simulations represent the warmer middle part (∼42 ky BP), and colder later part (∼30 ky BP) of Stage 3. Modelled annual averaged temperatures for the OIS 3 warm simulation are approximately 1–2 °C warmer than the OIS 3 cold simulation over Europe and 0.5–1 °C warmer over most of Asia. Carbon dioxide levels were specified at 345 ppm for the Eemian simulation [34], 200 ppm for the OIS 3 and LGM simulations [35], and 280 ppm for the mid-Holocene simulation [36]. Sea surface temperatures (SSTs) for the OIS 3 and Last Glacial Maximum simulations were taken primarily from CLIMAP [37], with modifications from GLAMAP-2000 and other sources [38]. SSTs for the mid-Holocene simulation were prescribed at present-day values [39]. Ice sheets for the OIS 3 and LGM simulations followed the ICE-4G [40] and other reconstructions [38, 41], while present-day ice sheets were used for the mid-Holocene simulation [36,38]. The Eemian interglacial simulation uses a mixed-layer slab ocean with dynamic sea ice [34]. Simulated Eemian temperatures in Eurasia are warmer than present-day and show reasonable agreement with temperatures inferred from pollen and plant macrofossils [42]. In all cases, insolation was calculated using orbital parameters [43,44]. The Eemian simulation used prescribed vegetation, the OIS 3 and LGM simulations were interactively coupled to the BIOME4 vegetation model [45], and the mid-Holocene simulation was interactively coupled to the EVE vegetation model [46]. All simulations were spun up to equilibrium. Results are 10-y averages. Temperatures are in °C and precipitation is in mm per year.

### Testing for differences between the climate conditions occupied by the species at 42 ky BP, 30 ky BP, and 21 ky BP periods.

We used a Kruskal-Wallis test, a nonparametric alternative to one-way ANOVA, to test for differences between the climate conditions occupied by the species at 42 ky BP, 30 ky BP, and 21 ky BP periods; *p* > 0.05 was taken to indicate that climate conditions do not differ significantly between time periods. To avoid inflation of *p*-values due to spatial autocorrelation in the Kruskal-Wallis test, we randomly filtered out 80% of the cases (216 of 270 cases were removed). This filtering process was only applied to the Kruskal-Wallis test. To define the climatic niche of the mammoth, we used all of the available 270 records. We used MD to model the ecological niche of the woolly mammoths. We repeated the same analysis filtering out 129 of 270 cases.

### Mahalanobis distance technique.

The MD technique relies on a multivariate mean and a covariance matrix, and performs an oblique positioning of an elliptical envelope within a multidimensional climatic space. Such an envelope is defined by combinations of climatic variables with equal MD to a vector of average climatic conditions, defined as the mean of all the observations available for the target species. MD scores should be interpreted as a similarity index to sites where the species has been recorded. Mathematically, the MD is defined as:

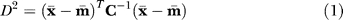
where **m** is the mean vector and **C** is the covariance matrix of **S**. The rows (vectors) of **S** stand for observations of fossil presence of woolly mammoths and the columns for climatic indices. **S**, therefore, represents the climatic conditions from grid cells with a fossil presence of woolly mammoths. The *T* superscript denotes the transpose operator. The vector **m** represents the average climatic conditions from grid cells with a fossil presence of woolly mammoths, and **x** is a vector indicating climatic conditions of a particular grid cell with a fossil presence of woolly mammoth. We performed a bootstrap with *n* = 1,000 resampling runs to assess the stability of model projections using the boot library in R. Through resampling with replacement of the rows of observations, the bootstrap allows us to estimate **m** and **C**, and then subtract the estimate of accuracy from the initial real measure to obtain a corrected estimate. Bootstrapping shows that corrected and estimated values of **m** and **C** were similar (Figure S5 and Figure S6). Finally, we divided MD scores into quartiles (Q1, Q2, Q3 and Q4, with increasing MD scores, i.e., decreasing climatic suitability), and mapped the potential range of woolly mammoths for each quartile during each period, projecting it also to the 126 ky BP and 6 ky BP intervals. Previous studies [47,48], on 192 plant species in Israel and 71 plant species in southern Africa, using the MD approach, only qualified as potentially suitable those areas with MD scores below 4 and 2.5, respectively.

### HI model.

The aim of the HI model is to estimate the hunting intensity by anatomically modern humans (AMH) necessary to drive the whole woolly mammoth population of Eurasia to extinction. HI is thus defined as the number of woolly mammoths killed per person per year. Let HI*_t_* be the hunting intensity necessary to drive woolly mammoths to extinction at time interval *t*:

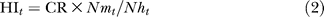
where CR is the cull rate, defined as the percentage of the woolly mammoth population that must be killed to drive it to extinction. CR was based on the computer-based simulation of mammoth population dynamics and exploitation in constant, fluctuating and deteriorating environments developed by Mithen [14]. According to Mithen's simulations, a cull greater than 2.7% of the total number of individuals may drive to extinction a vigorous mammoth population in a constant environment, and a proportion as low as 0.35% will do for a less vigorous, suboptimal, population. Thus, we computed our model using both values for CR. It is important to note that in Mithen's model, this CR represents the killing of animals which would have otherwise survived until the following year. Thus, it does not include the death of old or weak animals which would have died from other causes. *Nm_t_* and *Nh_t_* are the total population size (number of individuals) of, respectively, woolly mammoths and humans at each time interval *t* (42 ky BP, 30 ky BP, 21 ky BP, and 6 ky BP periods), and are obtained from:

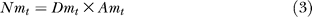


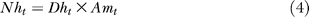
where *Dm_t_* is mammoth population density (individuals/km^2^), *Dh_t_* is human population density (individuals/km^2^), and *Am_t_* is the area of coexistence of woolly mammoths and AMH for time interval *t*, since AMH should coexist with mammoths to hunt them (therefore, *Am_t_* is removed from the equation because it represents the same area for woolly mammoths and humans). Thus, equation (2) may be written as:

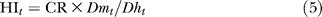
and HI*_t_* represents the hunting intensity necessary to drive the woolly mammoth population to extinction inside their area of coexistence with AMH for time interval *t*.

A range of woolly mammoth population densities (*Dm*) were estimated based on population densities of modern elephants and allometric body mass relationships. African elephants (Loxodonta africana) are considered a good analogue for woolly mammoths [49], and their reported average population density is 1.09 individuals/km^2^ [50], although it varies within the range of 0.25 to 5 individuals/km^2^ [51]. Population density for Asian elephants, Elephas maximus, the other extant proboscidean species, ranges from 0.12 to 1.0 individuals/km^2^ [51,52]. Since both are tropical species and population density is known to depend on primary productivity, which decreases with latitude [53,54], these values likely overestimate actual mammoth densities. Thus, we estimated *Dm* from woolly mammoth body mass using the allometric equation computed by [55] for temperate herbivorous mammals, obtaining maximum and minimum values of 0.74 and 1.79 individuals/km^2^ respectively. However, [14] estimated Siberian woolly mammoth population densities in the range 0.038 to 0.23 individuals/km^2^. Due to this high variation of estimates, we decided to use 0.1 and 4 individuals/km^2^ as a conservative and an optimistic estimates of the possible range of mammoth population densities. For simplicity, these estimates of *Dm* were considered to be time-independent and homogeneous across the entire area that is environmentally suitable for woolly mammoths.

AMH population densities (*Dh_t_*) for the four time intervals considered in our analysis were obtained from [56]. We used three different estimates of *Dh_t_* (Table S3). Their minimum *Dh_t_* for each cultural period, for example—assuming that the Aurignacian estimate represents our 42 ky BP period, the Gravettian estimate represents our 30 ky BP interval, the Glacial maximum represents our 21 ky BP period, and the Late Glacial represents our 6 ky BP period—were 0.066 individuals/km^2^, 0.072 individuals/km^2^, 0.101 individuals/km^2^, and 0.285 individuals/km^2^, respectively (see Table S3 for average and maximum population densities). The likely effect of the temporal discrepancies between our data and the periods defined in [56] would be the underestimation of human population density for the 6 ky BP period and the slight overestimation for 42 ky BP and 30 ky BP. Also, we consider human population density to be homogeneous across all the area that was environmentally suitable for the woolly mammoth, which is an optimistic estimate of the abilities of ancient human populations to survive at high latitudes during the upper Pleistocene. Indeed, the first recorded human presence above 60 °N dated from 11 ky BP [57]. As a result, our model will tend to underestimate the HI*_t_* value for 42 ky BP, 30 ky BP, and 21 ky BP, and to overestimate its value for 6 ky BP.

### Woolly mammoth population reduction through time.

Total Eurasian woolly mammoth population sizes for the five time intervals (126 ky BP, 42 ky BP, 30 ky BP, 21 ky BP, and 6 ky BP) have been estimated assuming (a) that the entire environmentally suitable area is occupied (Q1 + Q2 + Q3 + Q4); and (b) that woolly mammoth population density *Dm_t_* is homogeneous throughout the area and is comprised between 0.1 and 4 individuals/km^2^. Since both assumptions are simplistic, the obtained numbers might overestimate the actual metapopulation size, although they are useful to represent the general trend through time. A marked reduction in population size is seen for the Holocene (6 ky BP), whatever the woolly mammoth population density value selected.

## Supporting Information

Dataset S1 Dated Woolly Mammoth Occurrences in Eurasia Max Ca BP l and Min Cal BP represent the 95% confidence interval for the age for radiocarbon dates. Radiocarbon ages have been converted using CalPal 2005 SFCP. Woolly mammoth occurrences for each time interval were defined as those having their calibrated 95% confidence intervals within these time intervals (42 ± 3 ky BP, 30 ± 3 ky BP, 21 ± 3 ky BP, and 6 ± ky BP, respectively), resulting in 270 records being included for modelling the climatic niche of the woolly mammoths. Data summary references are listed below.
(2.5 MB DOC)

Figure S1 A Visual Example of the Climatic Niche Concept, Its Geographical Location, and Niche Conservatism The climatic niche represents the climatic conditions where a species is able to persist. It would encompass many climate variables (*n* dimensions). The climatic niche has a location in geographical space. On the left, we show an example of a climatic niche (yellow ellipse) defined by the distribution (black outlined symbols equal presence) of a species in three different periods of time, *t*_1_ (orange squares), *t*_2_ (green circles), and *t*_3_ (blue triangles), within a climatic space defined by two dimensions (two climatic gradients represented by precipitation and temperature). The mean climate conditions are symbolized by the black cross. On the right, we observe the geographical location of the climatic niche in the three periods of time and its projection to a fourth one, *t*_4_. Note that while the climatic conditions where the species is present remains constant (i.e., the records of the species in the three periods can be placed anywhere within the ellipse of the environmental scatterplot), climate change modifies the geographical location and the extent of the areas with conditions suitable for the species.
(76 KB DOC)

Figure S2 The Climatic Niche of Woolly Mammoths Records (*n* = 270) of woolly mammoth presence during OIS 3 Warm Phase, 42 ky BP (orange dots), OIS 3 Cold Phase, 30 ky BP (green dots) and LGM, 21 ky BP (blue dots). The niche is plotted in a three-dimensional space and also in three plots of two-dimensions to enable an easier interpretation of the results. Mean temperature of the coldest month, *T*_cm_; mean temperature of the warmest month, *T*_wm_; annual precipitation, *R*_ann_.
(196 KB DOC)

Figure S3 Number of Grid Cells (2° Resolution) in Relation to MD Scores Red bars: 6 ky BP period; blue bars: 21 ky BP period; green bars: 30 ky BP period; orange bars: 42 ky BP period. Deviation from the mean conditions is associated with higher MD scores. The reduction in the area of mean climatic conditions for woolly mammoths in Eurasia shows a clear trend, irrespective of the different ways (e.g., quartiles) in which the niche could be described.
(62 KB DOC)

Figure S4 The Projected Climatic Niche of Woolly Mammoths and the Vegetation for the 6 ky BP Period The map shows in more detail the climatic niche of woolly mammoths projected by our model for the 6 ky BP period. The most suitable climatic conditions are plotted in red (Q1). Q3 is plotted in yellow and Q4 in green. The dark green line represents the limit of the distribution of the birch forest, Betula spp., for the 6 ky BP period as published by MacDonald et al. [23] based on radiocarbon-dated macrofossils. Treelines for larch (Larix spp.) and spruce (Picea obovata) were located at similar latitude [23]. Small divergences between the red areas and the green line are the result of the coarse resolution of the maps generated by GENESIS 2 (2° × 2°). The use of bioclimatic models to assess the extent to which niches may or may not be reduced for many species, has been suggested [18] as one of the future steps of research on the Late Quaternary extinctions debate. Martínez-Meyer et al. [58] is an example of the use of bioclimatic models to assess past changes in the potential geographical range of species.
(164 KB DOC)

Figure S5 Bootstrap Plots of the Covariance Matrix **C**
MD technique relies on a mean vector and a covariance matrix. Blue bars are the real covariance values and the black lines are the covariance values simulated by the bootstrapping procedure. *T*_cm_, mean temperature of the coldest month; *T*_wm_, mean temperature of the warmest month; and *P,* annual precipitation.
(109 KB DOC)

Figure S6 Bootstrap Plots of the Distances to the Most Suitable Climatic Conditions: Mean Vector **m**
The MD technique relies on a mean vector and a covariance matrix. Blue bars are the observed mean vector values and the black lines are the mean vector values simulated by bootstrapping. *T*_cm_, mean temperature of the coldest month; T_wm_, mean temperature of the warmest month; and *P,* annual precipitation.
(37 KB DOC)

Protocol S1 BIOCLIM and Maxent Models of Woolly Mammoth's Niche. (313 KB DOC)

Table S1 Kruskal-Wallis Test Statistics (see Material and Methods) Table S1A filtered out 216 cases of 270; Table S1B filtered out 129 cases of 270. Table S1C did not filter out any case (*n* = 270). Significance levels above 0.05 indicate that the climate conditions do not differ between 42 ky BP, 30 ky BP, and 21 ky BP periods.
(24 KB DOC)

Table S2 HI_t_ Values through Time Number of woolly mammoths (NK) that should be killed per year in each time interval and hunting intensity (HI_t_) measured as woolly mammoths killed by human individual per year necessary to drive the entire population to extinction. CR, cull rate (individuals/y); *Dm*, woolly mammoth population density (individuals/km^2^). Values are calculated assuming the minimum human population densities for each period (see Table S3 for densities of human populations)
(58 KB DOC)

Table S3 Demographic Density of AMH in Europe up to 40 °E in Four Different Cultural Periods [56] (29 KB DOC)

## Abbreviations

AMH: anatomically modern human
CR: cull rate
*Dh*: human population density (individuals/km^2^)
*Dm*: woolly mammoth population density (individuals/km^2^)
GCM: general circulation model
HI: hunting intensity (mammoths killed per person per year)
MD: Mahalanobis distance
Q1: first quartile
Q2: second quartile
Q3: third quartile
Q4: fourth quartile
ky BP: thousand years before present
