# Supplementary material for: Climate Change, Humans, and the Extinction of the Woolly Mammoth
Source: PLoS Biol. 2008 Apr 1;6(4):e79. doi: 10.1371/journal.pbio.0060079 (PMC2276529; doi:10.1371/journal.pbio.0060079)
Supplement: Supplementary file 1 [file pbio.0060079.sd001.doc]

| Codelay | Locality | Layer | Longitude | Latitude | Sigma | Max Cal BP | Min Cal BP | Technique | References |
| --- | --- | --- | --- | --- | --- | --- | --- | --- | --- |
| 3 | Labeko Koba | IV | -2.5 | 43.07 | 305 | 27450 | 24850 | 14C/AMS 14C | 1; 20 |
| 890 | Robin Hood's Cave | N/A | -1.19 | 53.27 | 120 | 14920 | 13880 | 14C/AMS 14C | 9 |
| 856 | Pin Hole Cave | N/A | -1.1957 | 53.264 | 2100 | 50330 | 42650 | 14C/AMS 14C | *6* |
| 716 | Lespezie-Lutarie | N//A | 26.75 | 46.67 | 320 | 22680 | 20480 | 14C/AMS 14C | 2 |
| 437 | Bora gran d'en carreres | N/A | 2.87 | 41.87 | 500 | 14810 | 12370 | 14C/AMS 14C | 4; 17 |
| 362 | Achchagy-Allaikha River | N/A | 147.3 | 69 | 60 | 15100 | 14540 | 14C/AMS 14C | 15 |
| 362 | Achchagy-Allaikha River | N/A | 147.3 | 69 | 80 | 15210 | 14530 | 14C/AMS 14C | 15 |
| 362 | Achchagy-Allaikha River | N/A | 147.3 | 69 | 1000 | 52490 | 46130 | 14C/AMS 14C | 15 |
| 363 | Achinsk | N/A | 90.42 | 56.25 | 340 | 30510 | 28710 | 14C/AMS 14C | 15 |
| 365 | Afontova Gora 2 | 2 | 92.5 | 56.1 | 250 | 17470 | 15110 | 14C/AMS 14C | 15 |
| 366 | Afontova Gora 2 | 3 | 92.5 | 56.1 | 60 | 17130 | 16610 | 14C/AMS 14C | 15 |
| 367 | Afontova Gora 2 | 4 | 92.5 | 56.1 | 70 | 17260 | 17060 | 14C/AMS 14C | 15 |
| 368 | Afontova Gora 2 | N/A | 92.5 | 56.1 | 200 | 17480 | 16360 | 14C/AMS 14C | 15 |
| 368 | Afontova Gora 2 | N/A | 92.5 | 56.1 | 80 | 17410 | 17210 | 14C/AMS 14C | 15 |
| 369 | Afontova Gora 3 | N/A | 92.5 | 56.1 | 130 | 17760 | 17240 | 14C/AMS 14C | 15 |
| 371 | Aion Island | N/A | 168 | 69.78 | 130 | 16700 | 14940 | 14C/AMS 14C | 15 |
| 371 | Aion Island | N/A | 168 | 69.78 | 120 | 17480 | 17200 | 14C/AMS 14C | 15 |
| 371 | Aion Island | N/A | 168 | 69.78 | 170 | 17740 | 17180 | 14C/AMS 14C | 15 |
| 380 | Alei River | N/A | 83 | 52.58 | 1400 | 55640 | 47520 | 14C/AMS 14C | 15 |
| 382 | Amydai River | N/A | 119 | 70 | 100 | 22590 | 22230 | 14C/AMS 14C | 15 |
| 382 | Amydai River | N/A | 119 | 70 | 400 | 48100 | 44700 | 14C/AMS 14C | 15 |
| 382 | Amydai River | N/A | 119 | 70 | 1200 | 51740 | 45500 | 14C/AMS 14C | 15 |
| 384 | Anabar Gulf | N/A | 114 | 73.54 | 500 | 44980 | 42860 | 14C/AMS 14C | 15 |
| 385 | Anabarka River | N/A | 111 | 72.1 | 300 | 35150 | 32910 | 14C/AMS 14C | 15 |
| 385 | Anabarka River | N/A | 111 | 72.1 | 500 | 42580 | 41460 | 14C/AMS 14C | 15 |
| 385 | Anabarka River | N/A | 111 | 72.1 | 400 | 45120 | 42960 | 14C/AMS 14C | 15 |
| 385 | Anabarka River | N/A | 111 | 72.1 | 800 | 47030 | 43790 | 14C/AMS 14C | 15 |
| 387 | Anabaro-Olenek interfluve | N/A | 117 | 73.6 | 500 | 42700 | 39900 | 14C/AMS 14C | 15 |
| 394 | Arylakh Lake | N/A | 107.58 | 74.42 | 40 | 13980 | 13700 | 14C/AMS 14C | 15 |
| 394 | Arylakh Lake | N/A | 107.58 | 74.42 | 290 | 39460 | 36700 | 14C/AMS 14C | 15 |
| 394 | Arylakh Lake | N/A | 107.58 | 74.42 | 450 | 42740 | 41620 | 14C/AMS 14C | 15 |
| 394 | Arylakh Lake | N/A | 107.58 | 74.42 | 580 | 44520 | 42240 | 14C/AMS 14C | 15 |
| 394 | Arylakh Lake | N/A | 107.58 | 74.42 | 970 | 46150 | 42750 | 14C/AMS 14C | 15 |
| 394 | Arylakh Lake | N/A | 107.58 | 74.42 | 1280 | 49430 | 43870 | 14C/AMS 14C | 15 |
| 394 | Arylakh Lake | N/A | 107.58 | 74.42 | 1000 | 49350 | 44350 | 14C/AMS 14C | 15 |
| 397 | Baikura-Neru Bay, Lake Taimyr | N//A | 93.1 | 74.05 | 500 | 44580 | 42420 | 14C/AMS 14C | 15 |
| 397 | Baikura-Neru Bay, Lake Taimyr | N//A | 93.1 | 74.05 | 1000 | 48790 | 44070 | 14C/AMS 14C | 15 |
| 397 | Baikura-Neru Bay, Lake Taimyr | N//A | 93.1 | 74.05 | 1000 | 49350 | 44350 | 14C/AMS 14C | 15 |
| 397 | Baikura-Neru Bay, Lake Taimyr | N//A | 93.1 | 74.05 | 1200 | 52950 | 45830 | 14C/AMS 14C | 15 |
| 399 | Baikura-Turku Bay, Lake Taimyr | N//A | 100 | 74.15 | 220 | 33260 | 31060 | 14C/AMS 14C | 15 |
| 399 | Baikura-Turku Bay, Lake Taimyr | N//A | 100 | 74.15 | 330 | 37280 | 35840 | 14C/AMS 14C | 15 |
| 407 | Baskura Peninsula | N/A | 101.5 | 74.25 | 150 | 14590 | 13550 | 14C/AMS 14C | 15 |
| 407 | Baskura Peninsula | N/A | 101.5 | 74.25 | 80 | 14460 | 13740 | 14C/AMS 14C | 15 |
| 407 | Baskura Peninsula | N/A | 101.5 | 74.25 | 190 | 25820 | 24260 | 14C/AMS 14C | 15 |
| 407 | Baskura Peninsula | N/A | 101.5 | 74.25 | 150 | 28010 | 27170 | 14C/AMS 14C | 15 |
| 407 | Baskura Peninsula | N/A | 101.5 | 74.25 | 500 | 30830 | 28710 | 14C/AMS 14C | 15 |
| 407 | Baskura Peninsula | N/A | 101.5 | 74.25 | 170 | 31200 | 30520 | 14C/AMS 14C | 15 |
| 408 | Batpak | N/A | 72.75 | 50.5 | 400 | 16960 | 13400 | 14C/AMS 14C | 15 |
| 408 | Batpak | N/A | 72.75 | 50.5 | 305 | 30460 | 28820 | 14C/AMS 14C | 15 |
| 409 | Bederbo-Tarida River | N/A | 102.16 | 73.06 | 300 | 29110 | 27590 | 14C/AMS 14C | 15 |
| 409 | Bederbo-Tarida River | N/A | 102.16 | 73.06 | 400 | 43100 | 41820 | 14C/AMS 14C | 15 |
| 409 | Bederbo-Tarida River | N/A | 102.16 | 73.06 | 500 | 44100 | 42060 | 14C/AMS 14C | 15 |
| 409 | Bederbo-Tarida River | N/A | 102.16 | 73.06 | 600 | 44200 | 42000 | 14C/AMS 14C | 15 |
| 409 | Bederbo-Tarida River | N/A | 102.16 | 73.06 | 600 | 44870 | 42630 | 14C/AMS 14C | 15 |
| 409 | Bederbo-Tarida River | N/A | 102.16 | 73.06 | 1000 | 46680 | 43000 | 14C/AMS 14C | 15 |
| 410 | Belkovsky Island | N/A | 135.83 | 75.63 | 700 | 42920 | 39080 | 14C/AMS 14C | 15 |
| 412 | Bely Yar | N/A | 88.73 | 57.48 | 320 | 34820 | 31620 | 14C/AMS 14C | 15 |
| 413 | Bennet Island | N/A | 149 | 76.7 | 60 | 15210 | 14570 | 14C/AMS 14C | 15 |
| 415 | Berelekh River | N/A | 149.05 | 70.55 | 70 | 12660 | 11940 | 14C/AMS 14C | 15 |
| 415 | Berelekh River | N/A | 149.05 | 70.55 | 130 | 14400 | 13560 | 14C/AMS 14C | 15 |
| 415 | Berelekh River | N/A | 149.05 | 70.55 | 160 | 14850 | 13770 | 14C/AMS 14C | 15 |
| 415 | Berelekh River | N/A | 149.05 | 70.55 | 110 | 16430 | 14790 | 14C/AMS 14C | 15 |
| 415 | Berelekh River | N/A | 149.05 | 70.55 | 150 | 17260 | 15300 | 14C/AMS 14C | 15 |
| 415 | Berelekh River | N/A | 149.05 | 70.55 | 800 | 18800 | 14120 | 14C/AMS 14C | 15 |
| 416 | Berezovka River | N/A | 157.3 | 67.15 | 2500 | 42800 | 31160 | 14C/AMS 14C | 15 |
| 416 | Berezovka River | N/A | 157.3 | 67.15 | 3500 | 54880 | 41480 | 14C/AMS 14C | 15 |
| 417 | Berezovy Mys | N/A | 62.38 | 59.3 | 270 | 17690 | 15730 | 14C/AMS 14C | 15 |
| 417 | Berezovy Mys | N/A | 62.38 | 59.3 | 230 | 22730 | 21690 | 14C/AMS 14C | 15 |
| 417 | Berezovy Mys | N/A | 62.38 | 59.3 | 270 | 26340 | 24780 | 14C/AMS 14C | 15 |
| 419 | Bikada River | N/A | 106.58 | 74.92 | 60 | 11630 | 11150 | 14C/AMS 14C | 15 |
| 419 | Bikada River | N/A | 106.58 | 74.92 | 100 | 14380 | 13660 | 14C/AMS 14C | 15 |
| 419 | Bikada River | N/A | 106.3 | 74.53 | 80 | 15550 | 14990 | 14C/AMS 14C | 15 |
| 421 | Biya River | N/A | 85.67 | 52.63 | 650 | 30720 | 27880 | 14C/AMS 14C | 15 |
| 423 | Bolshaya Balachnya River | N/A | 75.30 | 105.00 | 240 | 17550 | 15230 | 14C/AMS 14C | 15 |
| 423 | Bolshaya Balachnya River | N/A | 75.30 | 105.00 | 100 | 19890 | 19920 | 14C/AMS 14C | 15 |
| 423 | Bolshaya Balachnya River | N/A | 73.50 | 105.00 | 700 | 44220 | 41900 | 14C/AMS 14C | 15 |
| 423 | Bolshaya Balachnya River | N/A | 73.30 | 105.00 | 1000 | 44840 | 42000 | 14C/AMS 14C | 15 |
| 423 | Bolshaya Balachnya River | N/A | 73.60 | 100.55 | 600 | 44640 | 42360 | 14C/AMS 14C | 15 |
| 423 | Bolshaya Balachnya River | N/A | 73.53 | 100.48 | 600 | 45120 | 12840 | 14C/AMS 14C | 15 |
| 424 | Bolshaya Balachnya River, Jarkov Woolly mammoth | N/A | 105.82 | 73.53 | 130 | 24190 | 23470 | 14C/AMS 14C | 15 |
| 424 | Bolshaya Balachnya River, Jarkov Woolly mammoth | N/A | 105.82 | 73.53 | 140 | 24950 | 23910 | 14C/AMS 14C | 15 |
| 424 | Bolshaya Balachnya River, Jarkov Woolly mammoth | N/A | 105.82 | 73.53 | 160 | 24980 | 23900 | 14C/AMS 14C | 15 |
| 425 | Bolshaya Balakhnya River | N/A | 100.48 | 73.6 | 60 | 22240 | 21560 | 14C/AMS 14C | 15 |
| 425 | Bolshaya Balakhnya River | N/A | 100.48 | 73.6 | 150 | 31210 | 30650 | 14C/AMS 14C | 15 |
| 425 | Bolshaya Balakhnya River | N/A | 100.48 | 73.6 | 200 | 36600 | 35400 | 14C/AMS 14C | 15 |
| 425 | Bolshaya Balakhnya River | N/A | 100.48 | 73.6 | 240 | 37110 | 35910 | 14C/AMS 14C | 15 |
| 425 | Bolshaya Balakhnya River | N/A | 100.48 | 73.6 | 200 | 37700 | 36340 | 14C/AMS 14C | 15 |
| 425 | Bolshaya Balakhnya River | N/A | 100.48 | 73.6 | 600 | 45520 | 43000 | 14C/AMS 14C | 15 |
| 431 | Bolshoi Lyakhovsky Island | N/A | 141.4 | 73.33 | 60 | 14100 | 13740 | 14C/AMS 14C | 15 |
| 431 | Bolshoi Lyakhovsky Island | N/A | 141.4 | 73.33 | 50 | 15050 | 14530 | 14C/AMS 14C | 15 |
| 431 | Bolshoi Lyakhovsky Island | N/A | 141.4 | 73.33 | 1000 | 29030 | 23990 | 14C/AMS 14C | 15 |
| 431 | Bolshoi Lyakhovsky Island | N/A | 141.4 | 73.33 | 600 | 31420 | 29820 | 14C/AMS 14C | 15 |
| 431 | Bolshoi Lyakhovsky Island | N/A | 141.4 | 73.33 | 180 | 33600 | 31280 | 14C/AMS 14C | 15 |
| 431 | Bolshoi Lyakhovsky Island | N/A | 141.4 | 73.33 | 1170 | 40240 | 34800 | 14C/AMS 14C | 15 |
| 431 | Bolshoi Lyakhovsky Island | N/A | 141.4 | 73.33 | 900 | 39840 | 35280 | 14C/AMS 14C | 15 |
| 431 | Bolshoi Lyakhovsky Island | N/A | 141.4 | 73.33 | 500 | 39510 | 36230 | 14C/AMS 14C | 15 |
| 431 | Bolshoi Lyakhovsky Island | N/A | 141.4 | 73.33 | 1000 | 40320 | 35560 | 14C/AMS 14C | 15 |
| 431 | Bolshoi Lyakhovsky Island | N/A | 141.4 | 73.33 | 1000 | 42470 | 38150 | 14C/AMS 14C | 15 |
| 431 | Bolshoi Lyakhovsky Island | N/A | 141.4 | 73.33 | 1800 | 43550 | 36270 | 14C/AMS 14C | 15 |
| 431 | Bolshoi Lyakhovsky Island | N/A | 141.4 | 73.33 | 1600 | 44210 | 38250 | 14C/AMS 14C | 15 |
| 431 | Bolshoi Lyakhovsky Island | N/A | 141.4 | 73.33 | 900 | 43950 | 41550 | 14C/AMS 14C | 15 |
| 431 | Bolshoi Lyakhovsky Island | N/A | 141.4 | 73.33 | 1000 | 45100 | 42220 | 14C/AMS 14C | 15 |
| 431 | Bolshoi Lyakhovsky Island | N/A | 141.4 | 73.33 | 900 | 45450 | 42570 | 14C/AMS 14C | 15 |
| 431 | Bolshoi Lyakhovsky Island | N/A | 141.4 | 73.33 | 1300 | 48980 | 43580 | 14C/AMS 14C | 15 |
| 431 | Bolshoi Lyakhovsky Island | N/A | 141.4 | 73.33 | 1000 | 49450 | 44410 | 14C/AMS 14C | 15 |
| 431 | Bolshoi Lyakhovsky Island | N/A | 141.4 | 73.33 | 2000 | 56380 | 46540 | 14C/AMS 14C | 15 |
| 431 | Bolshoi Lyakhovsky Island | N/A | 141.4 | 73.33 | 1820 | 60170 | 48330 | 14C/AMS 14C | 15 |
| 435 | Bolshoy Khomus-Yuriakh River | N/A | 153.45 | 71.16 | 800 | 47660 | 43980 | 14C/AMS 14C | 15 |
| 438 | Borgo-Tokur River | N/A | 117.01 | 71 | 1240 | 38440 | 32680 | 14C/AMS 14C | 15 |
| 443 | Bur | N/A | 119 | 71.4 | 120 | 22630 | 22070 | 14C/AMS 14C | 15 |
| 445 | Bykovsky Peninsula | N/A | 129.35 | 71.81 | 500 | 17690 | 14130 | 14C/AMS 14C | 15 |
| 445 | Bykovsky Peninsula | N/A | 129.35 | 71.81 | 120 | 28280 | 27480 | 14C/AMS 14C | 15 |
| 445 | Bykovsky Peninsula | N/A | 129.35 | 71.81 | 800 | 35820 | 31020 | 14C/AMS 14C | 15 |
| 445 | Bykovsky Peninsula | N/A | 129.35 | 71.81 | 180 | 34820 | 33100 | 14C/AMS 14C | 15 |
| 445 | Bykovsky Peninsula | N/A | 129.35 | 71.81 | 300 | 39420 | 36380 | 14C/AMS 14C | 15 |
| 445 | Bykovsky Peninsula | N/A | 129.35 | 71.81 | 450 | 39730 | 36650 | 14C/AMS 14C | 15 |
| 452 | Chaidakh-Yuriakh River | N/A | 117.25 | 73.29 | 400 | 35640 | 33440 | 14C/AMS 14C | 15 |
| 453 | Chekurovka | N/A | 127.3 | 71.05 | 1600 | 34570 | 27250 | 14C/AMS 14C | 15 |
| 456 | Chembakchino | N/A | 69.48 | 60 | 180 | 22430 | 21390 | 14C/AMS 14C | 15 |
| 457 | Cheremukhovo 1 | N/A | 60.04 | 60.25 | 320 | 23650 | 21850 | 14C/AMS 14C | 15 |
| 459 | Chersky | N/A | 161.15 | 68.45 | 800 | 49290 | 44650 | 14C/AMS 14C | 15 |
| 462 | Chulym River | N/A | 90 | 55.05 | 100 | 24390 | 23830 | 14C/AMS 14C | 15 |
| 463 | Chulym River | N/A | 90 | 55.05 | 80 | 24190 | 23550 | 14C/AMS 14C | 15 |
| 491 | Druzhinikha | N//A | 93.52 | 56.78 | 8800 | 63120 | 31080 | 14C/AMS 14C | 15 |
| 492 | Dudypta River | N//A | 96.25 | 72.08 | 100 | 24940 | 23940 | 14C/AMS 14C | 15 |
| 493 | Duvanny Yar | N//A | 150.45 | 68.45 | 300 | 34930 | 31850 | 14C/AMS 14C | 15 |
| 493 | Duvanny Yar | N//A | 150.45 | 68.45 | 500 | 42240 | 36840 | 14C/AMS 14C | 15 |
| 493 | Duvanny Yar | N//A | 150.45 | 68.45 | 400 | 42020 | 38940 | 14C/AMS 14C | 15 |
| 494 | Dyagyylakh Sis Island | N//A | 127.17 | 72.32 | 1300 | 58560 | 48960 | 14C/AMS 14C | 15 |
| 495 | Dyuktai Cave, layer 7a | N//A | 132.67 | 59.25 | 250 | 15770 | 13850 | 14C/AMS 14C | 15 |
| 508 | Engelgard Lake | N/A | 110.3 | 75.1 | 100 | 12150 | 11230 | 14C/AMS 14C | 15 |
| 508 | Engelgard Lake | N/A | 110.3 | 75.1 | 800 | 45670 | 42750 | 14C/AMS 14C | 15 |
| 509 | Enmynveem River | N/A | 173.7 | 66.3 | 900 | 38120 | 34520 | 14C/AMS 14C | 15 |
| 509 | Enmynveem River | N/A | 173.7 | 66.3 | 900 | 39170 | 34530 | 14C/AMS 14C | 15 |
| 509 | Enmynveem River | N/A | 173.7 | 66.3 | 3000 | 43600 | 30480 | 14C/AMS 14C | 15 |
| 509 | Enmynveem River | N/A | 173.7 | 66.3 | 720 | 40030 | 36230 | 14C/AMS 14C | 15 |
| 509 | Enmynveem River | N/A | 173.7 | 66.3 | 1200 | 41800 | 35360 | 14C/AMS 14C | 15 |
| 515 | Evalga | N/A | 62.33 | 59.38 | 160 | 20640 | 19800 | 14C/AMS 14C | 15 |
| 515 | Evalga | N/A | 62.33 | 59.38 | 205 | 24250 | 22810 | 14C/AMS 14C | 15 |
| 516 | Faddeevsky Island | N/A | 144 | 75.26 | 120 | 22590 | 21790 | 14C/AMS 14C | 15 |
| 516 | Faddeevsky Island | N/A | 144 | 75.26 | 100 | 25360 | 24400 | 14C/AMS 14C | 15 |
| 516 | Faddeevsky Island | N/A | 144 | 75.26 | 150 | 29450 | 28250 | 14C/AMS 14C | 15 |
| 516 | Faddeevsky Island | N/A | 144 | 75.26 | 150 | 30560 | 29760 | 14C/AMS 14C | 15 |
| 516 | Faddeevsky Island | N/A | 144 | 75.26 | 180 | 30630 | 29710 | 14C/AMS 14C | 15 |
| 516 | Faddeevsky Island | N/A | 144 | 75.26 | 170 | 30860 | 30180 | 14C/AMS 14C | 15 |
| 516 | Faddeevsky Island | N/A | 144 | 75.26 | 200 | 31070 | 30270 | 14C/AMS 14C | 15 |
| 516 | Faddeevsky Island | N/A | 144 | 75.26 | 300 | 31830 | 31030 | 14C/AMS 14C | 15 |
| 516 | Faddeevsky Island | N/A | 144 | 75.26 | 200 | 33650 | 31250 | 14C/AMS 14C | 15 |
| 516 | Faddeevsky Island | N/A | 144 | 75.26 | 350 | 35120 | 31800 | 14C/AMS 14C | 15 |
| 516 | Faddeevsky Island | N/A | 144 | 75.26 | 400 | 35470 | 32950 | 14C/AMS 14C | 15 |
| 516 | Faddeevsky Island | N/A | 144 | 75.26 | 1000 | 36460 | 31060 | 14C/AMS 14C | 15 |
| 516 | Faddeevsky Island | N/A | 144 | 75.26 | 250 | 35640 | 34280 | 14C/AMS 14C | 15 |
| 516 | Faddeevsky Island | N/A | 144 | 75.26 | 300 | 37080 | 35760 | 14C/AMS 14C | 15 |
| 516 | Faddeevsky Island | N/A | 144 | 75.26 | 500 | 41970 | 38770 | 14C/AMS 14C | 15 |
| 516 | Faddeevsky Island | N/A | 144 | 75.26 | 500 | 42450 | 39010 | 14C/AMS 14C | 15 |
| 516 | Faddeevsky Island | N/A | 144 | 75.26 | 500 | 42660 | 40220 | 14C/AMS 14C | 15 |
| 516 | Faddeevsky Island | N/A | 144 | 75.26 | 500 | 42650 | 41490 | 14C/AMS 14C | 15 |
| 518 | Farvaternyi Island, Pyasina River | N/A | 85.75 | 73.95 | 700 | 39780 | 36100 | 14C/AMS 14C | 15 |
| 527 | Gari | N/A | 62.34 | 59.24 | 280 | 18950 | 17910 | 14C/AMS 14C | 15 |
| 527 | Gari | N/A | 62.34 | 59.24 | 450 | 20620 | 18620 | 14C/AMS 14C | 15 |
| 527 | Gari | N/A | 62.34 | 59.24 | 240 | 20550 | 19350 | 14C/AMS 14C | 15 |
| 529 | Geographical Society Cave | N/A | 133 | 42.87 | 980 | 39540 | 34500 | 14C/AMS 14C | 15 |
| 529 | Geographical Society Cave | N/A | 133 | 42.87 | 600 | 38270 | 35310 | 14C/AMS 14C | 15 |
| 529 | Geographical Society Cave | N/A | 133 | 42.87 | 1510 | 41930 | 34570 | 14C/AMS 14C | 15 |
| 529 | Geographical Society Cave | N/A | 133 | 42.87 | 1000 | 41570 | 35730 | 14C/AMS 14C | 15 |
| 529 | Geographical Society Cave | N/A | 133 | 42.87 | 60 | 39980 | 36780 | 14C/AMS 14C | 15 |
| 531 | Gofman Cape | N/A | 100.5 | 74.88 | 2000 | 48090 | 41650 | 14C/AMS 14C | 15 |
| 532 | Goltsovaya River | N/A | 104.58 | 76.8 | 40 | 12160 | 11760 | 14C/AMS 14C | 15 |
| 535 | Gorki | N/A | 65.12 | 65.1 | 675 | 39910 | 36310 | 14C/AMS 14C | 15 |
| 576 | Gyda River | N/A | 77.3 | 70.3 | 1800 | 39570 | 30730 | 14C/AMS 14C | 15 |
| 576 | Gyda River | N/A | 77.3 | 70.3 | 1000 | 42230 | 35990 | 14C/AMS 14C | 15 |
| 576 | Gyda River | N/A | 77.3 | 70.3 | 1100 | 42930 | 38490 | 14C/AMS 14C | 15 |
| 588 | Igolskoe | N/A | 80.28 | 57.13 | 390 | 31170 | 30010 | 14C/AMS 14C | 15 |
| 593 | Iritka River | N/A | 104.67 | 63.55 | 1400 | 41400 | 34640 | 14C/AMS 14C | 15 |
| 594 | Irkutsk | N/A | 104.4 | 53 | 2015 | 36830 | 28550 | 14C/AMS 14C | 15 |
| 595 | Irtysh River | N/A | 69.3 | 58.03 | 100 | 17030 | 15350 | 14C/AMS 14C | 15 |
| 596 | Irtysh River, Yugoyar Berezovsky | N/A | 69 | 59 | 90 | 16040 | 14960 | 14C/AMS 14C | 15 |
| 597 | Isha River | N/A | 86.32 | 52.01 | 245 | 21730 | 19770 | 14C/AMS 14C | 15 |
| 597 | Isha River | N/A | 86.32 | 52.01 | 500 | 22310 | 19870 | 14C/AMS 14C | 15 |
| 598 | Ishim River | N/A | 70.48 | 57.4 | 840 | 44400 | 41840 | 14C/AMS 14C | 15 |
| 601 | Kamchatka River, Generalka | N/A | 159.15 | 55 | 150 | 27280 | 25480 | 14C/AMS 14C | 15 |
| 602 | Kamchatka River, Nikolka | N/A | 159 | 55 | 500 | 42660 | 40220 | 14C/AMS 14C | 15 |
| 603 | Kamchatka River, Polovinka | N/A | 159 | 55 | 600 | 45520 | 43000 | 14C/AMS 14C | 15 |
| 604 | Kamchatka River, Urz | N/A | 159 | 55 | 50 | 15260 | 14660 | 14C/AMS 14C | 15 |
| 606 | Kamenka 1, unit A | N/A | 108.15 | 51.87 | 530 | 37120 | 35200 | 14C/AMS 14C | 15 |
| 608 | Kamtchatka River, Yar Bolshoi | N/A | 159.75 | 56 | 300 | 35960 | 34640 | 14C/AMS 14C | 15 |
| 610 | Kandabaevo | N/A | 108.48 | 50.9 | 1100 | 44610 | 41690 | 14C/AMS 14C | 15 |
| 612 | Katun River | N/A | 85.35 | 51.43 | 995 | 36110 | 30830 | 14C/AMS 14C | 15 |
| 614 | Kaverga River | N/A | 112.25 | 57.23 | 100 | 21050 | 20250 | 14C/AMS 14C | 15 |
| 614 | Kaverga River | N/A | 112.25 | 57.23 | 730 | 30950 | 27910 | 14C/AMS 14C | 15 |
| 614 | Kaverga River | N/A | 112.25 | 57.23 | 1600 | 55580 | 46580 | 14C/AMS 14C | 15 |
| 615 | Keinguveem River | N/A | 164.8 | 69.6 | 420 | 37380 | 35700 | 14C/AMS 14C | 15 |
| 615 | Keinguveem River | N/A | 164.8 | 69.6 | 900 | 44470 | 41830 | 14C/AMS 14C | 15 |
| 627 | Khatanga River | N/A | 103 | 72.16 | 1500 | 44800 | 40960 | 14C/AMS 14C | 15 |
| 628 | Khayrgas | 6 | 133 | 62.42 | 300 | 19760 | 18680 | 14C/AMS 14C | 15 |
| 629 | Khayrgas | 7 | 133 | 62.42 | 775 | 28010 | 23770 | 14C/AMS 14C | 15 |
| 630 | Kheta River | N/A | 97 | 70.83 | 1000 | 50710 | 45430 | 14C/AMS 14C | 15 |
| 632 | Kholodnaya River | N/A | 109.75 | 55.88 | 480 | 34390 | 31030 | 14C/AMS 14C | 15 |
| 633 | Khomus-Yuriakh River | N/A | 153.45 | 71.16 | 300 | 36240 | 35040 | 14C/AMS 14C | 15 |
| 634 | Khorol | N/A | 131.78 | 44.15 | 780 | 18830 | 14230 | 14C/AMS 14C | 15 |
| 634 | Khorol | N/A | 131.78 | 44.15 | 140 | 18980 | 18100 | 14C/AMS 14C | 15 |
| 634 | Khorol | N/A | 131.78 | 44.15 | 150 | 21710 | 20150 | 14C/AMS 14C | 15 |
| 635 | Khotyk | N/A | 109.83 | 52.22 | 245 | 34970 | 32690 | 14C/AMS 14C | 15 |
| 640 | Kimitina River | N/A | 160 | 56.3 | 1600 | 45930 | 41690 | 14C/AMS 14C | 15 |
| 645 | Kirgilyakh River | N/A | 158.3 | 68.45 | 870 | 44960 | 42320 | 14C/AMS 14C | 15 |
| 645 | Kirgilyakh River | N/A | 158.3 | 68.45 | 770 | 44880 | 42400 | 14C/AMS 14C | 15 |
| 645 | Kirgilyakh River | N/A | 158.3 | 68.45 | 700 | 45630 | 42910 | 14C/AMS 14C | 15 |
| 645 | Kirgilyakh River | N/A | 158.3 | 68.45 | 700 | 45630 | 42910 | 14C/AMS 14C | 15 |
| 645 | Kirgilyakh River | N/A | 158.3 | 68.45 | 900 | 46280 | 42960 | 14C/AMS 14C | 15 |
| 645 | Kirgilyakh River | N/A | 158.3 | 68.45 | 1100 | 46630 | 42710 | 14C/AMS 14C | 15 |
| 647 | Kirpichny | N/A | 68.41 | 61.04 | 105 | 17240 | 16600 | 14C/AMS 14C | 15 |
| 647 | Kirpichny | N/A | 68.41 | 61.04 | 350 | 19600 | 18320 | 14C/AMS 14C | 15 |
| 649 | Kiya River | N/A | 88 | 55.64 | 200 | 27960 | 26000 | 14C/AMS 14C | 15 |
| 652 | Kochegur | N/A | 88.05 | 55.85 | 240 | 22730 | 21650 | 14C/AMS 14C | 15 |
| 652 | Kochegur | N/A | 88.05 | 55.85 | 220 | 23690 | 22370 | 14C/AMS 14C | 15 |
| 652 | Kochegur | N/A | 88.05 | 55.85 | 400 | 25190 | 23150 | 14C/AMS 14C | 15 |
| 654 | Kolyma_River | N/A | 157.67 | 68.2 | 1000 | 52490 | 46130 | 14C/AMS 14C | 15 |
| 656 | Komsomolsky | N/A | 63.57 | 57.07 | 340 | 23700 | 22020 | 14C/AMS 14C | 15 |
| 657 | Kondratyeva | N/A | 141.66 | 72.68 | 300 | 21510 | 19470 | 14C/AMS 14C | 15 |
| 657 | Kondratyeva | N/A | 141.66 | 72.68 | 600 | 31510 | 29990 | 14C/AMS 14C | 15 |
| 657 | Kondratyeva | N/A | 141.66 | 72.68 | 400 | 42450 | 41450 | 14C/AMS 14C | 15 |
| 657 | Kondratyeva | N/A | 141.66 | 72.68 | 400 | 42850 | 41730 | 14C/AMS 14C | 15 |
| 657 | Kondratyeva | N/A | 141.66 | 72.68 | 1000 | 48910 | 44110 | 14C/AMS 14C | 15 |
| 657 | Kondratyeva | N/A | 141.66 | 72.68 | 1200 | 55060 | 47060 | 14C/AMS 14C | 15 |
| 658 | Konzhul | N/A | 92.47 | 55.33 | 155 | 14450 | 13490 | 14C/AMS 14C | 15 |
| 658 | Konzhul | N/A | 92.47 | 55.33 | 175 | 16700 | 14660 | 14C/AMS 14C | 15 |
| 671 | Kotelny Island | N/A | 138 | 75 | 100 | 17300 | 17060 | 14C/AMS 14C | 15 |
| 671 | Kotelny Island | N/A | 138 | 75 | 100 | 18940 | 18540 | 14C/AMS 14C | 15 |
| 671 | Kotelny Island | N/A | 138 | 75 | 110 | 24230 | 23550 | 14C/AMS 14C | 15 |
| 671 | Kotelny Island | N/A | 138 | 75 | 190 | 35120 | 33400 | 14C/AMS 14C | 15 |
| 671 | Kotelny Island | N/A | 138 | 75 | 400 | 41880 | 38800 | 14C/AMS 14C | 15 |
| 675 | Krasnaya River | N/A | 98.5 | 74.57 | 100 | 12930 | 12610 | 14C/AMS 14C | 15 |
| 676 | Krasny Yar, Tomsk Province | N/A | 84.45 | 57.13 | 420 | 31170 | 29930 | 14C/AMS 14C | 15 |
| 678 | Kubalakh River | N/A | 97.88 | 73.28 | 300 | 33090 | 30890 | 14C/AMS 14C | 15 |
| 679 | Kuda River | N/A | 104.49 | 52.22 | 400 | 32410 | 30810 | 14C/AMS 14C | 15 |
| 680 | Kudelin Klyuch | N/A | 84.24 | 55.12 | 245 | 29390 | 27990 | 14C/AMS 14C | 15 |
| 681 | Kuilinsky Yar | N/A | 86.17 | 56.75 | 540 | 35690 | 31890 | 14C/AMS 14C | 15 |
| 682 | Kulachye | N/A | 73.15 | 55.12 | 385 | 22270 | 20150 | 14C/AMS 14C | 15 |
| 683 | Kular | N/A | 134.23 | 70.5 | 1100 | 31120 | 26400 | 14C/AMS 14C | 15 |
| 686 | Kumyshanskaya Cave | N/A | 58.22 | 58.15 | 260 | 15590 | 13710 | 14C/AMS 14C | 15 |
| 687 | Kupchiktakh Lake | N/A | 101.13 | 73.6 | 40 | 12090 | 11730 | 14C/AMS 14C | 15 |
| 687 | Kupchiktakh Lake | N/A | 101.13 | 73.6 | 800 | 39760 | 35520 | 14C/AMS 14C | 15 |
| 688 | Kuznetsky Basin | N/A | 86.38 | 54.12 | 600 | 35730 | 31530 | 14C/AMS 14C | 15 |
| 688 | Kuznetsky Basin | N/A | 86.38 | 54.12 | 700 | 44300 | 41940 | 14C/AMS 14C | 15 |
| 688 | Kuznetsky Basin | N/A | 86.38 | 54.12 | 2440 | 47020 | 40140 | 14C/AMS 14C | 15 |
| 688 | Kuznetsky Basin | N/A | 86.38 | 54.12 | 1000 | 47270 | 43510 | 14C/AMS 14C | 15 |
| 688 | Kuznetsky Basin | N/A | 86.38 | 54.12 | 2720 | 57530 | 45410 | 14C/AMS 14C | 15 |
| 698 | Labaz Lake | N/A | 99.7 | 72.37 | 110 | 30310 | 29350 | 14C/AMS 14C | 15 |
| 707 | Lena River Delta, Bykovskaya Channel | N/A | 129.25 | 71.8 | 2500 | 43890 | 34690 | 14C/AMS 14C | 15 |
| 707 | Lena River Delta, Bykovskaya Channel | N/A | 129.25 | 71.8 | 1200 | 43230 | 38430 | 14C/AMS 14C | 15 |
| 708 | Lena River Delta, Olenyok Channel | N/A | 123.35 | 72.9 | 400 | 36230 | 34710 | 14C/AMS 14C | 15 |
| 718 | Listvenka | N/A | 92.33 | 55.92 | 600 | 20860 | 18380 | 14C/AMS 14C | 15 |
| 719 | Listvenka | 12G | 92.33 | 55.92 | 150 | 17260 | 15300 | 14C/AMS 14C | 15 |
| 719 | Listvenka | 12G | 92.33 | 55.92 | 160 | 17380 | 15340 | 14C/AMS 14C | 15 |
| 720 | Listvenka | 19 | 92.33 | 55.92 | 230 | 21690 | 19730 | 14C/AMS 14C | 15 |
| 721 | Listvenka | 20 | 92.33 | 55.92 | 380 | 25580 | 23740 | 14C/AMS 14C | 15 |
| 725 | Logata River | N/A | 97 | 73.35 | 200 | 31890 | 31130 | 14C/AMS 14C | 15 |
| 725 | Logata River | N/A | 97 | 73.35 | 500 | 42300 | 38940 | 14C/AMS 14C | 15 |
| 725 | Logata River | N/A | 97 | 73.35 | 500 | 42460 | 41180 | 14C/AMS 14C | 15 |
| 725 | Logata River | N/A | 97 | 73.35 | 600 | 44020 | 41940 | 14C/AMS 14C | 15 |
| 725 | Logata River | N/A | 97 | 73.35 | 600 | 44470 | 42150 | 14C/AMS 14C | 15 |
| 725 | Logata River | N/A | 97 | 73.35 | 600 | 45120 | 42840 | 14C/AMS 14C | 15 |
| 727 | Lopatka Peninsula | N/A | 150 | 71.83 | 200 | 31360 | 30960 | 14C/AMS 14C | 15 |
| 727 | Lopatka Peninsula | N/A | 150 | 71.83 | 200 | 34780 | 32580 | 14C/AMS 14C | 15 |
| 727 | Lopatka Peninsula | N/A | 150 | 71.83 | 300 | 35900 | 34460 | 14C/AMS 14C | 15 |
| 727 | Lopatka Peninsula | N/A | 150 | 71.83 | 500 | 41990 | 36310 | 14C/AMS 14C | 15 |
| 729 | Lower Lena River | N/A | 125 | 70 | 80 | 21820 | 20700 | 14C/AMS 14C | 15 |
| 729 | Lower Lena River | N/A | 125 | 70 | 310 | 26320 | 24640 | 14C/AMS 14C | 15 |
| 732 | Lugovskoe | N/A | 68.57 | 61.05 | 135 | 12550 | 11310 | 14C/AMS 14C | 15 |
| 732 | Lugovskoe | N/A | 68.57 | 61.05 | 170 | 13100 | 12500 | 14C/AMS 14C | 15 |
| 732 | Lugovskoe | N/A | 68.57 | 61.05 | 380 | 13990 | 12510 | 14C/AMS 14C | 15 |
| 732 | Lugovskoe | N/A | 68.57 | 61.05 | 95 | 13950 | 13510 | 14C/AMS 14C | 15 |
| 732 | Lugovskoe | N/A | 68.57 | 61.05 | 350 | 17250 | 14010 | 14C/AMS 14C | 15 |
| 732 | Lugovskoe | N/A | 68.57 | 61.05 | 160 | 16890 | 14890 | 14C/AMS 14C | 15 |
| 732 | Lugovskoe | N/A | 68.57 | 61.05 | 60 | 17110 | 15510 | 14C/AMS 14C | 15 |
| 732 | Lugovskoe | N/A | 68.57 | 61.05 | 50 | 17190 | 16790 | 14C/AMS 14C | 15 |
| 732 | Lugovskoe | N/A | 68.57 | 61.05 | 60 | 17190 | 16790 | 14C/AMS 14C | 15 |
| 732 | Lugovskoe | N/A | 68.57 | 61.05 | 50 | 17190 | 16830 | 14C/AMS 14C | 15 |
| 732 | Lugovskoe | N/A | 68.57 | 61.05 | 155 | 17410 | 16250 | 14C/AMS 14C | 15 |
| 732 | Lugovskoe | N/A | 68.57 | 61.05 | 160 | 17370 | 16930 | 14C/AMS 14C | 15 |
| 732 | Lugovskoe | N/A | 68.57 | 61.05 | 215 | 19140 | 18180 | 14C/AMS 14C | 15 |
| 733 | Lugovskoye | N/A | 68.57 | 61.05 | 1100 | 24500 | 19220 | 14C/AMS 14C | 15 |
| 735 | Lygiy-Yuryue River | N/A | 116.02 | 73.58 | 300 | 33090 | 30890 | 14C/AMS 14C | 15 |
| 737 | Lyzhin Mys | N/A | 62.25 | 59.5 | 220 | 25150 | 24070 | 14C/AMS 14C | 15 |
| 746 | Malta | N/A | 103.5 | 53 | 300 | 24260 | 22500 | 14C/AMS 14C | 15 |
| 746 | Malta | N/A | 103.5 | 53 | 800 | 25770 | 21890 | 14C/AMS 14C | 15 |
| 746 | Malta | N/A | 103.5 | 53 | 240 | 25080 | 23840 | 14C/AMS 14C | 15 |
| 746 | Malta | N/A | 103.5 | 53 | 120 | 25190 | 24270 | 14C/AMS 14C | 15 |
| 746 | Malta | N/A | 103.5 | 53 | 140 | 25220 | 24260 | 14C/AMS 14C | 15 |
| 746 | Malta | N/A | 103.5 | 53 | 200 | 25370 | 24170 | 14C/AMS 14C | 15 |
| 746 | Malta | N/A | 103.5 | 53 | 200 | 25760 | 24160 | 14C/AMS 14C | 15 |
| 746 | Malta | N/A | 103.5 | 53 | 110 | 25630 | 24710 | 14C/AMS 14C | 15 |
| 746 | Malta | N/A | 103.5 | 53 | 140 | 25720 | 24640 | 14C/AMS 14C | 15 |
| 746 | Malta | N/A | 103.5 | 53 | 250 | 26020 | 24260 | 14C/AMS 14C | 15 |
| 746 | Malta | N/A | 103.5 | 53 | 150 | 25870 | 24750 | 14C/AMS 14C | 15 |
| 746 | Malta | N/A | 103.5 | 53 | 110 | 26070 | 24910 | 14C/AMS 14C | 15 |
| 746 | Malta | N/A | 103.5 | 53 | 200 | 26180 | 24820 | 14C/AMS 14C | 15 |
| 746 | Malta | N/A | 103.5 | 53 | 300 | 26350 | 24670 | 14C/AMS 14C | 15 |
| 746 | Malta | N/A | 103.5 | 53 | 110 | 26190 | 24990 | 14C/AMS 14C | 15 |
| 746 | Malta | N/A | 103.5 | 53 | 170 | 26570 | 25210 | 14C/AMS 14C | 15 |
| 746 | Malta | N/A | 103.5 | 53 | 200 | 27040 | 25000 | 14C/AMS 14C | 15 |
| 746 | Malta | N/A | 103.5 | 53 | 1500 | 47500 | 42300 | 14C/AMS 14C | 15 |
| 747 | Maly Anui River | N/A | 161.5 | 68.33 | 310 | 33070 | 30870 | 14C/AMS 14C | 15 |
| 748 | Maly Oesh River | N/A | 82.63 | 55.3 | 160 | 19690 | 18970 | 14C/AMS 14C | 15 |
| 752 | Mamontovaya Khayata | N/A | 129.42 | 71.78 | 170 | 16460 | 14460 | 14C/AMS 14C | 15 |
| 752 | Mamontovaya Khayata | N/A | 129.42 | 71.78 | 80 | 17630 | 17310 | 14C/AMS 14C | 15 |
| 752 | Mamontovaya Khayata | N/A | 129.42 | 71.78 | 60 | 17780 | 17380 | 14C/AMS 14C | 15 |
| 752 | Mamontovaya Khayata | N/A | 129.42 | 71.78 | 280 | 18240 | 17160 | 14C/AMS 14C | 15 |
| 752 | Mamontovaya Khayata | N/A | 129.42 | 71.78 | 110 | 18150 | 17430 | 14C/AMS 14C | 15 |
| 752 | Mamontovaya Khayata | N/A | 129.42 | 71.78 | 140 | 18180 | 17380 | 14C/AMS 14C | 15 |
| 752 | Mamontovaya Khayata | N/A | 129.42 | 71.78 | 100 | 18170 | 17570 | 14C/AMS 14C | 15 |
| 752 | Mamontovaya Khayata | N/A | 129.42 | 71.78 | 60 | 18200 | 17800 | 14C/AMS 14C | 15 |
| 752 | Mamontovaya Khayata | N/A | 129.42 | 71.78 | 100 | 24390 | 23830 | 14C/AMS 14C | 15 |
| 752 | Mamontovaya Khayata | N/A | 129.42 | 71.78 | 600 | 26540 | 23420 | 14C/AMS 14C | 15 |
| 752 | Mamontovaya Khayata | N/A | 129.42 | 71.78 | 240 | 27320 | 24920 | 14C/AMS 14C | 15 |
| 752 | Mamontovaya Khayata | N/A | 129.42 | 71.78 | 1200 | 30650 | 24730 | 14C/AMS 14C | 15 |
| 752 | Mamontovaya Khayata | N/A | 129.42 | 71.78 | 200 | 29890 | 28490 | 14C/AMS 14C | 15 |
| 752 | Mamontovaya Khayata | N/A | 129.42 | 71.78 | 90 | 30340 | 29380 | 14C/AMS 14C | 15 |
| 752 | Mamontovaya Khayata | N/A | 129.42 | 71.78 | 300 | 30600 | 29280 | 14C/AMS 14C | 15 |
| 752 | Mamontovaya Khayata | N/A | 129.42 | 71.78 | 230 | 31330 | 30810 | 14C/AMS 14C | 15 |
| 752 | Mamontovaya Khayata | N/A | 129.42 | 71.78 | 900 | 33720 | 29600 | 14C/AMS 14C | 15 |
| 752 | Mamontovaya Khayata | N/A | 129.42 | 71.78 | 800 | 34320 | 30360 | 14C/AMS 14C | 15 |
| 752 | Mamontovaya Khayata | N/A | 129.42 | 71.78 | 900 | 35450 | 30650 | 14C/AMS 14C | 15 |
| 752 | Mamontovaya Khayata | N/A | 129.42 | 71.78 | 200 | 34980 | 33220 | 14C/AMS 14C | 15 |
| 752 | Mamontovaya Khayata | N/A | 129.42 | 71.78 | 600 | 35970 | 32770 | 14C/AMS 14C | 15 |
| 752 | Mamontovaya Khayata | N/A | 129.42 | 71.78 | 600 | 36670 | 34310 | 14C/AMS 14C | 15 |
| 752 | Mamontovaya Khayata | N/A | 129.42 | 71.78 | 900 | 37470 | 33870 | 14C/AMS 14C | 15 |
| 752 | Mamontovaya Khayata | N/A | 129.42 | 71.78 | 650 | 38470 | 35150 | 14C/AMS 14C | 15 |
| 752 | Mamontovaya Khayata | N/A | 129.42 | 71.78 | 800 | 40220 | 36100 | 14C/AMS 14C | 15 |
| 752 | Mamontovaya Khayata | N/A | 129.42 | 71.78 | 300 | 41740 | 36380 | 14C/AMS 14C | 15 |
| 752 | Mamontovaya Khayata | N/A | 129.42 | 71.78 | 260 | 41870 | 38070 | 14C/AMS 14C | 15 |
| 752 | Mamontovaya Khayata | N/A | 129.42 | 71.78 | 500 | 41960 | 38120 | 14C/AMS 14C | 15 |
| 752 | Mamontovaya Khayata | N/A | 129.42 | 71.78 | 320 | 41860 | 38780 | 14C/AMS 14C | 15 |
| 752 | Mamontovaya Khayata | N/A | 129.42 | 71.78 | 400 | 42180 | 38980 | 14C/AMS 14C | 15 |
| 752 | Mamontovaya Khayata | N/A | 129.42 | 71.78 | 500 | 42610 | 39250 | 14C/AMS 14C | 15 |
| 752 | Mamontovaya Khayata | N/A | 129.42 | 71.78 | 700 | 42820 | 39140 | 14C/AMS 14C | 15 |
| 752 | Mamontovaya Khayata | N/A | 129.42 | 71.78 | 1000 | 50320 | 45000 | 14C/AMS 14C | 15 |
| 752 | Mamontovaya Khayata | N/A | 129.42 | 71.78 | 1100 | 50440 | 44880 | 14C/AMS 14C | 15 |
| 754 | Massonov River | N/A | 100 | 72.89 | 800 | 48170 | 44170 | 14C/AMS 14C | 15 |
| 755 | Matuda Peninsula | N/A | 102.63 | 74.5 | 300 | 35580 | 33820 | 14C/AMS 14C | 15 |
| 755 | Matuda Peninsula | N/A | 102.63 | 74.5 | 500 | 39400 | 35600 | 14C/AMS 14C | 15 |
| 756 | Maymecha River | N/A | 101 | 70.83 | 700 | 32870 | 30030 | 14C/AMS 14C | 15 |
| 756 | Maymecha River | N/A | 101 | 70.83 | 1100 | 57210 | 48730 | 14C/AMS 14C | 15 |
| 757 | Mayn River | N/A | 171 | 65 | 70 | 18690 | 18090 | 14C/AMS 14C | 15 |
| 762 | Middle Angara River | N/A | 101.3 | 59 | 200 | 29000 | 27920 | 14C/AMS 14C | 15 |
| 763 | Middle Yenisei River | N/A | 92 | 53.55 | 200 | 23890 | 22610 | 14C/AMS 14C | 15 |
| 763 | Middle Yenisei River | N/A | 92 | 53.55 | 200 | 24230 | 22790 | 14C/AMS 14C | 15 |
| 763 | Middle Yenisei River | N/A | 92 | 53.55 | 300 | 24760 | 23320 | 14C/AMS 14C | 15 |
| 769 | Mingyuegou | N/A | 128.91 | 43.11 | 550 | 31760 | 30360 | 14C/AMS 14C | 15 |
| 769 | Mingyuegou | N/A | 128.91 | 43.11 | 1850 | 43750 | 36470 | 14C/AMS 14C | 15 |
| 770 | Mogochino | N/A | 83.52 | 57.75 | 240 | 24660 | 23460 | 14C/AMS 14C | 15 |
| 771 | Mokhovaya River | N/A | 85.3 | 72 | 2700 | 45180 | 35220 | 14C/AMS 14C | 15 |
| 771 | Mokhovaya River | N/A | 85.3 | 72 | 4300 | 48780 | 33260 | 14C/AMS 14C | 15 |
| 783 | Mudanjiang | N/A | 129.58 | 44.63 | 1000 | 27880 | 22560 | 14C/AMS 14C | 15 |
| 784 | Munchirdakh Lake | N/A | 110.5 | 73 | 80 | 29640 | 28640 | 14C/AMS 14C | 15 |
| 786 | Mus-Khaya | N/A | 135.33 | 70.08 | 470 | 41980 | 38860 | 14C/AMS 14C | 15 |
| 786 | Mus-Khaya | N/A | 135.33 | 70.08 | 2000 | 43950 | 36190 | 14C/AMS 14C | 15 |
| 791 | Nemu-Dika-Tarida River | N/A | 98.75 | 73.08 | 400 | 44280 | 42200 | 14C/AMS 14C | 15 |
| 796 | Nganasanskaya River | N/A | 99.41 | 74.4 | 70 | 12790 | 12590 | 14C/AMS 14C | 15 |
| 797 | Nikolskaya Cave | N/A | 59.29 | 55.29 | 310 | 20070 | 18710 | 14C/AMS 14C | 15 |
| 799 | Niryakyan River | N/A | 111.83 | 57.22 | 100 | 21700 | 20180 | 14C/AMS 14C | 15 |
| 799 | Niryakyan River | N/A | 111.83 | 57.22 | 100 | 24940 | 23940 | 14C/AMS 14C | 15 |
| 800 | Nizhnaya Taimyra River | N/A | 99.73 | 75.25 | 60 | 11320 | 10720 | 14C/AMS 14C | 15 |
| 800 | Nizhnaya Taimyra River | N/A | 99.73 | 75.25 | 50 | 11380 | 11180 | 14C/AMS 14C | 15 |
| 800 | Nizhnaya Taimyra River | N/A | 99.73 | 75.25 | 100 | 12630 | 11670 | 14C/AMS 14C | 15 |
| 802 | Nizhneirtyshskoe | N/A | 73.14 | 55.26 | 130 | 17330 | 16850 | 14C/AMS 14C | 15 |
| 802 | Nizhneirtyshskoe | N/A | 73.14 | 55.26 | 195 | 18230 | 17350 | 14C/AMS 14C | 15 |
| 805 | Nizhny Tagil | N/A | 59.67 | 57.92 | 520 | 41590 | 36310 | 14C/AMS 14C | 15 |
| 806 | Nizhnyaya Tavda | N/A | 60.12 | 57.41 | 660 | 24100 | 20820 | 14C/AMS 14C | 15 |
| 809 | Northeastern Point, St. Paul Island | N/A | 188.35 | 57.17 | 100 | 9080 | 8480 | 14C/AMS 14C | 15 |
| 809 | Northeastern Point, St. Paul Island | N/A | 188.35 | 57.17 | 40 | 9070 | 8710 | 14C/AMS 14C | 15 |
| 809 | Northeastern Point, St. Paul Island | N/A | 188.35 | 57.17 | 85 | 9130 | 8610 | 14C/AMS 14C | 15 |
| 810 | Notsukesaki 1 | N/A | 145.38 | 43.62 | 120 | 25170 | 24250 | 14C/AMS 14C | 15 |
| 812 | Novaya River, Markel Woolly mammoth | N/A | 101 | 72.75 | 280 | 39420 | 36500 | 14C/AMS 14C | 15 |
| 812 | Novaya River, Markel Woolly mammoth | N/A | 101 | 72.75 | 1190 | 47400 | 43080 | 14C/AMS 14C | 15 |
| 813 | Novaya Sibir Island | N/A | 148 | 75 | 60 | 11300 | 10700 | 14C/AMS 14C | 15 |
| 815 | Novoaleksandrovka | N/A | 128.27 | 50.03 | 4000 | 52390 | 38670 | 14C/AMS 14C | 15 |
| 817 | Nyengatiatari River | N/A | 106.17 | 74.83 | 60 | 11930 | 11290 | 14C/AMS 14C | 15 |
| 818 | Nyunkarakutari River | N/A | 105.5 | 75.35 | 120 | 12640 | 11520 | 14C/AMS 14C | 15 |
| 821 | Obzhorinsky Yar | N/A | 91.06 | 57.11 | 185 | 17480 | 15920 | 14C/AMS 14C | 15 |
| 823 | Ogoshi | N/A | 143.2 | 41.97 | 80 | 23860 | 22820 | 14C/AMS 14C | 15 |
| 824 | Oktyabrskoi Revolutsii Island | N/A | 97.45 | 79.15 | 110 | 12760 | 11960 | 14C/AMS 14C | 15 |
| 824 | Oktyabrskoi Revolutsii Island | N/A | 97.45 | 79.15 | 60 | 13590 | 13230 | 14C/AMS 14C | 15 |
| 824 | Oktyabrskoi Revolutsii Island | N/A | 97.45 | 79.15 | 330 | 24340 | 22500 | 14C/AMS 14C | 15 |
| 824 | Oktyabrskoi Revolutsii Island | N/A | 97.45 | 79.15 | 110 | 24210 | 23530 | 14C/AMS 14C | 15 |
| 824 | Oktyabrskoi Revolutsii Island | N/A | 97.45 | 79.15 | 210 | 30490 | 29370 | 14C/AMS 14C | 15 |
| 825 | Oktyabrsky | N/A | 128.68 | 53 | 1170 | 45110 | 41990 | 14C/AMS 14C | 15 |
| 827 | OLP | N/A | 62.25 | 59.16 | 160 | 13320 | 12680 | 14C/AMS 14C | 15 |
| 829 | Onot River | N/A | 102 | 52.72 | 155 | 26320 | 25120 | 14C/AMS 14C | 15 |
| 829 | Onot River | N/A | 102 | 52.72 | 150 | 27410 | 25930 | 14C/AMS 14C | 15 |
| 836 | Ozernaya Balya | N/A | 103.05 | 55.45 | 780 | 39600 | 35120 | 14C/AMS 14C | 15 |
| 837 | Pakhcha River | N/A | 161 | 56.35 | 400 | 26490 | 24330 | 14C/AMS 14C | 15 |
| 837 | Pakhcha River | N/A | 161 | 56.35 | 200 | 26180 | 24820 | 14C/AMS 14C | 15 |
| 837 | Pakhcha River | N/A | 161 | 56.35 | 600 | 27500 | 23860 | 14C/AMS 14C | 15 |
| 838 | Parisento River | N/A | 75.46 | 70.11 | 300 | 21850 | 20130 | 14C/AMS 14C | 15 |
| 843 | Pichuguy-Yaha | N/A | 69.17 | 66.58 | 70 | 18950 | 18590 | 14C/AMS 14C | 15 |
| 843 | Pichuguy-Yaha | N/A | 69.17 | 66.58 | 220 | 30550 | 29430 | 14C/AMS 14C | 15 |
| 859 | Pirkanaivayam River | N/A | 166 | 68.03 | 900 | 40720 | 35920 | 14C/AMS 14C | 15 |
| 862 | Podaik | N/A | 87.9 | 56.03 | 190 | 21700 | 20020 | 14C/AMS 14C | 15 |
| 864 | Popigai River | N/A | 106 | 72.4 | 200 | 27400 | 25800 | 14C/AMS 14C | 15 |
| 864 | Popigai River | N/A | 106 | 72.4 | 400 | 39460 | 36060 | 14C/AMS 14C | 15 |
| 866 | Popigay River | N/A | 109 | 72.5 | 460 | 42820 | 41660 | 14C/AMS 14C | 15 |
| 866 | Popigay River | N/A | 109 | 72.5 | 1650 | 55720 | 46720 | 14C/AMS 14C | 15 |
| 872 | Prichulymskoe | N/A | 85.1 | 55.72 | 165 | 19090 | 18530 | 14C/AMS 14C | 15 |
| 874 | Pronchishcheva Bay | N/A | 113 | 76 | 40 | 11270 | 11150 | 14C/AMS 14C | 15 |
| 876 | Pyak-Yaha | N/A | 69.12 | 66.63 | 210 | 42370 | 39290 | 14C/AMS 14C | 15 |
| 877 | Pyasina River | N/A | 87 | 72.5 | 500 | 31000 | 29000 | 14C/AMS 14C | 15 |
| 878 | Qagnax Cave, St. Paul Island | N/A | -171.65 | 57.17 | 40 | 6500 | 6300 | 14C/AMS 14C | 15 |
| 878 | Qagnax Cave, St. Paul Island | N/A | -171.65 | 57.17 | 60 | 6680 | 6360 | 14C/AMS 14C | 15 |
| 878 | Qagnax Cave, St. Paul Island | N/A | -171.65 | 57.17 | 35 | 6670 | 6430 | 14C/AMS 14C | 15 |
| 878 | Qagnax Cave, St. Paul Island | N/A | -171.65 | 57.17 | 40 | 6700 | 6460 | 14C/AMS 14C | 15 |
| 881 | Rausu 1 | N/A | 145.32 | 43.97 | 120 | 30460 | 29540 | 14C/AMS 14C | 15 |
| 882 | Rausu 2 | N/A | 145.52 | 43.93 | 760 | 44590 | 42070 | 14C/AMS 14C | 15 |
| 883 | Razvodnaya | N/A | 104.68 | 52 | 295 | 35830 | 34310 | 14C/AMS 14C | 15 |
| 894 | Rychkovo | N/A | 62.33 | 59.27 | 320 | 22260 | 20260 | 14C/AMS 14C | 15 |
| 896 | Sabettayakha River | N/A | 71.33 | 71.15 | 130 | 12760 | 11880 | 14C/AMS 14C | 15 |
| 897 | Sabler Cape | N/A | 100.48 | 74.53 | 180 | 14260 | 13380 | 14C/AMS 14C | 15 |
| 897 | Sabler Cape | N/A | 100.48 | 74.53 | 130 | 17220 | 15340 | 14C/AMS 14C | 15 |
| 897 | Sabler Cape | N/A | 100.48 | 74.53 | 70 | 17260 | 17020 | 14C/AMS 14C | 15 |
| 897 | Sabler Cape | N/A | 100.48 | 74.53 | 50 | 18870 | 18550 | 14C/AMS 14C | 15 |
| 897 | Sabler Cape | N/A | 100.48 | 74.53 | 400 | 29730 | 27770 | 14C/AMS 14C | 15 |
| 897 | Sabler Cape | N/A | 100.48 | 74.53 | 260 | 30540 | 29340 | 14C/AMS 14C | 15 |
| 897 | Sabler Cape | N/A | 100.48 | 74.53 | 450 | 35800 | 33480 | 14C/AMS 14C | 15 |
| 897 | Sabler Cape | N/A | 100.48 | 74.53 | 270 | 39420 | 36420 | 14C/AMS 14C | 15 |
| 897 | Sabler Cape | N/A | 100.48 | 74.53 | 700 | 45600 | 42880 | 14C/AMS 14C | 15 |
| 899 | Samarga River | N/A | 138.62 | 47.33 | 1700 | 45300 | 41140 | 14C/AMS 14C | 15 |
| 900 | San Shan | N/A | 126.33 | 45.5 | 600 | 26260 | 23140 | 14C/AMS 14C | 15 |
| 902 | Sanga-Yuriakh | N/A | 125 | 70 | 3000 | 41320 | 28200 | 14C/AMS 14C | 15 |
| 902 | Sanga-Yuriakh | N/A | 125 | 70 | 2000 | 41560 | 32000 | 14C/AMS 14C | 15 |
| 902 | Sanga-Yuriakh | N/A | 125 | 70 | 2500 | 43250 | 32570 | 14C/AMS 14C | 15 |
| 902 | Sanga-Yuriakh | N/A | 125 | 70 | 3500 | 54880 | 41480 | 14C/AMS 14C | 15 |
| 905 | Semieriskyai River | N/A | 110.25 | 73.37 | 500 | 42810 | 41610 | 14C/AMS 14C | 15 |
| 909 | Severnaya River | N/A | 112 | 75.5 | 120 | 14770 | 13850 | 14C/AMS 14C | 15 |
| 909 | Severnaya River | N/A | 112 | 75.5 | 120 | 15180 | 14180 | 14C/AMS 14C | 15 |
| 909 | Severnaya River | N/A | 112 | 75.5 | 400 | 36510 | 34950 | 14C/AMS 14C | 15 |
| 909 | Severnaya River | N/A | 112 | 75.5 | 500 | 39000 | 35440 | 14C/AMS 14C | 15 |
| 910 | Severobaikalsk | N/A | 109.33 | 55.63 | 535 | 43630 | 41910 | 14C/AMS 14C | 15 |
| 911 | Shaitan Lake | N/A | 98.17 | 72.8 | 2000 | 48820 | 41900 | 14C/AMS 14C | 15 |
| 912 | Shaitanskaya Cave | N/A | 60.22 | 60.46 | 670 | 28960 | 25440 | 14C/AMS 14C | 15 |
| 913 | Shalaurov Cape | N/A | 143.58 | 73.22 | 1000 | 38290 | 34170 | 14C/AMS 14C | 15 |
| 913 | Shalaurov Cape | N/A | 143.58 | 73.22 | 2000 | 42870 | 34230 | 14C/AMS 14C | 15 |
| 914 | Shandrin River | N/A | 150.3 | 71.2 | 420 | 42490 | 41450 | 14C/AMS 14C | 15 |
| 914 | Shandrin River | N/A | 150.3 | 71.2 | 880 | 45610 | 42610 | 14C/AMS 14C | 15 |
| 914 | Shandrin River | N/A | 150.3 | 71.2 | 1290 | 47770 | 43090 | 14C/AMS 14C | 15 |
| 915 | Shchuchya River | N/A | 68 | 67.1 | 80 | 15150 | 14510 | 14C/AMS 14C | 15 |
| 915 | Shchuchya River | N/A | 68 | 67.1 | 480 | 39440 | 35720 | 14C/AMS 14C | 15 |
| 915 | Shchuchya River | N/A | 68 | 67.1 | 300 | 41860 | 38940 | 14C/AMS 14C | 15 |
| 917 | Shestakovo | N/A | 88 | 55.64 | 175 | 22290 | 21170 | 14C/AMS 14C | 15 |
| 917 | Shestakovo | N/A | 88 | 55.64 | 310 | 23770 | 22250 | 14C/AMS 14C | 15 |
| 917 | Shestakovo | N/A | 88 | 55.64 | 180 | 25040 | 23960 | 14C/AMS 14C | 15 |
| 917 | Shestakovo | N/A | 88 | 55.64 | 560 | 26400 | 23480 | 14C/AMS 14C | 15 |
| 917 | Shestakovo | N/A | 88 | 55.64 | 420 | 26700 | 24420 | 14C/AMS 14C | 15 |
| 917 | Shestakovo | N/A | 88 | 55.64 | 185 | 27630 | 25950 | 14C/AMS 14C | 15 |
| 917 | Shestakovo | N/A | 88 | 55.64 | 125 | 27660 | 25980 | 14C/AMS 14C | 15 |
| 917 | Shestakovo | N/A | 88 | 55.64 | 200 | 27900 | 25980 | 14C/AMS 14C | 15 |
| 917 | Shestakovo | N/A | 88 | 55.64 | 280 | 28140 | 25940 | 14C/AMS 14C | 15 |
| 917 | Shestakovo | N/A | 88 | 55.64 | 160 | 28030 | 27150 | 14C/AMS 14C | 15 |
| 917 | Shestakovo | N/A | 88 | 55.64 | 170 | 28290 | 27370 | 14C/AMS 14C | 15 |
| 917 | Shestakovo | N/A | 88 | 55.64 | 110 | 28460 | 27780 | 14C/AMS 14C | 15 |
| 920 | Shikaevka 2 | N/A | 65.92 | 56 | 95 | 22010 | 21490 | 14C/AMS 14C | 15 |
| 921 | Shirokostan Peninsula | N/A | 139.73 | 72.36 | 70 | 18670 | 18030 | 14C/AMS 14C | 15 |
| 921 | Shirokostan Peninsula | N/A | 139.73 | 72.36 | 150 | 24380 | 23580 | 14C/AMS 14C | 15 |
| 922 | Shlenka | N/A | 92.05 | 55.2 | 2000 | 27150 | 17710 | 14C/AMS 14C | 15 |
| 922 | Shlenka | N/A | 92.05 | 55.2 | 100 | 24310 | 23670 | 14C/AMS 14C | 15 |
| 923 | Shokalsky Island | N/A | 74.4 | 73 | 160 | 17360 | 16680 | 14C/AMS 14C | 15 |
| 923 | Shokalsky Island | N/A | 74.4 | 73 | 170 | 17380 | 16740 | 14C/AMS 14C | 15 |
| 924 | Shrenk River | N/A | 98 | 75.15 | 600 | 35670 | 31430 | 14C/AMS 14C | 15 |
| 925 | Shuryshkarsky Sor | N/A | 65.27 | 65.95 | 230 | 21740 | 20060 | 14C/AMS 14C | 15 |
| 926 | Sobo Sise Islahd | N/A | 128.42 | 72.47 | 50 | 17790 | 17390 | 14C/AMS 14C | 15 |
| 926 | Sobo Sise Islahd | N/A | 128.42 | 72.47 | 220 | 23660 | 22340 | 14C/AMS 14C | 15 |
| 926 | Sobo Sise Islahd | N/A | 128.42 | 72.47 | 650 | 30720 | 27880 | 14C/AMS 14C | 15 |
| 927 | Sokhonto | N/A | 69.88 | 69.18 | 1000 | 37000 | 31880 | 14C/AMS 14C | 15 |
| 930 | Sopochnaya | N/A | 106.75 | 72.83 | 40 | 17210 | 17010 | 14C/AMS 14C | 15 |
| 930 | Sopochnaya | N/A | 106.75 | 72.83 | 130 | 31030 | 30310 | 14C/AMS 14C | 15 |
| 935 | Srednekan River | N/A | 150.3 | 62.45 | 300 | 34520 | 31520 | 14C/AMS 14C | 15 |
| 936 | Stary Vitim Channel | N/A | 113.92 | 57.82 | 100 | 24390 | 23830 | 14C/AMS 14C | 15 |
| 939 | Sualema River | N/A | 113.16 | 73.14 | 300 | 37890 | 36090 | 14C/AMS 14C | 15 |
| 948 | Svyatoy Nos Cape | N/A | 141.08 | 72.85 | 130 | 24110 | 23390 | 14C/AMS 14C | 15 |
| 948 | Svyatoy Nos Cape | N/A | 141.08 | 72.85 | 700 | 29070 | 25430 | 14C/AMS 14C | 15 |
| 948 | Svyatoy Nos Cape | N/A | 141.08 | 72.85 | 600 | 29180 | 26140 | 14C/AMS 14C | 15 |
| 948 | Svyatoy Nos Cape | N/A | 141.08 | 72.85 | 300 | 30820 | 29460 | 14C/AMS 14C | 15 |
| 948 | Svyatoy Nos Cape | N/A | 141.08 | 72.85 | 600 | 33240 | 30360 | 14C/AMS 14C | 15 |
| 948 | Svyatoy Nos Cape | N/A | 141.08 | 72.85 | 300 | 35330 | 33250 | 14C/AMS 14C | 15 |
| 948 | Svyatoy Nos Cape | N/A | 141.08 | 72.85 | 400 | 41880 | 38800 | 14C/AMS 14C | 15 |
| 948 | Svyatoy Nos Cape | N/A | 141.08 | 72.85 | 800 | 42190 | 38270 | 14C/AMS 14C | 15 |
| 948 | Svyatoy Nos Cape | N/A | 141.08 | 72.85 | 900 | 45780 | 42660 | 14C/AMS 14C | 15 |
| 948 | Svyatoy Nos Cape | N/A | 141.08 | 72.85 | 1500 | 47100 | 42180 | 14C/AMS 14C | 15 |
| 948 | Svyatoy Nos Cape | N/A | 141.08 | 72.85 | 900 | 48480 | 44120 | 14C/AMS 14C | 15 |
| 948 | Svyatoy Nos Cape | N/A | 141.08 | 72.85 | 1400 | 56430 | 47990 | 14C/AMS 14C | 15 |
| 949 | Synya River | N/A | 64.43 | 65.04 | 155 | 17810 | 17210 | 14C/AMS 14C | 15 |
| 953 | Taimyr Lake | N/A | 100.33 | 74.32 | 180 | 13410 | 12690 | 14C/AMS 14C | 15 |
| 953 | Taimyr Lake | N/A | 100.33 | 74.32 | 170 | 34080 | 31640 | 14C/AMS 14C | 15 |
| 954 | Taimyr Peninsula | N/A | 103 | 74 | 70 | 25150 | 24310 | 14C/AMS 14C | 15 |
| 954 | Taimyr Peninsula | N/A | 103 | 74 | 210 | 34070 | 31550 | 14C/AMS 14C | 15 |
| 954 | Taimyr Peninsula | N/A | 103 | 74 | 280 | 39440 | 36640 | 14C/AMS 14C | 15 |
| 955 | Talalakh Lake | N/A | 106.83 | 73.07 | 280 | 35940 | 34660 | 14C/AMS 14C | 15 |
| 955 | Talalakh Lake | N/A | 106.83 | 73.07 | 290 | 36710 | 35350 | 14C/AMS 14C | 15 |
| 956 | Tanon River | N/A | 150.55 | 59.45 | 200 | 27040 | 25000 | 14C/AMS 14C | 15 |
| 957 | Tarachikha | N/A | 91.1 | 55.15 | 320 | 23650 | 21930 | 14C/AMS 14C | 15 |
| 958 | Taradanovo | N/A | 81.83 | 53.78 | 390 | 33410 | 30810 | 14C/AMS 14C | 15 |
| 959 | Tavda River | N/A | 64 | 59 | 410 | 28580 | 26620 | 14C/AMS 14C | 15 |
| 959 | Tavda River | N/A | 64 | 59 | 800 | 47030 | 43790 | 14C/AMS 14C | 15 |
| 962 | Teguldet | N/A | 88.08 | 57.33 | 90 | 23990 | 23390 | 14C/AMS 14C | 15 |
| 962 | Teguldet | N/A | 88.08 | 57.33 | 180 | 24430 | 23550 | 14C/AMS 14C | 15 |
| 963 | Terektyakh River | N/A | 147.08 | 68.58 | 630 | 42890 | 39250 | 14C/AMS 14C | 15 |
| 963 | Terektyakh River | N/A | 147.08 | 68.58 | 1900 | 51880 | 43920 | 14C/AMS 14C | 15 |
| 964 | Terpyi-Tumus Peninsula | N/A | 118.5 | 73.55 | 340 | 34620 | 31420 | 14C/AMS 14C | 15 |
| 964 | Terpyi-Tumus Peninsula | N/A | 118.5 | 73.55 | 300 | 35960 | 34640 | 14C/AMS 14C | 15 |
| 965 | Tesa River | N/A | 112 | 57.3 | 200 | 21800 | 20280 | 14C/AMS 14C | 15 |
| 965 | Tesa River | N/A | 112 | 57.3 | 420 | 28320 | 25760 | 14C/AMS 14C | 15 |
| 965 | Tesa River | N/A | 112 | 57.3 | 195 | 28120 | 27120 | 14C/AMS 14C | 15 |
| 965 | Tesa River | N/A | 112 | 57.3 | 600 | 35540 | 31260 | 14C/AMS 14C | 15 |
| 975 | Trautfetter River | N/A | 101.8 | 75.63 | 110 | 29610 | 28490 | 14C/AMS 14C | 15 |
| 975 | Trautfetter River | N/A | 101.8 | 75.63 | 1300 | 44950 | 41670 | 14C/AMS 14C | 15 |
| 975 | Trautfetter River | N/A | 101.8 | 75.63 | 910 | 44990 | 42270 | 14C/AMS 14C | 15 |
| 976 | Troitskaya | N/A | 61.4 | 54.1 | 300 | 20280 | 18840 | 14C/AMS 14C | 15 |
| 987 | Tyung | N/A | 116 | 67.35 | 200 | 28420 | 27420 | 14C/AMS 14C | 15 |
| 987 | Tyung | N/A | 116 | 67.35 | 500 | 35960 | 33440 | 14C/AMS 14C | 15 |
| 989 | Uelen | N/A | -170 | 66.3 | 70 | 17860 | 17420 | 14C/AMS 14C | 15 |
| 990 | Ulakhan-Taala | N/A | 141.6 | 72.75 | 1200 | 46670 | 42550 | 14C/AMS 14C | 15 |
| 991 | Ulakhan-Yuriakh River | N/A | 104 | 72.12 | 50 | 18200 | 18000 | 14C/AMS 14C | 15 |
| 992 | Uralovka | N/A | 128.11 | 52.45 | 200 | 30530 | 29450 | 14C/AMS 14C | 15 |
| 993 | Urasalakh River | N/A | 116.16 | 73.67 | 600 | 31270 | 28990 | 14C/AMS 14C | 15 |
| 994 | Urtuiskoe | N/A | 118 | 50.11 | 200 | 34530 | 32010 | 14C/AMS 14C | 15 |
| 995 | Ushlep 6 | 3 | 86.68 | 52.85 | 390 | 21790 | 19310 | 14C/AMS 14C | 15 |
| 997 | Ushlep 6 | N/A | 86.68 | 52.85 | 1100 | 45350 | 42230 | 14C/AMS 14C | 15 |
| 998 | Uspenka | N/A | 70.5 | 54.87 | 410 | 29640 | 27560 | 14C/AMS 14C | 15 |
| 1000 | Vacha River | N/A | 115.31 | 58.24 | 420 | 44530 | 42450 | 14C/AMS 14C | 15 |
| 1003 | Verkhnaya Taimyra River, Hook Woolly mammoth | N/A | 99.59 | 74.15 | 90 | 24990 | 24070 | 14C/AMS 14C | 15 |
| 1003 | Verkhnaya Taimyra River, Hook Woolly mammoth | N/A | 99.59 | 74.15 | 70 | 25010 | 24210 | 14C/AMS 14C | 15 |
| 1007 | Voenny Gospital | N/A | 104.32 | 52.3 | 450 | 28700 | 26540 | 14C/AMS 14C | 15 |
| 1010 | Volchya Griva (2) | N/A | 80.2 | 54.5 | 120 | 13250 | 12730 | 14C/AMS 14C | 15 |
| 1010 | Volchya Griva (2) | N/A | 80.2 | 54.5 | 150 | 15310 | 14270 | 14C/AMS 14C | 15 |
| 1010 | Volchya Griva (2) | N/A | 80.2 | 54.5 | 230 | 17590 | 16030 | 14C/AMS 14C | 15 |
| 1010 | Volchya Griva (2) | N/A | 80.2 | 54.5 | 150 | 17820 | 17220 | 14C/AMS 14C | 15 |
| 1010 | Volchya Griva (2) | N/A | 80.2 | 54.5 | 285 | 18200 | 17120 | 14C/AMS 14C | 15 |
| 1010 | Volchya Griva (2) | N/A | 80.2 | 54.5 | 260 | 18230 | 17190 | 14C/AMS 14C | 15 |
| 1010 | Volchya Griva (2) | N/A | 80.2 | 54.5 | 150 | 18700 | 17620 | 14C/AMS 14C | 15 |
| 1010 | Volchya Griva (2) | N/A | 80.2 | 54.5 | 210 | 20570 | 19610 | 14C/AMS 14C | 15 |
| 1010 | Volchya Griva (2) | N/A | 80.2 | 54.5 | 100 | 21850 | 20690 | 14C/AMS 14C | 15 |
| 1036 | Wrangel Island | N/A | -179 | 71 | 60 | 4210 | 3850 | 14C/AMS 14C | 15 |
| 1036 | Wrangel Island | N/A | -179 | 71 | 40 | 4220 | 3940 | 14C/AMS 14C | 15 |
| 1036 | Wrangel Island | N/A | -179 | 71 | 30 | 4460 | 4260 | 14C/AMS 14C | 15 |
| 1036 | Wrangel Island | N/A | -179 | 71 | 50 | 4620 | 4380 | 14C/AMS 14C | 15 |
| 1036 | Wrangel Island | N/A | -179 | 71 | 30 | 4610 | 4410 | 14C/AMS 14C | 15 |
| 1036 | Wrangel Island | N/A | -179 | 71 | 70 | 5270 | 4750 | 14C/AMS 14C | 15 |
| 1036 | Wrangel Island | N/A | -179 | 71 | 40 | 5120 | 4840 | 14C/AMS 14C | 15 |
| 1036 | Wrangel Island | N/A | -179 | 71 | 50 | 5330 | 4770 | 14C/AMS 14C | 15 |
| 1036 | Wrangel Island | N/A | -179 | 71 | 40 | 5670 | 5270 | 14C/AMS 14C | 15 |
| 1036 | Wrangel Island | N/A | -179 | 71 | 40 | 5720 | 5560 | 14C/AMS 14C | 15 |
| 1036 | Wrangel Island | N/A | -179 | 71 | 40 | 5980 | 5700 | 14C/AMS 14C | 15 |
| 1036 | Wrangel Island | N/A | -179 | 71 | 30 | 6020 | 5900 | 14C/AMS 14C | 15 |
| 1036 | Wrangel Island | N/A | -179 | 71 | 40 | 6200 | 5880 | 14C/AMS 14C | 15 |
| 1036 | Wrangel Island | N/A | -179 | 71 | 90 | 6320 | 5880 | 14C/AMS 14C | 15 |
| 1036 | Wrangel Island | N/A | -179 | 71 | 50 | 6380 | 6180 | 14C/AMS 14C | 15 |
| 1036 | Wrangel Island | N/A | -179 | 71 | 50 | 7330 | 7010 | 14C/AMS 14C | 15 |
| 1036 | Wrangel Island | N/A | -179 | 71 | 60 | 7450 | 7170 | 14C/AMS 14C | 15 |
| 1036 | Wrangel Island | N/A | -179 | 71 | 50 | 7610 | 7410 | 14C/AMS 14C | 15 |
| 1036 | Wrangel Island | N/A | -179 | 71 | 30 | 7680 | 7560 | 14C/AMS 14C | 15 |
| 1036 | Wrangel Island | N/A | -179 | 71 | 50 | 7700 | 7540 | 14C/AMS 14C | 15 |
| 1036 | Wrangel Island | N/A | -179 | 71 | 50 | 7830 | 7630 | 14C/AMS 14C | 15 |
| 1036 | Wrangel Island | N/A | -179 | 71 | 60 | 7990 | 7750 | 14C/AMS 14C | 15 |
| 1036 | Wrangel Island | N/A | -179 | 71 | 60 | 8220 | 7940 | 14C/AMS 14C | 15 |
| 1036 | Wrangel Island | N/A | -179 | 71 | 95 | 8330 | 7930 | 14C/AMS 14C | 15 |
| 1036 | Wrangel Island | N/A | -179 | 71 | 50 | 8360 | 8000 | 14C/AMS 14C | 15 |
| 1036 | Wrangel Island | N/A | -179 | 71 | 40 | 8600 | 8400 | 14C/AMS 14C | 15 |
| 1036 | Wrangel Island | N/A | -179 | 71 | 110 | 14360 | 13600 | 14C/AMS 14C | 15 |
| 1036 | Wrangel Island | N/A | -179 | 71 | 85 | 14230 | 13670 | 14C/AMS 14C | 15 |
| 1036 | Wrangel Island | N/A | -179 | 71 | 50 | 15410 | 15010 | 14C/AMS 14C | 15 |
| 1036 | Wrangel Island | N/A | -179 | 71 | 80 | 16590 | 15110 | 14C/AMS 14C | 15 |
| 1036 | Wrangel Island | N/A | -179 | 71 | 100 | 18930 | 18530 | 14C/AMS 14C | 15 |
| 1036 | Wrangel Island | N/A | -179 | 71 | 110 | 24240 | 23560 | 14C/AMS 14C | 15 |
| 1036 | Wrangel Island | N/A | -179 | 71 | 200 | 27890 | 25970 | 14C/AMS 14C | 15 |
| 1036 | Wrangel Island | N/A | -179 | 71 | 300 | 28000 | 25880 | 14C/AMS 14C | 15 |
| 1037 | Xiaonanshan | N/A | 134.03 | 46.78 | 410 | 17430 | 13990 | 14C/AMS 14C | 15 |
| 1038 | Xietian | N/A | 127.6 | 45 | 3500 | 49260 | 36740 | 14C/AMS 14C | 15 |
| 1038 | Xietian | N/A | 127.6 | 45 | 3000 | 48810 | 39370 | 14C/AMS 14C | 15 |
| 1038 | Xietian | N/A | 127.6 | 45 | 3500 | 50560 | 38880 | 14C/AMS 14C | 15 |
| 1040 | Yamal Peninsula | N/A | 66.5 | 71 | 350 | 34440 | 31320 | 14C/AMS 14C | 15 |
| 1040 | Yamal Peninsula | N/A | 66.5 | 71 | 300 | 35440 | 33560 | 14C/AMS 14C | 15 |
| 1041 | Yambuto Lake | N/A | 79.2 | 71.02 | 500 | 33270 | 30590 | 14C/AMS 14C | 15 |
| 1042 | Yana RHS | N/A | 135.42 | 70.63 | 600 | 33820 | 30580 | 14C/AMS 14C | 15 |
| 1043 | Yana River | N/A | 134.47 | 68.12 | 195 | 28120 | 27120 | 14C/AMS 14C | 15 |
| 1045 | Yekaryauyakha River | N/A | 80.66 | 71.55 | 1000 | 39580 | 34460 | 14C/AMS 14C | 15 |
| 1046 | Yubari | N/A | 141.97 | 43.08 | 90 | 19860 | 19300 | 14C/AMS 14C | 15 |
| 1047 | Yukagir Woolly mammoth | N/A | 139.92 | 71.75 | 110 | 22300 | 21500 | 14C/AMS 14C | 15 |
| 1049 | Yuni | N/A | 141.78 | 43 | 250 | 42930 | 41850 | 14C/AMS 14C | 15 |
| 1049 | Yuni | N/A | 141.78 | 43 | 480 | 50270 | 46110 | 14C/AMS 14C | 15 |
| 1050 | Yunkyur-Seene River | N/A | 117 | 73.63 | 300 | 42030 | 41190 | 14C/AMS 14C | 15 |
| 1050 | Yunkyur-Seene River | N/A | 117 | 73.63 | 1000 | 44980 | 42140 | 14C/AMS 14C | 15 |
| 1052 | Yuribei River | N/A | 71 | 68.92 | 100 | 11390 | 10710 | 14C/AMS 14C | 15 |
| 1052 | Yuribei River | N/A | 71 | 68.92 | 70 | 11830 | 11190 | 14C/AMS 14C | 15 |
| 1052 | Yuribei River | N/A | 71 | 68.92 | 120 | 12790 | 11950 | 14C/AMS 14C | 15 |
| 1052 | Yuribei River | N/A | 71 | 68.92 | 170 | 17520 | 17120 | 14C/AMS 14C | 15 |
| 1052 | Yuribei River | N/A | 71 | 68.92 | 300 | 30960 | 29800 | 14C/AMS 14C | 15 |
| 1053 | Zelenaya Seyakha River | N/A | 69 | 70.15 | 80 | 17900 | 17420 | 14C/AMS 14C | 15 |
| 1055 | Zhalainuoer | N/A | 117.58 | 49.35 | 1300 | 34730 | 28850 | 14C/AMS 14C | 15 |
| 1055 | Zhalainuoer | N/A | 117.58 | 49.35 | 1700 | 42090 | 34050 | 14C/AMS 14C | 15 |
| 1056 | Zhemchuzhnaya Cave | N/A | 57.78 | 54.9 | 320 | 22550 | 20390 | 14C/AMS 14C | 15 |
| 1058 | Zhokhov Island | N/A | 152.75 | 76.1 | 2600 | 46020 | 36900 | 14C/AMS 14C | 15 |
| 1059 | Zhoujiayoufang | N/A | 126.53 | 44.76 | 1220 | 36550 | 30670 | 14C/AMS 14C | 15 |
| 751 | Mamont River | N/A | 96 | 75.15 | 250 | 13830 | 12870 | 14C/AMS 14C | 15; 21 |
| 1173 | Willendorf II | 5 | 15.399 | 48.323 | 2900 | 34020 | 21340 | 14C/AMS 14C | 16 |
| 1173 | Willendorf II | 5 | 15.399 | 48.323 | 2600 | 34180 | 22780 | 14C/AMS 14C | 16 |
| 1173 | Willendorf II | 5 | 15.399 | 48.323 | 290 | 32270 | 30990 | 14C/AMS 14C | 16 |
| 1173 | Willendorf II | 5 | 15.399 | 48.323 | 850 | 37290 | 33850 | 14C/AMS 14C | 16 |
| 1172 | Jarošov | N/A | 17.5 | 49 | 600 | 31090 | 28610 | 14C/AMS 14C | 16 |
| 1172 | Jarošov | N/A | 17.5 | 49 | 600 | 31370 | 29250 | 14C/AMS 14C | 16 |
| 1172 | Jarošov | N/A | 17.5 | 49 | 245 | 31100 | 30220 | 14C/AMS 14C | 16 |
| 1172 | Jarošov | N/A | 17.5 | 49 | 235 | 30630 | 29470 | 14C/AMS 14C | 16 |
| 1172 | Jarošov | N/A | 17.5 | 49 | 375 | 31360 | 30360 | 14C/AMS 14C | 16 |
| 1172 | Jarošov | N/A | 17.5 | 49 | 180 | 31260 | 30740 | 14C/AMS 14C | 16 |
| 1172 | Jarošov | N/A | 17.5 | 49 | 200 | 31480 | 31080 | 14C/AMS 14C | 16 |
| 1177 | Willendorf II | 9 | 15.399 | 48.323 | 120 | 28370 | 27610 | 14C/AMS 14C | 16 |
| 1177 | Willendorf II | 9 | 15.399 | 48.323 | 270 | 29540 | 28060 | 14C/AMS 14C | 16 |
| 1177 | Willendorf II | 9 | 15.399 | 48.323 | 290 | 30260 | 28380 | 14C/AMS 14C | 16 |
| 1177 | Willendorf II | 9 | 15.399 | 48.323 | 150 | 30430 | 29390 | 14C/AMS 14C | 16 |
| 1178 | Bodrogkeretúr | N/A | 21.38 | 48.12 | 3000 | 40080 | 27560 | 14C/AMS 14C | 16 |
| 1182 | Petřkovice | N/A | 18.25 | 49.87 | 270 | 25670 | 24030 | 14C/AMS 14C | 16 |
| 1182 | Petřkovice | N/A | 18.25 | 49.87 | 160 | 28550 | 27750 | 14C/AMS 14C | 16 |
| 1183 | Kulna Cave | 6b | 16.75 | 49.41 | 140 | 27270 | 25510 | 14C/AMS 14C | 16 |
| 1183 | Kulna Cave | 6b | 16.75 | 49.41 | 150 | 26880 | 25240 | 14C/AMS 14C | 16 |
| 1183 | Kulna Cave | 6b | 16.75 | 49.41 | 170 | 28290 | 27370 | 14C/AMS 14C | 16 |
| 1183 | Kulna Cave | 6b | 16.75 | 49.41 | 140 | 26060 | 24860 | 14C/AMS 14C | 16 |
| 1184 | Grubgraben | 1-5 | 16.7 | 46.6 | 300 | 23140 | 21620 | 14C/AMS 14C | 16 |
| 1184 | Grubgraben | 1-5 | 16.7 | 46.6 | 130 | 22530 | 21650 | 14C/AMS 14C | 16 |
| 1184 | Grubgraben | 1-5 | 16.7 | 46.6 | 330 | 22730 | 21370 | 14C/AMS 14C | 16 |
| 1184 | Grubgraben | 1-5 | 16.7 | 46.6 | 140 | 22920 | 22320 | 14C/AMS 14C | 16 |
| 1184 | Grubgraben | 1-5 | 16.7 | 46.6 | 180 | 23150 | 22270 | 14C/AMS 14C | 16 |
| 1184 | Grubgraben | 1-5 | 16.7 | 46.6 | 290 | 23620 | 22060 | 14C/AMS 14C | 16 |
| 1184 | Grubgraben | 1-5 | 16.7 | 46.6 | 90 | 23630 | 22670 | 14C/AMS 14C | 16 |
| 1151 | Bohunice | N/A | 16.61 | 49.178 | 1550 | 49610 | 43410 | 14C/AMS 14C | 16 |
| 1151 | Bohunice | N/A | 16.61 | 49.178 | 1300 | 47440 | 42760 | 14C/AMS 14C | 16 |
| 1151 | Bohunice | N/A | 16.61 | 49.178 | 1100 | 45690 | 42370 | 14C/AMS 14C | 16 |
| 1151 | Bohunice | N/A | 16.61 | 49.178 | 1100 | 43270 | 38710 | 14C/AMS 14C | 16 |
| 1152 | Bohunice | IIIa | 16.61 | 49.178 | 2700 | 50110 | 40990 | 14C/AMS 14C | 16 |
| 1152 | Bohunice | IIIa | 16.61 | 49.178 | 1100 | 44480 | 41560 | 14C/AMS 14C | 16 |
| 1152 | Bohunice | IIIa | 16.61 | 49.178 | 1550 | 45070 | 41270 | 14C/AMS 14C | 16 |
| 1187 | Stránská skála IV | N/A | 16.68 | 49.2 | 120 | 22410 | 21530 | 14C/AMS 14C | 16 |
| 1188 | Velké Pavlovice | N/A | 16.82 | 48.9 | 230 | 18240 | 17280 | 14C/AMS 14C | 16 |
| 622 | Kent’s Cavern, Devon | N/A | -3.5 | 50.47 | 130 | 13840 | 13240 | 14C/AMS 14C | 8 |
| 477 | Coygan Cave | N/A | -4.4962 | 51.761 | 320 | 30470 | 28710 | 14C/AMS 14C | 7 |
| 537 | Gough's Cave | N/A | -2.78 | 51.28 | 120 | 15200 | 14320 | 14C/AMS 14C | 7 |
| 537 | Gough's Cave | N/A | -2.78 | 51.28 | 140 | 16730 | 14890 | 14C/AMS 14C | 7 |
| 622 | Kent’s Cavern, Devon | N/A | -3.5 | 50.47 | 110 | 14730 | 13850 | 14C/AMS 14C | 12 |
| 622 | Kent’s Cavern, Devon | N/A | -3.5 | 50.47 | 260 | 28470 | 27310 | 14C/AMS 14C | 12 |
| 622 | Kent’s Cavern, Devon | N/A | -3.5 | 50.47 | 400 | 33770 | 30930 | 14C/AMS 14C | 12 |
| 622 | Kent’s Cavern, Devon | N/A | -3.5 | 50.47 | 500 | 34070 | 30870 | 14C/AMS 14C | 12 |
| 622 | Kent’s Cavern, Devon | N/A | -3.5 | 50.47 | 440 | 34250 | 31050 | 14C/AMS 14C | 12 |
| 622 | Kent’s Cavern, Devon | N/A | -3.5 | 50.47 | 440 | 35450 | 32170 | 14C/AMS 14C | 12 |
| 820 | Oblazowa Cave, Poland | N/A | 20.2 | 49.25 | 260 | 22570 | 21170 | 14C/AMS 14C | 12 |
| 622 | Kent’s Cavern, Devon | N/A | -3.5 | 50.47 | 800 | 42190 | 38270 | 14C/AMS 14C | 12 |
| 622 | Kent’s Cavern, Devon | N/A | -3.5 | 50.47 | 820 | 42240 | 38360 | 14C/AMS 14C | 12 |
| 450 | Castlepook Cave, Co. Cork | N/A | -8.65 | 52.28 | 840 | 42410 | 36930 | 14C/AMS 14C | 13 |
| 1005 | Villefranche-s-Saone, France | N/A | 4.72 | 45.98 | 240 | 26270 | 24790 | 14C/AMS 14C | 14 |
| 1017 | Wildscheuer Cave, Germany | N/A | 8.137 | 50.421 | 360 | 25310 | 23670 | 14C/AMS 14C | 10 |
| 1017 | Wildscheuer Cave, Germany | N/A | 8.137 | 50.421 | 420 | 34690 | 31250 | 14C/AMS 14C | 10 |
| 1017 | Wildscheuer Cave, Germany | N/A | 8.137 | 50.421 | 550 | 36410 | 34010 | 14C/AMS 14C | 10 |
| 1017 | Wildscheuer Cave, Germany | N/A | 8.137 | 50.421 | 1100 | 37620 | 32460 | 14C/AMS 14C | 10 |
| 1017 | Wildscheuer Cave, Germany | N/A | 8.137 | 50.421 | 600 | 37240 | 35080 | 14C/AMS 14C | 10 |
| 1017 | Wildscheuer Cave, Germany | N/A | 8.137 | 50.421 | 700 | 39820 | 36140 | 14C/AMS 14C | 10 |
| 1017 | Wildscheuer Cave, Germany | N/A | 8.137 | 50.421 | 750 | 41770 | 36130 | 14C/AMS 14C | 10 |
| 1017 | Wildscheuer Cave, Germany | N/A | 8.137 | 50.421 | 1200 | 42680 | 36240 | 14C/AMS 14C | 10 |
| 622 | Kent’s Cavern, Devon | N/A | -3.5 | 50.47 | 100 | 14680 | 13880 | 14C/AMS 14C | 3 |
| 414 | Berdyzh, USSR | N/A | 30.95 | 52.84 | 250 | 18840 | 17920 | 14C/AMS 14C | 5 |
| 646 | Kirillovskaya, USSR | N/A | 102.9 | 53.5 | 350 | 23840 | 22200 | 14C/AMS 14C | 5 |
| 759 | Mezhirich, USSR | N/A | 34.48 | 50.69 | 250 | 18230 | 17230 | 14C/AMS 14C | 5 |
| 760 | Mezin, USSR | N/A | 33.08 | 51.81 | 200 | 18760 | 18000 | 14C/AMS 14C | 5 |
| 472 | Condover Shropshire | N/A | -2.76 | 52.64 | 160 | 15810 | 14450 | 14C/AMS 14C | 11 |
| 622 | Kent’s Cavern, Devon | N/A | -3.5 | 50.47 | 800 | 37770 | 34770 | 14C/AMS 14C | 11 |
| 814 | Novgorod-Severskii, USSR | N/A | 33.27 | 52 | 350 | 24540 | 22620 | 14C/AMS 14C | 11 |
| 879 | Radomyshl', USSR | N/A | 29.24 | 50.5 | 300 | 23660 | 22100 | 14C/AMS 14C | 11 |
| 409 | Bederbo-Tarida River | N/A | 102.16 | 73.06 | 200 | 26410 | 24970 | 14C/AMS 14C | 18 |
| 419 | Bikada River | N/A | 106.5 | 70.8 | 700 | 42810 | 41290 | 14C/AMS 14C | 18 |
| 493 | Duvanny Yar | N//A | 150.45 | 68.45 | 100 | 23730 | 22730 | 14C/AMS 14C | 18 |
| 508 | Engelgard Lake | N/A | 110.3 | 75.1 | 200 | 30670 | 29670 | 14C/AMS 14C | 18 |
| 516 | Faddeevsky Island | N/A | 144 | 75.26 | 400 | 35470 | 32950 | 14C/AMS 14C | 18 |
| 654 | Kolyma_River | N/A | 157.67 | 68.2 | 400 | 31830 | 30830 | 14C/AMS 14C | 18 |
| 732 | Lugovskoe | N/A | 68.57 | 61.05 | 250 | 13260 | 11980 | 14C/AMS 14C | 18 |
| 836 | Ozernaya Balya | N/A | 103.05 | 55.45 | 780 | 39600 | 35120 | 14C/AMS 14C | 18 |
| 915 | Shchuchya River | N/A | 68 | 67.1 | 170 | 31920 | 31160 | 14C/AMS 14C | 18 |
| 955 | Talalakh Lake | N/A | 106.83 | 73.07 | 610 | 42760 | 41440 | 14C/AMS 14C | 18 |
| 1036 | Wrangel Island | N/A | -179 | 71 | 60 | 18780 | 18180 | 14C/AMS 14C | 18 |
| 1036 | Wrangel Island | N/A | -179 | 71 | 340 | 35890 | 34170 | 14C/AMS 14C | 18 |
| 844 | Picken's Hole, Layer 3 | N/A | -2.8778 | 51.291 | 2600 | 43920 | 34320 | 14C/AMS 14C | 19 |
| 978 | Trou Al'Wesse | ? (old excavation) | 5.294 | 50.421 | 850 | 37470 | 34310 | 14C/AMS 14C | 19 |
| 979 | Trou Al'Wesse | Assoc. aurignacian | 5.294 | 50.421 | 660 | 39640 | 35840 | 14C/AMS 14C | 19 |
| 980 | Trou Al'Wesse | L5 / Ch17 / F507 / -758 cm | 5.294 | 50.421 | 2300 | 49080 | 41400 | 14C/AMS 14C | 19 |
| 981 | Trou Al'Wesse | L6 / Ch15 / F480 / -543 -647 cm | 5.294 | 50.421 | 1100 | 43540 | 39260 | 14C/AMS 14C | 19 |
| 659 | Korman' IV | basal layer (layer 11)+ | 27.28 | 48.27 | 2050 | 52020 | 43660 | 14C/AMS 14C | 19 |
| 562 | Gr. Tournal (or Grande Grotte de Bize) [Bize-Minervois] | ?? | 2.8818 | 43.341 | 4000 | 45210 | 29730 | 14C/AMS 14C | 19 |
| 562 | Gr. Tournal (or Grande Grotte de Bize) [Bize-Minervois] | ?? | 2.8818 | 43.341 | 8000 | 52130 | 21050 | 14C/AMS 14C | 19 |
| 418 | Betovo | N/A | 34.026 | 53.354 | 500 | 42480 | 40920 | 14C/AMS 14C | 19 |
| 132 | Roc de Combe [Nadaillac] | 6 | 1.33 | 44.07 | 1200 | 32220 | 27860 | 14C/AMS 14C | 19 |
| 132 | Roc de Combe [Nadaillac] | 6 | 1.33 | 44.07 | 500 | 33680 | 30720 | 14C/AMS 14C | 19 |
| 133 | Roc de Combe [Nadaillac] | 1a-c | 1.33 | 44.07 | 400 | 31060 | 29380 | 14C/AMS 14C | 19 |
| 133 | Roc de Combe [Nadaillac] | 1a-c | 1.33 | 44.07 | 750 | 36600 | 32920 | 14C/AMS 14C | 19 |
| 133 | Roc de Combe [Nadaillac] | 1a-c | 1.33 | 44.07 | 1000 | 39940 | 35020 | 14C/AMS 14C | 19 |
| 174 | Abri Pataud | Layer 5 | 1 | 44.93 | 1000 | 32310 | 28870 | 14C/AMS 14C | 19 |
| 174 | Abri Pataud | Layer 5 | 1 | 44.93 | 1100 | 35950 | 30550 | 14C/AMS 14C | 19 |
| 176 | Abri Pataud | Layer 3 | 1 | 44.93 | 450 | 27670 | 24710 | 14C/AMS 14C | 19 |
| 176 | Abri Pataud | Layer 3 | 1 | 44.93 | 670 | 29620 | 26300 | 14C/AMS 14C | 19 |
| 176 | Abri Pataud | Layer 3 | 1 | 44.93 | 500 | 29130 | 26890 | 14C/AMS 14C | 19 |
| 176 | Abri Pataud | Layer 3 | 1 | 44.93 | 750 | 30760 | 27560 | 14C/AMS 14C | 19 |
| 176 | Abri Pataud | Layer 3 | 1 | 44.93 | 740 | 30850 | 27770 | 14C/AMS 14C | 19 |
| 176 | Abri Pataud | Layer 3 | 1 | 44.93 | 600 | 30730 | 28050 | 14C/AMS 14C | 19 |
| 177 | Abri Pataud | Layer 2 | 1 | 44.93 | 600 | 28110 | 24790 | 14C/AMS 14C | 19 |
| 184 | Grotte du Renne, Arcy-sur-Cure | XI | 3.77 | 47.59 | 570 | 37810 | 35450 | 14C/AMS 14C | 19 |
| 185 | Roc de Combe [Nadaillac] | 5 | 1.33 | 44.07 | 700 | 35480 | 31000 | 14C/AMS 14C | 19 |
| 185 | Roc de Combe [Nadaillac] | 5 | 1.33 | 44.07 | 1000 | 39940 | 35020 | 14C/AMS 14C | 19 |
| 223 | Brean Down | N/A | -3.02 | 51.32 | 1600 | 47800 | 42240 | 14C/AMS 14C | 19 |
| 225 | Soldier's Hole | Spit 8-20 | -2.77 | 51.28 | 400 | 24030 | 22190 | 14C/AMS 14C | 19 |
| 225 | Soldier's Hole | Spit 8-20 | -2.77 | 51.28 | 550 | 31820 | 30380 | 14C/AMS 14C | 19 |
| 225 | Soldier's Hole | Spit 8-20 | -2.77 | 51.28 | 600 | 33950 | 30630 | 14C/AMS 14C | 19 |
| 225 | Soldier's Hole | Spit 8-20 | -2.77 | 51.28 | 1100 | 36810 | 31090 | 14C/AMS 14C | 19 |
| 225 | Soldier's Hole | Spit 8-20 | -2.77 | 51.28 | 450 | 36160 | 34040 | 14C/AMS 14C | 19 |
| 225 | Soldier's Hole | Spit 8-20 | -2.77 | 51.28 | 1200 | 42730 | 37890 | 14C/AMS 14C | 19 |
| 225 | Soldier's Hole | Spit 8-20 | -2.77 | 51.28 | 3500 | 52060 | 40100 | 14C/AMS 14C | 19 |
| 228 | Shandon Cave | N/A | -7.63 | 52.01 | 320 | 31290 | 30330 | 14C/AMS 14C | 19 |
| 228 | Shandon Cave | N/A | -7.63 | 52.01 | 350 | 32260 | 30900 | 14C/AMS 14C | 19 |
| 228 | Shandon Cave | N/A | -7.63 | 52.01 | 420 | 33470 | 30790 | 14C/AMS 14C | 19 |
| 228 | Shandon Cave | N/A | -7.63 | 52.01 | 400 | 33580 | 30860 | 14C/AMS 14C | 19 |
| 228 | Shandon Cave | N/A | -7.63 | 52.01 | 380 | 33650 | 30930 | 14C/AMS 14C | 19 |
| 228 | Shandon Cave | N/A | -7.63 | 52.01 | 400 | 34420 | 31220 | 14C/AMS 14C | 19 |
| 228 | Shandon Cave | N/A | -7.63 | 52.01 | 560 | 36380 | 33860 | 14C/AMS 14C | 19 |
| 228 | Shandon Cave | N/A | -7.63 | 52.01 | 540 | 37010 | 35050 | 14C/AMS 14C | 19 |
| 228 | Shandon Cave | N/A | -7.63 | 52.01 | 670 | 39670 | 35950 | 14C/AMS 14C | 19 |
| 230 | Trou Magrite | 2 | 4.97 | 50.21 | 200 | 22140 | 20580 | 14C/AMS 14C | 19 |
| 245 | Das Geissenklosterle | II | 9.77 | 48.4 | 700 | 39680 | 35760 | 14C/AMS 14C | 19 |
| 245 | Das Geissenklosterle | II | 9.77 | 48.4 | 500 | 40120 | 36640 | 14C/AMS 14C | 19 |
| 245 | Das Geissenklosterle | II | 9.77 | 48.4 | 1100 | 42030 | 35710 | 14C/AMS 14C | 19 |
| 245 | Das Geissenklosterle | II | 9.77 | 48.4 | 1100 | 42460 | 36020 | 14C/AMS 14C | 19 |
| 245 | Das Geissenklosterle | II | 9.77 | 48.4 | 1000 | 43350 | 40590 | 14C/AMS 14C | 19 |
| 251 | Wildscheuer | III | 8.14 | 50.42 | 360 | 25310 | 23670 | 14C/AMS 14C | 19 |
| 251 | Wildscheuer | III | 8.14 | 50.42 | 420 | 34690 | 31250 | 14C/AMS 14C | 19 |
| 251 | Wildscheuer | III | 8.14 | 50.42 | 550 | 36410 | 34010 | 14C/AMS 14C | 19 |
| 251 | Wildscheuer | III | 8.14 | 50.42 | 1100 | 37620 | 32460 | 14C/AMS 14C | 19 |
| 251 | Wildscheuer | III | 8.14 | 50.42 | 600 | 37240 | 35080 | 14C/AMS 14C | 19 |
| 251 | Wildscheuer | III | 8.14 | 50.42 | 700 | 39820 | 36140 | 14C/AMS 14C | 19 |
| 251 | Wildscheuer | III | 8.14 | 50.42 | 750 | 41770 | 36130 | 14C/AMS 14C | 19 |
| 251 | Wildscheuer | III | 8.14 | 50.42 | 1200 | 42680 | 36240 | 14C/AMS 14C | 19 |
| 251 | Wildscheuer | III | 8.14 | 50.42 | 900 | 42430 | 37030 | 14C/AMS 14C | 19 |
| 255 | Pavlov I | N/A | 16.69 | 48.87 | 110 | 30800 | 30200 | 14C/AMS 14C | 19 |
| 258 | Kostienki I | Cultural layer 1 | 39 | 51.29 | 200 | 24970 | 23730 | 14C/AMS 14C | 19 |
| 258 | Kostienki I | Cultural layer 1 | 39 | 51.29 | 260 | 25810 | 24050 | 14C/AMS 14C | 19 |
| 259 | Kostienki XIV [Markina Gora] | Cultural layer 2 | 39.04 | 51.39 | 420 | 35150 | 31470 | 14C/AMS 14C | 19 |
| 314 | Paviland Cave [Goat's Hole] | Red Lady | -4.24 | 51.55 | 550 | 31580 | 30220 | 14C/AMS 14C | 19 |
| 317 | Trou Magrite | 2 top | 4.97 | 50.21 | 200 | 22140 | 20580 | 14C/AMS 14C | 19 |
| 318 | Trou Magrite | 3 mid | 4.97 | 50.21 | 1690 | 48080 | 42200 | 14C/AMS 14C | 19 |
| 319 | Trou Magrite | 4a | 4.97 | 50.21 | 190 | 26470 | 25070 | 14C/AMS 14C | 19 |
| 319 | Trou Magrite | 4a | 4.97 | 50.21 | 660 | 37190 | 34870 | 14C/AMS 14C | 19 |
| 325 | Das Geissenklosterle | [I] | 9.77 | 48.4 | 380 | 30360 | 28240 | 14C/AMS 14C | 19 |
| 325 | Das Geissenklosterle | [I] | 9.77 | 48.4 | 500 | 31490 | 30250 | 14C/AMS 14C | 19 |
| 325 | Das Geissenklosterle | [I] | 9.77 | 48.4 | 550 | 33020 | 30460 | 14C/AMS 14C | 19 |
| 325 | Das Geissenklosterle | [I] | 9.77 | 48.4 | 550 | 33800 | 30680 | 14C/AMS 14C | 19 |
| 326 | Das Geissenklosterle | [IIIa] | 9.77 | 48.4 | 680 | 40530 | 36370 | 14C/AMS 14C | 19 |
| 326 | Das Geissenklosterle | [IIIa] | 9.77 | 48.4 | 640 | 41910 | 36230 | 14C/AMS 14C | 19 |
| 329 | Das Geissenklosterle | III [sq. 37] | 9.77 | 48.4 | 1800 | 45090 | 38730 | 14C/AMS 14C | 19 |
| 330 | Das Geissenklosterle | III [sq. 66] | 9.77 | 48.4 | 1050 | 44160 | 41400 | 14C/AMS 14C | 19 |
| 331 | Das Geissenklosterle | IIIa [sq. 66] | 9.77 | 48.4 | 1600 | 46530 | 41890 | 14C/AMS 14C | 19 |
| 332 | Das Geissenklosterle | Is [sq. 130] | 9.77 | 48.4 | 550 | 34490 | 30930 | 14C/AMS 14C | 19 |
| 333 | Das Geissenklosterle | It [sq. 120] | 9.77 | 48.4 | 550 | 35250 | 31170 | 14C/AMS 14C | 19 |
| 334 | Das Geissenklosterle | It [sq. 130] | 9.77 | 48.4 | 550 | 34340 | 30900 | 14C/AMS 14C | 19 |
| 334 | Das Geissenklosterle | It [sq. 130] | 9.77 | 48.4 | 460 | 35620 | 32900 | 14C/AMS 14C | 19 |
| 335 | Das Geissenklosterle | It [sq. 33] | 9.77 | 48.4 | 500 | 35700 | 32780 | 14C/AMS 14C | 19 |
| 336 | Das Geissenklosterle | It [sq. 89] | 9.77 | 48.4 | 460 | 31570 | 30490 | 14C/AMS 14C | 19 |
| 337 | Das Geissenklosterle | It [sq. 99] | 9.77 | 48.4 | 500 | 35700 | 32740 | 14C/AMS 14C | 19 |
| 339 | Kostienki I | Cultural layer 3 | 39 | 51.29 | 5460 | 52610 | 31850 | 14C/AMS 14C | 19 |
| 344 | Kostienki XIV [Markina Gora] | Cultural layer 4: X / 40, trench III | 39.04 | 51.39 | 390 | 33340 | 30780 | 14C/AMS 14C | 19 |
| 345 | Kostienki XIV [Markina Gora] | Cultural layer 4: X / 41, trench III | 39.04 | 51.39 | 410 | 33700 | 30900 | 14C/AMS 14C | 19 |
| 450 | Castlepook Cave, Co. Cork | N/A | -8.65 | 52.28 | 220 | 25030 | 23750 | 14C/AMS 14C | 19 |
| 474 | Conningbrook Pit | N//A | 0.8853 | 51.169 | 1300 | 42240 | 35480 | 14C/AMS 14C | 19 |
| 474 | Conningbrook Pit | N//A | 0.8853 | 51.169 | 1600 | 43360 | 36840 | 14C/AMS 14C | 19 |
| 474 | Conningbrook Pit | N//A | 0.8853 | 51.169 | 1900 | 45240 | 38480 | 14C/AMS 14C | 19 |
| 474 | Conningbrook Pit | N//A | 0.8853 | 51.169 | 2400 | 46540 | 39540 | 14C/AMS 14C | 19 |
| 476 | Coygan Cave | layer 5 | -4.4962 | 51.761 | 320 | 30470 | 28710 | 14C/AMS 14C | 19 |
| 566 | Grotte Chauvet | art | 4.424 | 44.387 | 570 | 36630 | 34470 | 14C/AMS 14C | 19 |
| 566 | Grotte Chauvet | art | 4.424 | 44.387 | 600 | 37010 | 34890 | 14C/AMS 14C | 19 |
| 566 | Grotte Chauvet | art | 4.424 | 44.387 | 1500 | 39910 | 32070 | 14C/AMS 14C | 19 |
| 566 | Grotte Chauvet | art | 4.424 | 44.387 | 610 | 37150 | 34990 | 14C/AMS 14C | 19 |
| 566 | Grotte Chauvet | art | 4.424 | 44.387 | 720 | 39720 | 35880 | 14C/AMS 14C | 19 |
| 583 | Hohlenstein-Stadel IV | [?IV] | 10.168 | 48.551 | 550 | 39440 | 35560 | 14C/AMS 14C | 19 |
| 586 | Hyaena Den | N/A | -2.6761 | 51.226 | 340 | 30480 | 28560 | 14C/AMS 14C | 19 |
| 586 | Hyaena Den | N/A | -2.6761 | 51.226 | 300 | 30450 | 28730 | 14C/AMS 14C | 19 |
| 586 | Hyaena Den | N/A | -2.6761 | 51.226 | 460 | 34030 | 30910 | 14C/AMS 14C | 19 |
| 586 | Hyaena Den | N/A | -2.6761 | 51.226 | 500 | 34290 | 30970 | 14C/AMS 14C | 19 |
| 586 | Hyaena Den | N/A | -2.6761 | 51.226 | 550 | 37590 | 35470 | 14C/AMS 14C | 19 |
| 586 | Hyaena Den | N/A | -2.6761 | 51.226 | 700 | 39910 | 36230 | 14C/AMS 14C | 19 |
| 586 | Hyaena Den | N/A | -2.6761 | 51.226 | 680 | 42210 | 36290 | 14C/AMS 14C | 19 |
| 586 | Hyaena Den | N/A | -2.6761 | 51.226 | 1450 | 43100 | 36780 | 14C/AMS 14C | 19 |
| 586 | Hyaena Den | N/A | -2.6761 | 51.226 | 1200 | 44260 | 41180 | 14C/AMS 14C | 19 |
| 586 | Hyaena Den | N/A | -2.6761 | 51.226 | 1300 | 45120 | 41800 | 14C/AMS 14C | 19 |
| 586 | Hyaena Den | N/A | -2.6761 | 51.226 | 1600 | 46820 | 41940 | 14C/AMS 14C | 19 |
| 622 | Kent’s Cavern, Devon | N/A | -3.5 | 50.47 | 300 | 29410 | 27810 | 14C/AMS 14C | 19 |
| 622 | Kent’s Cavern, Devon | N/A | -3.5 | 50.47 | 340 | 31380 | 30460 | 14C/AMS 14C | 19 |
| 622 | Kent’s Cavern, Devon | N/A | -3.5 | 50.47 | 440 | 34250 | 31050 | 14C/AMS 14C | 19 |
| 622 | Kent’s Cavern, Devon | N/A | -3.5 | 50.47 | 600 | 35570 | 31290 | 14C/AMS 14C | 19 |
| 622 | Kent’s Cavern, Devon | N/A | -3.5 | 50.47 | 460 | 36350 | 34590 | 14C/AMS 14C | 19 |
| 641 | King Arthur's Cave | woolly mammoth layer | -2.6568 | 51.836 | 1500 | 43130 | 36610 | 14C/AMS 14C | 19 |
| 642 | King Arthur's Cave | red clay | -2.6568 | 51.836 | 2300 | 46260 | 39700 | 14C/AMS 14C | 19 |
| 847 | Pin Hole Cave | 37/9'-6'' (Deeper than Upper Palaeolithic) | -1.1957 | 53.264 | 2100 | 50330 | 42650 | 14C/AMS 14C | 19 |
| 848 | Pin Hole Cave | 48/8'-6'' (Deeper than Upper Palaeolithic) | -1.1957 | 53.264 | 2200 | 50500 | 42540 | 14C/AMS 14C | 19 |
| 849 | Pin Hole Cave | 50/8'-0'' (Deeper than Upper Palaeolithic) | -1.1957 | 53.264 | 2800 | 54570 | 42850 | 14C/AMS 14C | 19 |
| 852 | Pin Hole Cave | 68/6'-0'' (Deeper than Upper Palaeolithic) | -1.1957 | 53.264 | 2300 | 51910 | 43110 | 14C/AMS 14C | 19 |
| 853 | Pin Hole Cave | 69/6'-0'' (Deeper than Upper Palaeolithic) | -1.1957 | 53.264 | 1050 | 43840 | 41240 | 14C/AMS 14C | 19 |
| 854 | Pin Hole Cave | 70/7'-0'' (Deeper than Upper Palaeolithic) | -1.1957 | 53.264 | 490 | 36980 | 35140 | 14C/AMS 14C | 19 |
| 942 | Sungir' [Vladimir] | main occupation layer | 40.479 | 56.134 | 310 | 33040 | 30880 | 14C/AMS 14C | 19 |
| 943 | Sungir' [Vladimir] | Sungir' [burial] 1 | 40.479 | 56.134 | 200 | 28270 | 27270 | 14C/AMS 14C | 19 |
| 944 | Sungir' [Vladimir] | Sungir' [burial] 2 | 40.479 | 56.134 | 220 | 29420 | 28100 | 14C/AMS 14C | 19 |
| 945 | Sungir' [Vladimir] | Sungir' [burial] 3 | 40.479 | 56.134 | 240 | 29710 | 28270 | 14C/AMS 14C | 19 |
| 1077 | Paviland Cave [Goat's Hole] | occup. horizon | -4.24 | 51.55 | 340 | 28380 | 25980 | 14C/AMS 14C | 19 |
| 1077 | Paviland Cave [Goat's Hole] | occup. horizon | -4.24 | 51.55 | 400 | 29620 | 27580 | 14C/AMS 14C | 19 |
| 1077 | Paviland Cave [Goat's Hole] | occup. horizon | -4.24 | 51.55 | 1900 | 38700 | 30140 | 14C/AMS 14C | 19 |
| 583 | Hohlenstein-Stadel IV | [?IV] | 10.168 | 48.551 | 550 | 39440 | 35560 | 14C/AMS 14C | 19 |
| 140 | A. Combe Sauniere [Sarliac-sur-l'Isle] | IVB | 0.88 | 45.23 | 240 | 21770 | 20170 | 14C/AMS 14C | 19 |
| 143 | Sclayn Cave | 1A | 5 | 50.49 | 1500 | 45040 | 41360 | 14C/AMS 14C | 19 |
| 145 | Pod Hradem Cave | N/A | 16.07 | 49.04 | 470 | 39510 | 36150 | 14C/AMS 14C | 19 |
| 145 | Pod Hradem Cave | N/A | 16.07 | 49.04 | 1500 | 42310 | 34990 | 14C/AMS 14C | 19 |
| 146 | Istallosko cave | 9 | 20.04 | 48 | 600 | 39820 | 36420 | 14C/AMS 14C | 19 |
| 146 | Istallosko cave | 9 | 20.04 | 48 | 900 | 47840 | 43880 | 14C/AMS 14C | 19 |
| 147 | Tata | Culture layer | 18.3 | 47.06 | 900 | 41950 | 35990 | 14C/AMS 14C | 19 |
| 148 | Erd | d* [-2.2m] | 18.9 | 47.04 | 900 | 42670 | 38670 | 14C/AMS 14C | 19 |
| 148 | Erd | d* [-2.2m] | 18.9 | 47.04 | 830 | 44820 | 42220 | 14C/AMS 14C | 19 |
| 172 | Abri Pataud | layer 11 | 1 | 44.93 | 800 | 39680 | 35280 | 14C/AMS 14C | 19 |
| 172 | Abri Pataud | layer 11 | 1 | 44.93 | 550 | 39590 | 36270 | 14C/AMS 14C | 19 |
| 173 | Abri Pataud | Layer 7 | 1 | 44.93 | 450 | 35650 | 33130 | 14C/AMS 14C | 19 |
| 173 | Abri Pataud | Layer 7 | 1 | 44.93 | 310 | 37480 | 36080 | 14C/AMS 14C | 19 |
| 173 | Abri Pataud | Layer 7 | 1 | 44.93 | 450 | 39580 | 36540 | 14C/AMS 14C | 19 |
| 173 | Abri Pataud | Layer 7 | 1 | 44.93 | 700 | 40090 | 36330 | 14C/AMS 14C | 19 |
| 174 | Abri Pataud | Layer 5 | 1 | 44.93 | 215 | 27340 | 25420 | 14C/AMS 14C | 19 |
| 174 | Abri Pataud | Layer 5 | 1 | 44.93 | 1000 | 28930 | 23890 | 14C/AMS 14C | 19 |
| 174 | Abri Pataud | Layer 5 | 1 | 44.93 | 170 | 28550 | 27710 | 14C/AMS 14C | 19 |
| 174 | Abri Pataud | Layer 5 | 1 | 44.93 | 800 | 30300 | 26620 | 14C/AMS 14C | 19 |
| 174 | Abri Pataud | Layer 5 | 1 | 44.93 | 800 | 30490 | 26890 | 14C/AMS 14C | 19 |
| 174 | Abri Pataud | Layer 5 | 1 | 44.93 | 1000 | 30970 | 26650 | 14C/AMS 14C | 19 |
| 174 | Abri Pataud | Layer 5 | 1 | 44.93 | 330 | 31170 | 30170 | 14C/AMS 14C | 19 |
| 174 | Abri Pataud | Layer 5 | 1 | 44.93 | 310 | 31270 | 30310 | 14C/AMS 14C | 19 |
| 174 | Abri Pataud | Layer 5 | 1 | 44.93 | 450 | 31470 | 30350 | 14C/AMS 14C | 19 |
| 174 | Abri Pataud | Layer 5 | 1 | 44.93 | 200 | 31350 | 30910 | 14C/AMS 14C | 19 |
| 174 | Abri Pataud | Layer 5 | 1 | 44.93 | 460 | 31710 | 30630 | 14C/AMS 14C | 19 |
| 174 | Abri Pataud | Layer 5 | 1 | 44.93 | 320 | 33240 | 30880 | 14C/AMS 14C | 19 |
| 174 | Abri Pataud | Layer 5 | 1 | 44.93 | 260 | 33250 | 30970 | 14C/AMS 14C | 19 |
| 174 | Abri Pataud | Layer 5 | 1 | 44.93 | 225 | 33930 | 31370 | 14C/AMS 14C | 19 |
| 176 | Abri Pataud | Layer 3 | 1 | 44.93 | 280 | 22690 | 21530 | 14C/AMS 14C | 19 |
| 176 | Abri Pataud | Layer 3 | 1 | 44.93 | 160 | 26360 | 25160 | 14C/AMS 14C | 19 |
| 176 | Abri Pataud | Layer 3 | 1 | 44.93 | 140 | 28020 | 27220 | 14C/AMS 14C | 19 |
| 176 | Abri Pataud | Layer 3 | 1 | 44.93 | 170 | 28310 | 27390 | 14C/AMS 14C | 19 |
| 177 | Abri Pataud | Layer 2 | 1 | 44.93 | 170 | 23660 | 22460 | 14C/AMS 14C | 19 |
| 177 | Abri Pataud | Layer 2 | 1 | 44.93 | 300 | 24310 | 22550 | 14C/AMS 14C | 19 |
| 177 | Abri Pataud | Layer 2 | 1 | 44.93 | 170 | 24140 | 23300 | 14C/AMS 14C | 19 |
| 177 | Abri Pataud | Layer 2 | 1 | 44.93 | 200 | 25000 | 23760 | 14C/AMS 14C | 19 |
| 177 | Abri Pataud | Layer 2 | 1 | 44.93 | 140 | 25030 | 24070 | 14C/AMS 14C | 19 |
| 177 | Abri Pataud | Layer 2 | 1 | 44.93 | 170 | 25250 | 24210 | 14C/AMS 14C | 19 |
| 177 | Abri Pataud | Layer 2 | 1 | 44.93 | 170 | 25300 | 24220 | 14C/AMS 14C | 19 |
| 177 | Abri Pataud | Layer 2 | 1 | 44.93 | 220 | 25900 | 24220 | 14C/AMS 14C | 19 |
| 177 | Abri Pataud | Layer 2 | 1 | 44.93 | 340 | 26580 | 24660 | 14C/AMS 14C | 19 |
| 177 | Abri Pataud | Layer 2 | 1 | 44.93 | 250 | 27430 | 25670 | 14C/AMS 14C | 19 |
| 183 | Roche a Pierrot [St.-Cesaire] | 8 [Ejop sup.]* | 0.5 | 45.75 | 840 | 29780 | 25340 | 14C/AMS 14C | 19 |
| 184 | Grotte du Renne, Arcy-sur-Cure | XI | 3.77 | 47.59 | 1400 | 42640 | 35720 | 14C/AMS 14C | 19 |
| 192 | Roc de Marcamps [Prignac-et-Marcamps] | 8M30-N30 | -0.49 | 45.04 | 830 | 32960 | 29680 | 14C/AMS 14C | 19 |
| 193 | Jaurens [Nespouls] | Clay Cave floor | 1.52 | 45 | 1400 | 37280 | 30680 | 14C/AMS 14C | 19 |
| 193 | Jaurens [Nespouls] | Clay Cave floor | 1.52 | 45 | 1500 | 37960 | 30800 | 14C/AMS 14C | 19 |
| 193 | Jaurens [Nespouls] | Clay Cave floor | 1.52 | 45 | 3000 | 42180 | 28820 | 14C/AMS 14C | 19 |
| 193 | Jaurens [Nespouls] | Clay Cave floor | 1.52 | 45 | 2900 | 43760 | 31560 | 14C/AMS 14C | 19 |
| 197 | Gr de La Baume [Gigny sur Suran] | 10 | 5 | 46.47 | 1400 | 35220 | 29020 | 14C/AMS 14C | 19 |
| 229 | Trou Magrite | 3 | 4.97 | 50.21 | 3400 | 40130 | 25570 | 14C/AMS 14C | 19 |
| 230 | Trou Magrite | 2 | 4.97 | 50.21 | 1925 | 43210 | 35450 | 14C/AMS 14C | 19 |
| 230 | Trou Magrite | 2 | 4.97 | 50.21 | 1150 | 30100 | 24340 | 14C/AMS 14C | 19 |
| 230 | Trou Magrite | 2 | 4.97 | 50.21 | 1310 | 34620 | 28660 | 14C/AMS 14C | 19 |
| 234 | Gr du Spy | Middle Bed | 4.67 | 50.48 | 510 | 31180 | 29140 | 14C/AMS 14C | 19 |
| 235 | Gr du Spy | Arch. Bed | 4.67 | 50.48 | 455 | 27890 | 25410 | 14C/AMS 14C | 19 |
| 235 | Gr du Spy | Arch. Bed | 4.67 | 50.48 | 550 | 28080 | 25120 | 14C/AMS 14C | 19 |
| 245 | Das Geissenklosterle | II | 9.77 | 48.4 | 796 | 37240 | 34280 | 14C/AMS 14C | 19 |
| 245 | Das Geissenklosterle | II | 9.77 | 48.4 | 750 | 37590 | 34830 | 14C/AMS 14C | 19 |
| 245 | Das Geissenklosterle | II | 9.77 | 48.4 | 770 | 39120 | 34880 | 14C/AMS 14C | 19 |
| 245 | Das Geissenklosterle | II | 9.77 | 48.4 | 1000 | 39870 | 34870 | 14C/AMS 14C | 19 |
| 245 | Das Geissenklosterle | II | 9.77 | 48.4 | 480 | 39550 | 36430 | 14C/AMS 14C | 19 |
| 245 | Das Geissenklosterle | II | 9.77 | 48.4 | 825 | 42340 | 36220 | 14C/AMS 14C | 19 |
| 245 | Das Geissenklosterle | II | 9.77 | 48.4 | 3560 | 46580 | 33900 | 14C/AMS 14C | 19 |
| 246 | Vogelherd Cave | V | 10.2 | 48.56 | 1340 | 38160 | 31680 | 14C/AMS 14C | 19 |
| 246 | Vogelherd Cave | V | 10.2 | 48.56 | 560 | 36870 | 34830 | 14C/AMS 14C | 19 |
| 246 | Vogelherd Cave | V | 10.2 | 48.56 | 1100 | 40040 | 34760 | 14C/AMS 14C | 19 |
| 247 | Vogelherd Cave | IV | 10.2 | 48.56 | 750 | 37230 | 34550 | 14C/AMS 14C | 19 |
| 247 | Vogelherd Cave | IV | 10.2 | 48.56 | 1900 | 43510 | 35830 | 14C/AMS 14C | 19 |
| 248 | Lommersum | IIc | 6.8 | 50.7 | 2540 | 37370 | 26370 | 14C/AMS 14C | 19 |
| 248 | Lommersum | IIc | 6.8 | 50.7 | 850 | 36330 | 31450 | 14C/AMS 14C | 19 |
| 248 | Lommersum | IIc | 6.8 | 50.7 | 140 | 35240 | 33720 | 14C/AMS 14C | 19 |
| 248 | Lommersum | IIc | 6.8 | 50.7 | 140 | 35360 | 33960 | 14C/AMS 14C | 19 |
| 248 | Lommersum | IIc | 6.8 | 50.7 | 150 | 35540 | 34540 | 14C/AMS 14C | 19 |
| 248 | Lommersum | IIc | 6.8 | 50.7 | 1500 | 40170 | 32410 | 14C/AMS 14C | 19 |
| 248 | Lommersum | IIc | 6.8 | 50.7 | 520 | 38760 | 35360 | 14C/AMS 14C | 19 |
| 248 | Lommersum | IIc | 6.8 | 50.7 | 950 | 39820 | 34940 | 14C/AMS 14C | 19 |
| 248 | Lommersum | IIc | 6.8 | 50.7 | 320 | 38800 | 35840 | 14C/AMS 14C | 19 |
| 248 | Lommersum | IIc | 6.8 | 50.7 | 500 | 41390 | 36350 | 14C/AMS 14C | 19 |
| 252 | Krems-Hundssteig | N/A | 15.59 | 48.41 | 2000 | 43990 | 36310 | 14C/AMS 14C | 19 |
| 255 | Pavlov I | N/A | 16.69 | 48.87 | 150 | 30470 | 29510 | 14C/AMS 14C | 19 |
| 255 | Pavlov I | N/A | 16.69 | 48.87 | 350 | 31270 | 30230 | 14C/AMS 14C | 19 |
| 255 | Pavlov I | N/A | 16.69 | 48.87 | 450 | 31400 | 30240 | 14C/AMS 14C | 19 |
| 255 | Pavlov I | N/A | 16.69 | 48.87 | 460 | 31600 | 30520 | 14C/AMS 14C | 19 |
| 255 | Pavlov I | N/A | 16.69 | 48.87 | 230 | 31370 | 30890 | 14C/AMS 14C | 19 |
| 255 | Pavlov I | N/A | 16.69 | 48.87 | 230 | 31390 | 30910 | 14C/AMS 14C | 19 |
| 255 | Pavlov I | N/A | 16.69 | 48.87 | 250 | 31420 | 30940 | 14C/AMS 14C | 19 |
| 258 | Kostienki I | Cultural layer 1 | 39 | 51.29 | 620 | 23270 | 20150 | 14C/AMS 14C | 19 |
| 258 | Kostienki I | Cultural layer 1 | 39 | 51.29 | 120 | 23090 | 22370 | 14C/AMS 14C | 19 |
| 258 | Kostienki I | Cultural layer 1 | 39 | 51.29 | 580 | 24690 | 22050 | 14C/AMS 14C | 19 |
| 258 | Kostienki I | Cultural layer 1 | 39 | 51.29 | 460 | 24540 | 22300 | 14C/AMS 14C | 19 |
| 258 | Kostienki I | Cultural layer 1 | 39 | 51.29 | 200 | 24240 | 23320 | 14C/AMS 14C | 19 |
| 258 | Kostienki I | Cultural layer 1 | 39 | 51.29 | 680 | 25730 | 22290 | 14C/AMS 14C | 19 |
| 258 | Kostienki I | Cultural layer 1 | 39 | 51.29 | 300 | 25800 | 23960 | 14C/AMS 14C | 19 |
| 258 | Kostienki I | Cultural layer 1 | 39 | 51.29 | 400 | 26630 | 24470 | 14C/AMS 14C | 19 |
| 258 | Kostienki I | Cultural layer 1 | 39 | 51.29 | 700 | 28010 | 24170 | 14C/AMS 14C | 19 |
| 258 | Kostienki I | Cultural layer 1 | 39 | 51.29 | 200 | 27320 | 25520 | 14C/AMS 14C | 19 |
| 258 | Kostienki I | Cultural layer 1 | 39 | 51.29 | 310 | 27560 | 25640 | 14C/AMS 14C | 19 |
| 258 | Kostienki I | Cultural layer 1 | 39 | 51.29 | 500 | 27950 | 25190 | 14C/AMS 14C | 19 |
| 258 | Kostienki I | Cultural layer 1 | 39 | 51.29 | 200 | 27740 | 25940 | 14C/AMS 14C | 19 |
| 258 | Kostienki I | Cultural layer 1 | 39 | 51.29 | 230 | 27760 | 25920 | 14C/AMS 14C | 19 |
| 258 | Kostienki I | Cultural layer 1 | 39 | 51.29 | 250 | 28140 | 26860 | 14C/AMS 14C | 19 |
| 258 | Kostienki I | Cultural layer 1 | 39 | 51.29 | 250 | 28210 | 26970 | 14C/AMS 14C | 19 |
| 258 | Kostienki I | Cultural layer 1 | 39 | 51.29 | 200 | 28140 | 27140 | 14C/AMS 14C | 19 |
| 258 | Kostienki I | Cultural layer 1 | 39 | 51.29 | 500 | 28870 | 26590 | 14C/AMS 14C | 19 |
| 258 | Kostienki I | Cultural layer 1 | 39 | 51.29 | 300 | 28470 | 27190 | 14C/AMS 14C | 19 |
| 258 | Kostienki I | Cultural layer 1 | 39 | 51.29 | 420 | 29000 | 27160 | 14C/AMS 14C | 19 |
| 258 | Kostienki I | Cultural layer 1 | 39 | 51.29 | 680 | 29740 | 26420 | 14C/AMS 14C | 19 |
| 258 | Kostienki I | Cultural layer 1 | 39 | 51.29 | 420 | 29420 | 27340 | 14C/AMS 14C | 19 |
| 258 | Kostienki I | Cultural layer 1 | 39 | 51.29 | 200 | 28780 | 27820 | 14C/AMS 14C | 19 |
| 258 | Kostienki I | Cultural layer 1 | 39 | 51.29 | 320 | 29400 | 27720 | 14C/AMS 14C | 19 |
| 258 | Kostienki I | Cultural layer 1 | 39 | 51.29 | 200 | 29310 | 28070 | 14C/AMS 14C | 19 |
| 258 | Kostienki I | Cultural layer 1 | 39 | 51.29 | 500 | 30240 | 27880 | 14C/AMS 14C | 19 |
| 259 | Kostienki XIV [Markina Gora] | Cultural layer 2 | 39.04 | 51.39 | 310 | 30720 | 29320 | 14C/AMS 14C | 19 |
| 259 | Kostienki XIV [Markina Gora] | Cultural layer 2 | 39.04 | 51.39 | 660 | 31830 | 30070 | 14C/AMS 14C | 19 |
| 259 | Kostienki XIV [Markina Gora] | Cultural layer 2 | 39.04 | 51.39 | 700 | 35080 | 30840 | 14C/AMS 14C | 19 |
| 259 | Kostienki XIV [Markina Gora] | Cultural layer 2 | 39.04 | 51.39 | 220 | 34290 | 31650 | 14C/AMS 14C | 19 |
| 300 | Istallosko cave | 8? | 20.04 | 48 | 600 | 37100 | 34980 | 14C/AMS 14C | 19 |
| 300 | Istallosko cave | 8? | 20.04 | 48 | 600 | 38140 | 35340 | 14C/AMS 14C | 19 |
| 301 | Istallosko cave | 9 [base] | 20.04 | 48 | 1900 | 51570 | 43810 | 14C/AMS 14C | 19 |
| 302 | Istallosko cave | 9 [top] | 20.04 | 48 | 900 | 45070 | 42350 | 14C/AMS 14C | 19 |
| 303 | Erd | e* [-2.4m] | 18.9 | 47.04 | 1400 | 50700 | 44380 | 14C/AMS 14C | 19 |
| 316 | Trou Magrite | 2 base | 4.97 | 50.21 | 2200 | 40520 | 29840 | 14C/AMS 14C | 19 |
| 316 | Trou Magrite | 2 base | 4.97 | 50.21 | 1925 | 43210 | 35450 | 14C/AMS 14C | 19 |
| 326 | Das Geissenklosterle | [IIIa] | 9.77 | 48.4 | 1000 | 42550 | 36630 | 14C/AMS 14C | 19 |
| 327 | Das Geissenklosterle | Ia | 9.77 | 48.4 | 290 | 29310 | 27750 | 14C/AMS 14C | 19 |
| 328 | Das Geissenklosterle | III | 9.77 | 48.4 | 1570 | 44210 | 38330 | 14C/AMS 14C | 19 |
| 338 | Kulna Cave | 7a | 16.75 | 49.41 | 2850 | 55650 | 43210 | 14C/AMS 14C | 19 |
| 339 | Kostienki I | Cultural layer 3 | 39 | 51.29 | 1800 | 34650 | 26530 | 14C/AMS 14C | 19 |
| 339 | Kostienki I | Cultural layer 3 | 39 | 51.29 | 400 | 31220 | 30100 | 14C/AMS 14C | 19 |
| 339 | Kostienki I | Cultural layer 3 | 39 | 51.29 | 2200 | 35770 | 25810 | 14C/AMS 14C | 19 |
| 339 | Kostienki I | Cultural layer 3 | 39 | 51.29 | 400 | 39470 | 36430 | 14C/AMS 14C | 19 |
| 340 | Kostienki I | Cultural layer 3, sq. D 72 | 39 | 51.29 | 1300 | 31680 | 26560 | 14C/AMS 14C | 19 |
| 341 | Kostienki I | Cultural layer 3, sq. D 74 | 39 | 51.29 | 1500 | 34590 | 27750 | 14C/AMS 14C | 19 |
| 342 | Kostienki I | Cultural layer 3, sq. E 72 | 39 | 51.29 | 1000 | 31910 | 28430 | 14C/AMS 14C | 19 |
| 343 | Kostienki XIV [Markina Gora] | Cultural layer 1 | 39.04 | 51.39 | 250 | 28230 | 26990 | 14C/AMS 14C | 19 |
| 346 | Kostienki XIV [Markina Gora] | Cultural layer 4a | 39.04 | 51.39 | 650 | 41300 | 36260 | 14C/AMS 14C | 19 |
| 348 | [Gr.] Neron [Soyons] | IV [sq. 1] | 4.836 | 44.891 | 1100 | 48960 | 43960 | 14C/AMS 14C | 19 |
| 422 | Bodrogkeresztur [Henye Hill] | N/A | 21.3864 | 48.125 | 3000 | 40080 | 27560 | 14C/AMS 14C | 19 |
| 448 | Camiac[-et-St-Denis] | basal layer [D] | -0.2655 | 44.787 | 2000 | 43780 | 35900 | 14C/AMS 14C | 19 |
| 475 | Coygan Cave | calcrete 1 [below Mousterian] | -4.4962 | 51.761 | 310 | 39800 | 36760 | 14C/AMS 14C | 19 |
| 477 | Coygan Cave | N/A | -4.4962 | 51.761 | 2713 | 47360 | 38600 | 14C/AMS 14C | 19 |
| 480 | Cueto de la Mina | N/A | -4.84 | 43.42 | 520 | 31620 | 30340 | 14C/AMS 14C | 19 |
| 486 | Dolni Vestonice | II | 16.628 | 48.882 | 743 | 28310 | 24350 | 14C/AMS 14C | 19 |
| 486 | Dolni Vestonice | II | 16.628 | 48.882 | 749 | 28790 | 24950 | 14C/AMS 14C | 19 |
| 486 | Dolni Vestonice | II | 16.628 | 48.882 | 420 | 28530 | 25850 | 14C/AMS 14C | 19 |
| 486 | Dolni Vestonice | II | 16.628 | 48.882 | 870 | 30630 | 26710 | 14C/AMS 14C | 19 |
| 486 | Dolni Vestonice | II | 16.628 | 48.882 | 900 | 30820 | 26900 | 14C/AMS 14C | 19 |
| 486 | Dolni Vestonice | II | 16.628 | 48.882 | 2100 | 32850 | 23770 | 14C/AMS 14C | 19 |
| 486 | Dolni Vestonice | II | 16.628 | 48.882 | 190 | 30220 | 28740 | 14C/AMS 14C | 19 |
| 486 | Dolni Vestonice | II | 16.628 | 48.882 | 920 | 31460 | 27900 | 14C/AMS 14C | 19 |
| 486 | Dolni Vestonice | II | 16.628 | 48.882 | 210 | 31030 | 30230 | 14C/AMS 14C | 19 |
| 486 | Dolni Vestonice | II | 16.628 | 48.882 | 100 | 31160 | 30600 | 14C/AMS 14C | 19 |
| 486 | Dolni Vestonice | II | 16.628 | 48.882 | 190 | 31280 | 30760 | 14C/AMS 14C | 19 |
| 486 | Dolni Vestonice | II | 16.628 | 48.882 | 270 | 31340 | 30660 | 14C/AMS 14C | 19 |
| 486 | Dolni Vestonice | II | 16.628 | 48.882 | 160 | 31310 | 30910 | 14C/AMS 14C | 19 |
| 486 | Dolni Vestonice | II | 16.628 | 48.882 | 110 | 31320 | 31000 | 14C/AMS 14C | 19 |
| 486 | Dolni Vestonice | II | 16.628 | 48.882 | 250 | 31510 | 31030 | 14C/AMS 14C | 19 |
| 486 | Dolni Vestonice | II | 16.628 | 48.882 | 160 | 31460 | 31100 | 14C/AMS 14C | 19 |
| 486 | Dolni Vestonice | II | 16.628 | 48.882 | 200 | 31490 | 31090 | 14C/AMS 14C | 19 |
| 486 | Dolni Vestonice | II | 16.628 | 48.882 | 170 | 31510 | 31150 | 14C/AMS 14C | 19 |
| 486 | Dolni Vestonice | II | 16.628 | 48.882 | 300 | 31770 | 31010 | 14C/AMS 14C | 19 |
| 486 | Dolni Vestonice | II | 16.628 | 48.882 | 170 | 31530 | 31130 | 14C/AMS 14C | 19 |
| 486 | Dolni Vestonice | II | 16.628 | 48.882 | 80 | 32430 | 31230 | 14C/AMS 14C | 19 |
| 486 | Dolni Vestonice | II | 16.628 | 48.882 | 550 | 34280 | 30880 | 14C/AMS 14C | 19 |
| 486 | Dolni Vestonice | II | 16.628 | 48.882 | 370 | 34320 | 31240 | 14C/AMS 14C | 19 |
| 486 | Dolni Vestonice | II | 16.628 | 48.882 | 300 | 34300 | 31420 | 14C/AMS 14C | 19 |
| 486 | Dolni Vestonice | II | 16.628 | 48.882 | 200 | 35110 | 33350 | 14C/AMS 14C | 19 |
| 486 | Dolni Vestonice | II | 16.628 | 48.882 | 300 | 35930 | 34530 | 14C/AMS 14C | 19 |
| 487 | Dolni Vestonice | III | 16.628 | 48.882 | 660 | 30840 | 28000 | 14C/AMS 14C | 19 |
| 488 | Dolni Vestonice | I | 16.628 | 48.882 | 540 | 24710 | 22190 | 14C/AMS 14C | 19 |
| 488 | Dolni Vestonice | I | 16.628 | 48.882 | 210 | 24950 | 23630 | 14C/AMS 14C | 19 |
| 488 | Dolni Vestonice | I | 16.628 | 48.882 | 570 | 28260 | 25300 | 14C/AMS 14C | 19 |
| 488 | Dolni Vestonice | I | 16.628 | 48.882 | 320 | 31160 | 30160 | 14C/AMS 14C | 19 |
| 488 | Dolni Vestonice | I | 16.628 | 48.882 | 170 | 31060 | 30300 | 14C/AMS 14C | 19 |
| 488 | Dolni Vestonice | I | 16.628 | 48.882 | 630 | 31480 | 29800 | 14C/AMS 14C | 19 |
| 488 | Dolni Vestonice | I | 16.628 | 48.882 | 190 | 31290 | 30810 | 14C/AMS 14C | 19 |
| 488 | Dolni Vestonice | I | 16.628 | 48.882 | 590 | 33620 | 30500 | 14C/AMS 14C | 19 |
| 488 | Dolni Vestonice | I | 16.628 | 48.882 | 370 | 33700 | 30980 | 14C/AMS 14C | 19 |
| 488 | Dolni Vestonice | I | 16.628 | 48.882 | 460 | 35600 | 32880 | 14C/AMS 14C | 19 |
| 488 | Dolni Vestonice | I | 16.628 | 48.882 | 750 | 36220 | 31940 | 14C/AMS 14C | 19 |
| 488 | Dolni Vestonice | I | 16.628 | 48.882 | 1000 | 39770 | 34690 | 14C/AMS 14C | 19 |
| 488 | Dolni Vestonice | I | 16.628 | 48.882 | 660 | 39940 | 36340 | 14C/AMS 14C | 19 |
| 489 | Dolni Vestonice | XVI | 16.628 | 48.882 | 280 | 31000 | 30080 | 14C/AMS 14C | 19 |
| 552 | Gr. de la Princesse [Marche-les-Dames] | Aurig. II bed | 4.972 | 50.483 | 500 | 29580 | 27140 | 14C/AMS 14C | 19 |
| 583 | Hohlenstein-Stadel IV | [?IV] | 10.168 | 48.551 | 1150 | 40050 | 34490 | 14C/AMS 14C | 19 |
| 660 | Korman' IV | VII (layer 7) | 27.28 | 48.27 | 500 | 30630 | 28190 | 14C/AMS 14C | 19 |
| 660 | Korman' IV | VII (layer 7) | 27.28 | 48.27 | 350 | 30840 | 29280 | 14C/AMS 14C | 19 |
| 662 | Kostienki XI [Anosovka site 2] | cultural layer 2 | 39 | 51.29 | 200 | 27320 | 25520 | 14C/AMS 14C | 19 |
| 663 | Kostienki XI [Anosovka site 2] | cultural layer 3 [upper] | 39 | 51.29 | 120 | 19540 | 18940 | 14C/AMS 14C | 19 |
| 663 | Kostienki XI [Anosovka site 2] | cultural layer 3 [upper] | 39 | 51.29 | 340 | 28390 | 26630 | 14C/AMS 14C | 19 |
| 666 | Kostienki XVII [Spitsyn site] | N/A | 39 | 51.29 | 350 | 24730 | 23090 | 14C/AMS 14C | 19 |
| 668 | Kostienki XXI [Gmelinskaya] | Cultural layer 3 | 39 | 51.29 | 300 | 20940 | 19460 | 14C/AMS 14C | 19 |
| 668 | Kostienki XXI [Gmelinskaya] | Cultural layer 3 | 39 | 51.29 | 340 | 26400 | 24560 | 14C/AMS 14C | 19 |
| 668 | Kostienki XXI [Gmelinskaya] | Cultural layer 3 | 39 | 51.29 | 150 | 27650 | 25970 | 14C/AMS 14C | 19 |
| 670 | Kostienki XXI [Gmelinskaya] | N/A | 39 | 51.29 | 340 | 27460 | 24700 | 14C/AMS 14C | 19 |
| 670 | Kostienki XXI [Gmelinskaya] | N/A | 39 | 51.29 | 150 | 27650 | 25970 | 14C/AMS 14C | 19 |
| 690 | La Balme d'Epy [Jura] | [underground] river deposit | 5.428 | 46.382 | 1900 | 29130 | 19770 | 14C/AMS 14C | 19 |
| 695 | La Quina Y-Z [Villebois la Valette] | 1 | 0.303 | 45.504 | 490 | 36850 | 35010 | 14C/AMS 14C | 19 |
| 695 | La Quina Y-Z [Villebois la Valette] | 1 | 0.303 | 45.504 | 350 | 37120 | 35720 | 14C/AMS 14C | 19 |
| 701 | Laugerie-Haute Ouest | 2 | 0.95 | 44.967 | 240 | 24420 | 23380 | 14C/AMS 14C | 19 |
| 702 | Laugerie-Haute Ouest | 12a | 0.95 | 44.967 | 100 | 24350 | 23790 | 14C/AMS 14C | 19 |
| 702 | Laugerie-Haute Ouest | 12a | 0.95 | 44.967 | 230 | 25570 | 24090 | 14C/AMS 14C | 19 |
| 703 | Laugerie-Haute Ouest | 12d | 0.95 | 44.967 | 150 | 25170 | 24210 | 14C/AMS 14C | 19 |
| 709 | Les Cottes [St. Pierre de Maille] | 2 | 0.851 | 47.7 | 150 | 18030 | 17310 | 14C/AMS 14C | 19 |
| 714 | Les Cottes [St. Pierre de Maille] | I1 | 0.851 | 47.7 | 400 | 39460 | 36060 | 14C/AMS 14C | 19 |
| 715 | Les Cottes [St. Pierre de Maille] | I2 | 0.851 | 47.7 | 700 | 43500 | 41660 | 14C/AMS 14C | 19 |
| 742 | Maisieres-Canal | clay layer | 3.987 | 50.473 | 1040 | 31800 | 27960 | 14C/AMS 14C | 19 |
| 743 | Maisieres-Canal | humic bed 2 | 3.987 | 50.473 | 700 | 30790 | 27790 | 14C/AMS 14C | 19 |
| 744 | Maisieres-Canal | occup. Horizon | 3.987 | 50.473 | 650 | 30510 | 27590 | 14C/AMS 14C | 19 |
| 744 | Maisieres-Canal | occup. Horizon | 3.987 | 50.473 | 1890 | 39640 | 30480 | 14C/AMS 14C | 19 |
| 744 | Maisieres-Canal | occup. Horizon | 3.987 | 50.473 | 2040 | 41310 | 31230 | 14C/AMS 14C | 19 |
| 744 | Maisieres-Canal | occup. Horizon | 3.987 | 50.473 | 3140 | 45920 | 34640 | 14C/AMS 14C | 19 |
| 745 | Maisieres-Canal | palaeosol | 3.987 | 50.473 | 400 | 36770 | 35130 | 14C/AMS 14C | 19 |
| 766 | Milovice I | [base of overlying loess] | 16.73 | 48.842 | 1100 | 29250 | 23770 | 14C/AMS 14C | 19 |
| 766 | Milovice I | [base of overlying loess] | 16.73 | 48.842 | 490 | 28790 | 26350 | 14C/AMS 14C | 19 |
| 766 | Milovice I | [base of overlying loess] | 16.73 | 48.842 | 280 | 30810 | 29530 | 14C/AMS 14C | 19 |
| 767 | Milovice I | section L [base of overlying loess] | 16.73 | 48.842 | 1100 | 29250 | 23770 | 14C/AMS 14C | 19 |
| 768 | Milovice I | trench D [2nd interpleniglacial soil] | 16.73 | 48.842 | 950 | 36480 | 31240 | 14C/AMS 14C | 19 |
| 773 | Molodova V [Kosoutsy] | 10 | 27.28 | 48.27 | 400 | 28710 | 27070 | 14C/AMS 14C | 19 |
| 776 | Montagne de Girault [Genay] | tranchee 2 [East] | 4.2833 | 47.533 | 1000 | 34760 | 30120 | 14C/AMS 14C | 19 |
| 779 | Moravany-Lopata II | Concentration A | 17.8863 | 48.609 | 610 | 27640 | 24000 | 14C/AMS 14C | 19 |
| 780 | Moravany-Lopata II | Concentration B | 17.8863 | 48.609 | 800 | 30720 | 27280 | 14C/AMS 14C | 19 |
| 844 | Picken's Hole, Layer 3 | N/A | -2.8778 | 51.291 | 2440 | 37600 | 27480 | 14C/AMS 14C | 19 |
| 861 | Pod Hradem Cave A | 8 | 16.721 | 49.386 | 220 | 33980 | 31460 | 14C/AMS 14C | 19 |
| 861 | Pod Hradem Cave A | 8 | 16.721 | 49.386 | 230 | 35450 | 33810 | 14C/AMS 14C | 19 |
| 861 | Pod Hradem Cave A | 8 | 16.721 | 49.386 | 530 | 40000 | 36600 | 14C/AMS 14C | 19 |
| 861 | Pod Hradem Cave A | 8 | 16.721 | 49.386 | 1100 | 42130 | 35770 | 14C/AMS 14C | 19 |
| 898 | Salzgitter-Lebenstedt | [fluviatile sediments] | 10.3306 | 52.182 | 900 | 60910 | 54790 | 14C/AMS 14C | 19 |
| 940 | Sungir' [Vladimir] | -3.2m | 40.479 | 56.134 | 400 | 30470 | 28270 | 14C/AMS 14C | 19 |
| 940 | Sungir' [Vladimir] | -3.2m | 40.479 | 56.134 | 200 | 30880 | 30120 | 14C/AMS 14C | 19 |
| 941 | Sungir' [Vladimir] | c.i | 40.479 | 56.134 | 120 | 25010 | 24090 | 14C/AMS 14C | 19 |
| 941 | Sungir' [Vladimir] | c.i | 40.479 | 56.134 | 1000 | 28690 | 23690 | 14C/AMS 14C | 19 |
| 941 | Sungir' [Vladimir] | c.i | 40.479 | 56.134 | 600 | 28630 | 25470 | 14C/AMS 14C | 19 |
| 941 | Sungir' [Vladimir] | c.i | 40.479 | 56.134 | 400 | 30470 | 28270 | 14C/AMS 14C | 19 |
| 941 | Sungir' [Vladimir] | c.i | 40.479 | 56.134 | 200 | 30880 | 30120 | 14C/AMS 14C | 19 |
| 946 | Sungir' [Vladimir] | N/A | 40.479 | 56.134 | 600 | 19060 | 16820 | 14C/AMS 14C | 19 |
| 946 | Sungir' [Vladimir] | N/A | 40.479 | 56.134 | 400 | 20340 | 18620 | 14C/AMS 14C | 19 |
| 946 | Sungir' [Vladimir] | N/A | 40.479 | 56.134 | 80 | 23950 | 23350 | 14C/AMS 14C | 19 |
| 946 | Sungir' [Vladimir] | N/A | 40.479 | 56.134 | 500 | 33930 | 30810 | 14C/AMS 14C | 19 |
| 950 | Szeleta Cave | 4 | 20.6312 | 48.119 | 420 | 39480 | 36280 | 14C/AMS 14C | 19 |
| 951 | Szeleta Cave | 3 [-6m] - dark brown layer | 20.6312 | 48.119 | 1100 | 48960 | 43960 | 14C/AMS 14C | 19 |
| 952 | Szeleta Cave | 7 [-3m] - light grey layer | 20.6312 | 48.119 | 400 | 39480 | 36440 | 14C/AMS 14C | 19 |
| 1012 | Weinberghohlen [Mauern 2] | -0.7m [2*] | 11.065 | 48.773 | 470 | 35760 | 33240 | 14C/AMS 14C | 19 |
| 1013 | Weinberghohlen [Mauern 2] | D [-1.5m] [2*] | 11.065 | 48.773 | 325 | 34280 | 31360 | 14C/AMS 14C | 19 |
| 1073 | Kostienki XVII [Spitsyn site] | Cultural layer 1 | 39 | 51.29 | 700 | 33040 | 30040 | 14C/AMS 14C | 19 |
| 1074 | Kostienki XVII [Spitsyn site] | Cultural layer 2 | 39 | 51.29 | 200 | 24470 | 23510 | 14C/AMS 14C | 19 |
| 1074 | Kostienki XVII [Spitsyn site] | Cultural layer 2 | 39 | 51.29 | 2000 | 42360 | 33080 | 14C/AMS 14C | 19 |
| 1074 | Kostienki XVII [Spitsyn site] | Cultural layer 2 | 39 | 51.29 | 300 | 39450 | 36650 | 14C/AMS 14C | 19 |
| 1074 | Kostienki XVII [Spitsyn site] | Cultural layer 2 | 39 | 51.29 | 1700 | 44220 | 38020 | 14C/AMS 14C | 19 |
| 1075 | Kostienki XXI [Gmelinskaya] | Cultural layer 2 | 39 | 51.29 | 130 | 23410 | 22290 | 14C/AMS 14C | 19 |
| 1075 | Kostienki XXI [Gmelinskaya] | Cultural layer 2 | 39 | 51.29 | 150 | 23540 | 22300 | 14C/AMS 14C | 19 |
| 1075 | Kostienki XXI [Gmelinskaya] | Cultural layer 2 | 39 | 51.29 | 100 | 24440 | 23920 | 14C/AMS 14C | 19 |
| 1075 | Kostienki XXI [Gmelinskaya] | Cultural layer 2 | 39 | 51.29 | 150 | 28180 | 27300 | 14C/AMS 14C | 19 |
| 583 | Hohlenstein-Stadel IV | [?IV] | 10.168 | 48.551 | 1150 | 40050 | 34490 | 14C/AMS 14C | 19 |
| 199 | La Chapelle-aux-Saints | bed 1 [yellow clay with limestone fragments] | 1.72 | 44.95 | 3000 | 50000 | 44000 | ESR: EU | 19 |
| 199 | La Chapelle-aux-Saints | bed 1 [yellow clay with limestone fragments] | 1.72 | 44.95 | 4000 | 60000 | 52000 | ESR: LU | 19 |
| 775 | Montagne de Girault [Genay] | tranchee 2 (red series: levels 3 & 4) | 4.2833 | 47.533 | 6000 | 81000 | 69000 | Pa-231/U-235 | 19 |
| 775 | Montagne de Girault [Genay] | tranchee 2 (red series: levels 3 & 4) | 4.2833 | 47.533 | 20000 | 102000 | 62000 | Th-230/U-234 | 19 |
| 143 | Sclayn Cave | 1A | 5 | 50.49 | 5500 | 49500 | 38500 | TL | 19 |
| 183 | Roche a Pierrot [St.-Cesaire] | 8 [Ejop sup.]* | 0.5 | 45.75 | 5400 | 39100 | 28300 | TL | 19 |
| 183 | Roche a Pierrot [St.-Cesaire] | 8 [Ejop sup.]* | 0.5 | 45.75 | 4600 | 40200 | 31000 | TL | 19 |
| 183 | Roche a Pierrot [St.-Cesaire] | 8 [Ejop sup.]* | 0.5 | 45.75 | 2700 | 39000 | 33600 | TL | 19 |
| 183 | Roche a Pierrot [St.-Cesaire] | 8 [Ejop sup.]* | 0.5 | 45.75 | 4900 | 41500 | 31700 | TL | 19 |
| 183 | Roche a Pierrot [St.-Cesaire] | 8 [Ejop sup.]* | 0.5 | 45.75 | 5000 | 41600 | 31600 | TL | 19 |
| 183 | Roche a Pierrot [St.-Cesaire] | 8 [Ejop sup.]* | 0.5 | 45.75 | 5200 | 42600 | 32200 | TL | 19 |
| 183 | Roche a Pierrot [St.-Cesaire] | 8 [Ejop sup.]* | 0.5 | 45.75 | 5300 | 43500 | 32900 | TL | 19 |
| 245 | Das Geissenklosterle | II | 9.77 | 48.4 | 3900 | 39500 | 31700 | TL | 19 |
| 245 | Das Geissenklosterle | II | 9.77 | 48.4 | 2500 | 40600 | 35600 | TL | 19 |
| 328 | Das Geissenklosterle | III | 9.77 | 48.4 | 5400 | 43700 | 32900 | TL | 19 |
| 328 | Das Geissenklosterle | III | 9.77 | 48.4 | 3900 | 42600 | 34800 | TL | 19 |
| 328 | Das Geissenklosterle | III | 9.77 | 48.4 | 2100 | 41600 | 37400 | TL | 19 |
| 328 | Das Geissenklosterle | III | 9.77 | 48.4 | 4700 | 45600 | 36200 | TL | 19 |
| 328 | Das Geissenklosterle | III | 9.77 | 48.4 | 5400 | 49000 | 38200 | TL | 19 |
| 328 | Das Geissenklosterle | III | 9.77 | 48.4 | 5600 | 50300 | 39100 | TL | 19 |
| 328 | Das Geissenklosterle | III | 9.77 | 48.4 | 3800 | 65400 | 57800 | TL | 19 |
| 523 | Fonseigner [Bourdeilles] | D [mid] | 0.6095 | 45.341 | 5500 | 58300 | 47300 | TL | 19 |
| 524 | Fonseigner [Bourdeilles] | D [upper] | 0.6095 | 45.341 | 5300 | 55500 | 44900 | TL | 19 |
| 525 | Fonseigner [Bourdeilles] | E | 0.6095 | 45.341 | 6800 | 63200 | 49600 | TL | 19 |
| 669 | Kostienki XXI [Gmelinskaya] | Cultural layer 3 | 39 | 51.29 | 2000 | 28765 | 24765 | TL | 19 |
| 303 | Erd | e* [-2.4m] | 18.9 | 47.04 | 1400 | 50700 | 44380 | 14C/AMS 14C | 19 |
| 228 | Shandon Cave | N/A | -7.63 | 52.01 | 550 | 36990 | 34990 | 14C/AMS 14C | 19; 13 |
| 474 | Conningbrook Pit | N//A | 0.8853 | 51.169 | 1200 | 42460 | 35860 | 14C/AMS 14C | 19 |
| 474 | Conningbrook Pit | N//A | 0.8853 | 51.169 | 1400 | 42760 | 35920 | 14C/AMS 14C | 19 |
| 474 | Conningbrook Pit | N//A | 0.8853 | 51.169 | 1500 | 43170 | 36810 | 14C/AMS 14C | 19 |
| 86 | Gönnersdorf | N/A | 7.42 | 50.45 | 650 | 14590 | 11230 | 14C/AMS 14C | 19 |
| 86 | Gönnersdorf | N/A | 7.42 | 50.45 | 230 | 15390 | 13710 | 14C/AMS 14C | 19 |
| 100 | Alberndorf | N/A | 15 | 48.5 | 900 | 24270 | 19710 | 14C/AMS 14C | 19 |
| 100 | Alberndorf | N/A | 15 | 48.5 | 500 | 31410 | 30130 | 14C/AMS 14C | 19 |
| 101 | Risovaca | N/A | 20.57 | 44.28 | 1800 | 36360 | 28840 | 14C/AMS 14C | 19 |
| 101 | Risovaca | N/A | 20.57 | 44.28 | 2800 | 42610 | 29810 | 14C/AMS 14C | 19 |
| 103 | Bora gran d'en carreres | N/A | 2.87 | 41.87 | 500 | 14810 | 12370 | 14C/AMS 14C | 19 |
| 140 | A. Combe Sauniere [Sarliac-sur-l'Isle] | IVB | 0.88 | 45.23 | 250 | 21790 | 20150 | 14C/AMS 14C | 19 |
| 146 | Istallosko cave | 9 | 20.04 | 48 | 1900 | 51570 | 43810 | 14C/AMS 14C | 19 |
| 146 | Istallosko cave | 9 | 20.04 | 48 | 900 | 45070 | 42350 | 14C/AMS 14C | 19 |
| 225 | Soldier's Hole | Spit 8-20 | -2.77 | 51.28 | 3500 | 51370 | 39610 | 14C/AMS 14C | 19 |
| 225 | Soldier's Hole | Spit 8-20 | -2.77 | 51.28 | 550 | 31820 | 30380 | 14C/AMS 14C | 19 |
| 232 | Trou Walou | C6C | 5.72 | 50.59 | 640 | 36070 | 32590 | 14C/AMS 14C | 19 |
| 232 | Trou Walou | C6C | 5.72 | 50.59 | 760 | 36610 | 32890 | 14C/AMS 14C | 19 |
| 235 | Gr du Spy | Arch. Bed | 4.67 | 50.48 | 550 | 28080 | 25120 | 14C/AMS 14C | 19 |
| 248 | Lommersum | IIc | 6.8 | 50.7 | 500 | 41390 | 36350 | 14C/AMS 14C | 19 |
| 248 | Lommersum | IIc | 6.8 | 50.7 | 140 | 35360 | 33960 | 14C/AMS 14C | 19 |
| 254 | Kulna Cave | 6a | 16.75 | 49.41 | 900 | 46680 | 50640 | 14C/AMS 14C | 19 |
| 258 | Kostienki I | Cultural layer 1 | 39 | 51.29 | 500 | 30240 | 27880 | 14C/AMS 14C | 19 |
| 258 | Kostienki I | Cultural layer 1 | 39 | 51.29 | 620 | 23270 | 20150 | 14C/AMS 14C | 19 |
| 259 | Kostienki XIV [Markina Gora] | Cultural layer 2 | 39.04 | 51.39 | 420 | 34990 | 31390 | 14C/AMS 14C | 19 |
| 259 | Kostienki XIV [Markina Gora] | Cultural layer 2 | 39.04 | 51.39 | 310 | 30720 | 29320 | 14C/AMS 14C | 19 |
| 260 | Moldova V [Kosoutsy] | 7 | 27.28 | 48.27 | 800 | 29750 | 25470 | 14C/AMS 14C | 19 |
| 260 | Moldova V [Kosoutsy] | 7 | 27.28 | 48.27 | 320 | 29470 | 27790 | 14C/AMS 14C | 19 |
| 245 | Das Geissenklosterle | II | 9.77 | 48.4 | 1000 | 43350 | 40590 | 14C/AMS 14C | 19 |
| 245 | Das Geissenklosterle | II | 9.77 | 48.4 | 796 | 37240 | 34280 | 14C/AMS 14C | 19 |
| 164 | Pin Hole | Lower Cave Earth | -1.19 | 53.26 | 1000 | 28930 | 23890 | 14C/AMS 14C | 19 |
| 164 | Pin Hole | Lower Cave Earth | -1.19 | 53.26 | 1000 | 40070 | 35230 | 14C/AMS 14C | 19 |
| 223 | Brean Down | N/A | -3.02 | 51.32 | 1600 | 47800 | 42240 | 14C/AMS 14C | 19 |
| 228 | Shandon Cave | N/A | -7.63 | 52.01 | 670 | 39670 | 35950 | 14C/AMS 14C | 19 |
| 228 | Shandon Cave | N/A | -7.63 | 52.01 | 420 | 33470 | 30790 | 14C/AMS 14C | 19 |
| 231 | Trou Magrite | 4a | 4.97 | 50.21 | 190 | 26470 | 25070 | 14C/AMS 14C | 19 |
| 231 | Trou Magrite | 4a | 4.97 | 50.21 | 600 | 37090 | 34970 | 14C/AMS 14C | 19 |
| 2 | Labeko Koba | VII | -2.5 | 43.07 | 530 | 32590 | 30510 | 14C/AMS 14C | 19 |
| 2 | Labeko Koba | VII | -2.5 | 43.07 | 915 | 39340 | 34580 | 14C/AMS 14C | 19 |
| 132 | Roc de Combe [Nadaillac] | 6 | 1.33 | 44.07 | 1200 | 32220 | 27860 | 14C/AMS 14C | 19 |
| 132 | Roc de Combe [Nadaillac] | 6 | 1.33 | 44.07 | 500 | 33680 | 30720 | 14C/AMS 14C | 19 |
| 133 | Roc de Combe [Nadaillac] | 1a-c | 1.33 | 44.07 | 1000 | 39940 | 35020 | 14C/AMS 14C | 19 |
| 133 | Roc de Combe [Nadaillac] | 1a-c | 1.33 | 44.07 | 400 | 31060 | 29380 | 14C/AMS 14C | 19 |
| 145 | Pod Hradem Cave | N/A | 16.07 | 49.04 | 1500 | 42310 | 34990 | 14C/AMS 14C | 19 |
| 145 | Pod Hradem Cave | N/A | 16.07 | 49.04 | 470 | 39510 | 36150 | 14C/AMS 14C | 19 |
| 147 | Tata | Culture layer | 18.3 | 47.06 | 900 | 41950 | 35990 | 14C/AMS 14C | 19 |
| 148 | Erd | d* [-2.2m] | 18.9 | 47.04 | 900 | 42670 | 38670 | 14C/AMS 14C | 19 |
| 148 | Erd | d* [-2.2m] | 18.9 | 47.04 | 830 | 44820 | 42220 | 14C/AMS 14C | 19 |
| 172 | Abri Pataud | layer 11 | 1 | 44.93 | 800 | 39680 | 35280 | 14C/AMS 14C | 19 |
| 172 | Abri Pataud | layer 11 | 1 | 44.93 | 550 | 39590 | 36270 | 14C/AMS 14C | 19 |
| 173 | Abri Pataud | Layer 7 | 1 | 44.93 | 450 | 39580 | 36540 | 14C/AMS 14C | 19 |
| 173 | Abri Pataud | Layer 7 | 1 | 44.93 | 310 | 37480 | 36080 | 14C/AMS 14C | 19 |
| 174 | Abri Pataud | Layer 5 | 1 | 44.93 | 1100 | 35950 | 30550 | 14C/AMS 14C | 19 |
| 174 | Abri Pataud | Layer 5 | 1 | 44.93 | 215 | 27340 | 25420 | 14C/AMS 14C | 19 |
| 177 | Abri Pataud | Layer 2 | 1 | 44.93 | 340 | 26580 | 24660 | 14C/AMS 14C | 19 |
| 177 | Abri Pataud | Layer 2 | 1 | 44.93 | 300 | 24310 | 22550 | 14C/AMS 14C | 19 |
| 187 | La Quina | 1 | 0.3 | 45.5 | 350 | 37120 | 35720 | 14C/AMS 14C | 19 |
| 187 | La Quina | 1 | 0.3 | 45.5 | 490 | 36850 | 35010 | 14C/AMS 14C | 19 |
| 192 | Roc de Marcamps [Prignac-et-Marcamps] | 8M30-N30 | -0.49 | 45.04 | 830 | 32960 | 29680 | 14C/AMS 14C | 19 |
| 193 | Jaurens [Nespouls] | Clay Cave floor | 1.52 | 45 | 2900 | 43760 | 31560 | 14C/AMS 14C | 19 |
| 193 | Jaurens [Nespouls] | Clay Cave floor | 1.52 | 45 | 1400 | 37280 | 30680 | 14C/AMS 14C | 19 |
| 197 | Gr de La Baume [Gigny sur Suran] | 10 | 5 | 46.47 | 1400 | 35220 | 29020 | 14C/AMS 14C | 19 |
| 221 | Paviland Cave [Goat's Hole] | N/A | -4.24 | 51.55 | 120 | 18830 | 18110 | 14C/AMS 14C | 19 |
| 226 | Picken's Hole | Layer3 | -2.88 | 51.29 | 2600 | 44100 | 34660 | 14C/AMS 14C | 19 |
| 226 | Picken's Hole | Layer3 | -2.88 | 51.29 | 2440 | 37600 | 27480 | 14C/AMS 14C | 19 |
| 234 | Gr du Spy | Middle Bed | 4.67 | 50.48 | 510 | 31180 | 29140 | 14C/AMS 14C | 19 |
| 246 | Vogelherd Cave | V | 10.2 | 48.56 | 1100 | 40040 | 34760 | 14C/AMS 14C | 19 |
| 246 | Vogelherd Cave | V | 10.2 | 48.56 | 1340 | 38160 | 31680 | 14C/AMS 14C | 19 |
| 247 | Vogelherd Cave | IV | 10.2 | 48.56 | 750 | 37230 | 34550 | 14C/AMS 14C | 19 |
| 249 | Bockstein-Torle | VII | 10.15 | 48.55 | 376 | 31330 | 30290 | 14C/AMS 14C | 19 |
| 249 | Bockstein-Torle | VII | 10.15 | 48.55 | 790 | 39650 | 35250 | 14C/AMS 14C | 19 |
| 252 | Krems-Hundssteig | N/A | 15.59 | 48.41 | 2000 | 43990 | 36310 | 14C/AMS 14C | 19 |
| 255 | Pavlov I | N/A | 16.69 | 48.87 | 350 | 30660 | 29220 | 14C/AMS 14C | 19 |
| 255 | Pavlov I | N/A | 16.69 | 48.87 | 250 | 31420 | 30940 | 14C/AMS 14C | 19 |
| 270 | Labeko Koba | V | -2.5 | 43.07 | 820 | 37290 | 34170 | 14C/AMS 14C | 19 |
| 176 | Abri Pataud | Layer 3 | 1 | 44.93 | 600 | 30730 | 28050 | 14C/AMS 14C | 19 |
| 176 | Abri Pataud | Layer 3 | 1 | 44.93 | 280 | 22690 | 21530 | 14C/AMS 14C | 19 |
| 184 | Grotte du Renne, Arcy-sur-Cure | XI | 3.77 | 47.59 | 1400 | 42640 | 35720 | 14C/AMS 14C | 19 |
| 184 | Grotte du Renne, Arcy-sur-Cure | XI | 3.77 | 47.59 | 570 | 37810 | 35450 | 14C/AMS 14C | 19 |
| 185 | Roc de Combe [Nadaillac] | 5 | 1.33 | 44.07 | 700 | 35480 | 31000 | 14C/AMS 14C | 19 |
| 185 | Roc de Combe [Nadaillac] | 5 | 1.33 | 44.07 | 1000 | 39940 | 35020 | 14C/AMS 14C | 19 |
| 229 | Trou Magrite | 3 | 4.97 | 50.21 | 3400 | 40130 | 25570 | 14C/AMS 14C | 19 |
| 229 | Trou Magrite | 3 | 4.97 | 50.21 | 1690 | 48080 | 42200 | 14C/AMS 14C | 19 |
| 139 | Les Canalettes [12 - Nant] | 2 | 3.25 | 43.99 | 6000 | 79500 | 67500 | TL | 19 |
| 3 | Labeko Koba | IV | -2.5 | 43.07 | 2400 | 50440 | 41920 | 14C/AMS 14C | 20 |
| 388 | Andrei Polar Station, SW Taymyr | N//A | 112.37 | 72.27 | 40 | 11270 | 11150 | 14C/AMS 14C | 21 |
| 398 | Baikura-Neru Bay, Taymyr Lake | N//A | 100 | 74.15 | 180 | 13410 | 12690 | 14C/AMS 14C | 21 |
| 472 | Condover Shropshire | N/A | -2.76 | 52.64 | 180 | 15130 | 13730 | 14C/AMS 14C | 21 |
| 472 | Condover Shropshire | N/A | -2.76 | 52.64 | 120 | 14970 | 13890 | 14C/AMS 14C | 21 |
| 479 | Cueto de la Mina | N//A | -4.84 | 43.42 | 500 | 24720 | 22280 | 14C/AMS 14C | 21 |
| 484 | Dobranichevka | N//A | 34.98 | 48.59 | 200 | 16180 | 14260 | 14C/AMS 14C | 21 |
| 504 | Eliseevichi | N//A | 34.55 | 53.29 | 360 | 16950 | 13590 | 14C/AMS 14C | 21 |
| 504 | Eliseevichi | N//A | 34.55 | 53.29 | 140 | 16790 | 14950 | 14C/AMS 14C | 21 |
| 507 | Engelgard Lake | N//A | 110.3 | 75.1 | 100 | 12150 | 11230 | 14C/AMS 14C | 21 |
| 514 | Etiolles | N//A | 2.47 | 48.63 | 220 | 14700 | 13380 | 14C/AMS 14C | 21 |
| 534 | Gönnersdorf | N//A | 7.25 | 50.3 | 100 | 17930 | 17370 | 14C/AMS 14C | 21 |
| 534 | Gönnersdorf | N//A | 7.25 | 50.3 | 90 | 18170 | 17530 | 14C/AMS 14C | 21 |
| 537 | Gough's Cave | N/A | -2.78 | 51.28 | 130 | 14670 | 13750 | 14C/AMS 14C | 21 |
| 580 | Herttoniemi | N//A | 25.03 | 60.2 | 200 | 19110 | 18470 | 14C/AMS 14C | 21 |
| 599 | Islands of the October Revolution | N//A | 98 | 79.44 | 610 | 15180 | 12060 | 14C/AMS 14C | 21 |
| 623 | Kesslerloch Cave | N//A | 8.79 | 47.63 | 110 | 17450 | 17210 | 14C/AMS 14C | 21 |
| 691 | La Colombière Rockshelter | N//A | 5.37 | 46.08 | 300 | 17630 | 15150 | 14C/AMS 14C | 21 |
| 724 | Lockarp | N//A | 13.03 | 55.51 | 120 | 17020 | 15220 | 14C/AMS 14C | 21 |
| 724 | Lockarp | N//A | 13.03 | 55.51 | 110 | 17330 | 15450 | 14C/AMS 14C | 21 |
| 724 | Lockarp | N//A | 13.03 | 55.51 | 95 | 17280 | 16200 | 14C/AMS 14C | 21 |
| 730 | Lower Yuribei River | N//A | 69.07 | 69.05 | 120 | 12790 | 11950 | 14C/AMS 14C | 21 |
| 758 | Mezherich | N//A | 35.77 | 48.57 | 200 | 16870 | 14670 | 14C/AMS 14C | 21 |
| 787 | Mutnaya Seyakha River | N/A | 78.22 | 70.26 | 50 | 12560 | 11960 | 14C/AMS 14C | 21 |
| 801 | Nizhnaya Taymyra River, lower stream | N/A | 96.46 | 75.88 | 60 | 11320 | 10720 | 14C/AMS 14C | 21 |
| 801 | Nizhnaya Taymyra River, lower stream | N/A | 96.46 | 75.88 | 50 | 11380 | 11180 | 14C/AMS 14C | 21 |
| 801 | Nizhnaya Taymyra River, lower stream | N/A | 96.46 | 75.88 | 100 | 12630 | 11670 | 14C/AMS 14C | 21 |
| 822 | Oelknitz | N/A | 11.61 | 50.84 | 100 | 17530 | 17250 | 14C/AMS 14C | 21 |
| 855 | Pin Hole Cave | N/A | -1.1957 | 53.264 | 160 | 15310 | 14030 | 14C/AMS 14C | 21 |
| 869 | Praz Rodet | N//A | 6.93 | 46.95 | 210 | 15140 | 13660 | 14C/AMS 14C | 21 |
| 875 | Puurmani | N/A | 26.28 | 58.57 | 100 | 12150 | 11230 | 14C/AMS 14C | 21 |
| 895 | Sabbettayaha River | N//A | 71.7 | 71.36 | 130 | 12760 | 11880 | 14C/AMS 14C | 21 |
| 906 | Settepolesini di Bondeno | N//A | 11.44 | 44.8 | 500 | 42750 | 39550 | 14C/AMS 14C | 21 |
| 908 | Severnaya River | N//A | 112 | 75.5 | 120 | 14770 | 13850 | 14C/AMS 14C | 21 |
| 908 | Severnaya River | N//A | 112 | 75.5 | 120 | 15180 | 14180 | 14C/AMS 14C | 21 |
| 960 | Taymyr Lake | N//A | 100 | 74.15 | 80 | 14460 | 13740 | 14C/AMS 14C | 21 |
| 969 | Timonovka | N/A | 34.32 | 53.21 | 300 | 15320 | 13400 | 14C/AMS 14C | 21 |
| 1009 | Volchya Griva (1) | N//A | 82.64 | 55 | 120 | 13250 | 12730 | 14C/AMS 14C | 21 |
| 1051 | Yuribei (Yuribei carcase) | N/A | 75.41 | 70.72 | 70 | 11830 | 11190 | 14C/AMS 14C | 21 |
| 1057 | Zhidikovo Peatbog | N//A | 37.87 | 59.13 | 100 | 11510 | 10990 | 14C/AMS 14C | 21 |
| 717 | Lijoki | N//A | 25.88 | 65.07 | 950 | 39850 | 35050 | 14C/AMS 14C | 22 |
| 726 | Lohtaja | N//A | 23.48 | 64.03 | 385 | 30450 | 28330 | 14C/AMS 14C | 22 |
| 577 | Haapajärvi | N//A | 25.29 | 63.64 | 670 | 35700 | 31220 | 14C/AMS 14C | 22 |
| 798 | Nilsiä, Syväri | N/A | 28.11 | 63.22 | 315 | 28060 | 25860 | 14C/AMS 14C | 22 |
| 579 | Helsinki, Töölö | N//A | 24.93 | 60.16 | 350 | 28930 | 27370 | 14C/AMS 14C | 22 |
| 393 | Arrie | N/A | 13.08 | 55.52 | 390 | 23870 | 22110 | 14C/AMS 14C | 23 |
| 393 | Arrie | N/A | 13.08 | 55.52 | 3050 | 43100 | 29460 | 14C/AMS 14C | 23 |
| 420 | Bioley-Orjulaz | N/A | 6.36 | 46.68 | 2700 | 44210 | 34450 | 14C/AMS 14C | 23 |
| 447 | Cae Gwyn Cave, Wales | N//A | -3.38 | 53.25 | 1400 | 24880 | 18360 | 14C/AMS 14C | 23 |
| 471 | Condover Shropshire | N//A | -2.76 | 52.64 | 130 | 14580 | 13620 | 14C/AMS 14C | 23 |
| 471 | Condover Shropshire | N//A | -2.76 | 52.64 | 160 | 15310 | 14030 | 14C/AMS 14C | 23 |
| 471 | Condover Shropshire | N//A | -2.76 | 52.64 | 96 | 15120 | 14400 | 14C/AMS 14C | 23 |
| 471 | Condover Shropshire | N//A | -2.76 | 52.64 | 160 | 15810 | 14450 | 14C/AMS 14C | 23 |
| 471 | Condover Shropshire | N//A | -2.76 | 52.64 | 390 | 17410 | 14090 | 14C/AMS 14C | 23 |
| 471 | Condover Shropshire | N//A | -2.76 | 52.64 | 700 | 24710 | 21550 | 14C/AMS 14C | 23 |
| 472 | Condover Shropshire | N/A | -2.76 | 52.64 | 160 | 15230 | 13910 | 14C/AMS 14C | 23 |
| 474 | Conningbrook Pit | N//A | 0.8853 | 51.169 | 1300 | 42240 | 35480 | 14C/AMS 14C | 23 |
| 474 | Conningbrook Pit | N//A | 0.8853 | 51.169 | 1600 | 43360 | 36840 | 14C/AMS 14C | 23 |
| 474 | Conningbrook Pit | N//A | 0.8853 | 51.169 | 1900 | 45240 | 38480 | 14C/AMS 14C | 23 |
| 474 | Conningbrook Pit | N//A | 0.8853 | 51.169 | 2400 | 46540 | 39540 | 14C/AMS 14C | 23 |
| 490 | Dosebacka | N//A | 12.23 | 57.7 | 1550 | 43700 | 37980 | 14C/AMS 14C | 23 |
| 517 | Farnham, Surrey | N//A | -0.8 | 51.2 | 1900 | 45030 | 38150 | 14C/AMS 14C | 23 |
| 519 | Favang | N//A | 10.37 | 61.43 | 250 | 24440 | 23360 | 14C/AMS 14C | 23 |
| 519 | Favang | N//A | 10.37 | 61.43 | 980 | 29300 | 24340 | 14C/AMS 14C | 23 |
| 578 | Hadsund | N//A | 10.11 | 56.71 | 440 | 30930 | 29130 | 14C/AMS 14C | 23 |
| 616 | Kelsterbach | N//A | 8.52 | 50.06 | 410 | 19800 | 18320 | 14C/AMS 14C | 23 |
| 616 | Kelsterbach | N//A | 8.52 | 50.06 | 2500 | 41390 | 29510 | 14C/AMS 14C | 23 |
| 643 | King Arthur's Cave | N/A | -2.6568 | 51.836 | 1500 | 43130 | 36610 | 14C/AMS 14C | 23 |
| 643 | King Arthur's Cave | N/A | -2.6568 | 51.836 | 2300 | 46260 | 39700 | 14C/AMS 14C | 23 |
| 648 | Kiskelund | N//A | 9.43 | 54.87 | 1010 | 39870 | 34830 | 14C/AMS 14C | 23 |
| 689 | Kvam | N//A | 9.1 | 61.97 | 900 | 31030 | 27430 | 14C/AMS 14C | 23 |
| 692 | La Croze-sur Suran | 1 | 5.09 | 46.87 | 260 | 18200 | 17160 | 14C/AMS 14C | 23 |
| 693 | La Croze-sur Suran | 2 | 5.09 | 46.87 | 350 | 18890 | 17370 | 14C/AMS 14C | 23 |
| 694 | La Mère Clochette Grotte | N//A | 5.56 | 47.12 | 700 | 31540 | 29380 | 14C/AMS 14C | 23 |
| 723 | Little Rissington | N//A | -1.71 | 51.87 | 500 | 41970 | 38770 | 14C/AMS 14C | 23 |
| 734 | Lundebjerg 1 | N//A | 12.4 | 55.72 | 970 | 40210 | 35570 | 14C/AMS 14C | 23 |
| 785 | Munke Bjergby | N/A | 11.54 | 55.5 | 420 | 30220 | 28060 | 14C/AMS 14C | 23 |
| 788 | Myrup Banke | N/A | 11.83 | 55.18 | 430 | 27490 | 24450 | 14C/AMS 14C | 23 |
| 830 | Orsjo | N/A | 13.55 | 55.46 | 900 | 28710 | 24110 | 14C/AMS 14C | 23 |
| 830 | Orsjo | N/A | 13.55 | 55.46 | 2000 | 44390 | 37070 | 14C/AMS 14C | 23 |
| 834 | Ostrupgard | N/A | 12.35 | 56.02 | 560 | 31310 | 29270 | 14C/AMS 14C | 23 |
| 835 | Oxbow, Leeds | N/A | -1.56 | 53.8 | 1720 | 45320 | 41120 | 14C/AMS 14C | 23 |
| 858 | Pin Hole Cave, England | N/A | -1.19 | 53.26 | 550 | 32030 | 30430 | 14C/AMS 14C | 23 |
| 892 | Ronninge 1 | N//A | 10.62 | 55.35 | 760 | 35100 | 30740 | 14C/AMS 14C | 23 |
| 893 | Rosmos 1 | N//A | 10.88 | 56.3 | 760 | 18190 | 13630 | 14C/AMS 14C | 23 |
| 903 | Saxkobing | N//A | 11.65 | 54.78 | 950 | 36800 | 31800 | 14C/AMS 14C | 23 |
| 931 | Spadzista St. [B] | N//A | 19.924 | 50.053 | 350 | 25110 | 23270 | 14C/AMS 14C | 23 |
| 931 | Spadzista St. [B] | N//A | 19.924 | 50.053 | 1050 | 27460 | 22100 | 14C/AMS 14C | 23 |
| 931 | Spadzista St. [B] | N//A | 19.924 | 50.053 | 900 | 27750 | 22910 | 14C/AMS 14C | 23 |
| 931 | Spadzista St. [B] | N//A | 19.924 | 50.053 | 170 | 28330 | 27410 | 14C/AMS 14C | 23 |
| 974 | Toten. Opland | N/A | 10.87 | 64.33 | 1200 | 25650 | 19730 | 14C/AMS 14C | 23 |
| 974 | Toten. Opland | N/A | 10.87 | 64.33 | 2300 | 37760 | 28520 | 14C/AMS 14C | 23 |
| 974 | Toten. Opland | N/A | 10.87 | 64.33 | 3100 | 43770 | 30410 | 14C/AMS 14C | 23 |
| 228 | Shandon Cave | N/A | -7.63 | 52.01 | 320 | 31290 | 30330 | 14C/AMS 14C | 24 |
| 228 | Shandon Cave | N/A | -7.63 | 52.01 | 550 | 36990 | 34990 | 14C/AMS 14C | 24 |
