# Supplementary material for: Climate Change, Humans, and the Extinction of the Woolly Mammoth
Source: PLoS Biol. 2008 Apr 1;6(4):e79. doi: 10.1371/journal.pbio.0060079 (PMC2276529; doi:10.1371/journal.pbio.0060079)
Supplement: Supplementary file 2 [file pbio.0060079.sd002.doc]

Protocol S1 for

**Climate change, humans and the extinction of the woolly mammoth**

Nogués-Bravo, D.*, Rodríguez, J., Hortal, J., Batra, P. & Araújo, M.B.

*To whom correspondence should be addressed. E-mail: davidnogues@mncn.csic.es

**Protocol S1**

**1. BIOCLIM and Maxent models of woolly mammoth’s niche**.

In addition to the Mahalanobis distance (MD) model presented in the core section of the paper, two additional predictive modelling techniques (BIOCLIM and Maxent) were used to evaluate alternative climate-envelope estimates of the changes in the reduction of the area of suitable conditions for the woolly mammoth. In both cases, we used the same data as in the MD analyses to generate the models (i.e., the same presence data and R_ann_, T_cm_ and T_wm_ as niche descriptors; we choose our variables following two requisites. First, the variables should describe the basic axis of the climatic niche. Temperature and precipitation are proxies of direct resources such as energy and water availability and they have been extensively used in the field of niche modeling to model different groups of species and taxa. Secondly, outputs of General Circulation Models, GCMs, have different levels of uncertainty. Basic temperature variables such as the ones used in our manuscript are the ones that could be modelled by GCMs with the most certainty. Annual precipitation has more uncertainty than temperature, but less than other more complex variables related to water availability). The results were projected to the five palaeoclimatic scenarios used (126, 42, 30, 21 and 6 kyr BP), converting the suitability scores into four quartiles to allow comparison with the main results. Also, we compare the predicted distribution of the most suitable conditions of the three techniques with the treeline reconstruction for northern Eurasia [S1] in order to assess the quality of these predictions. This analysis was important because there is evidence that different modelling techniques may provide different projections [S2], and we needed to make sure that our choice of climate-envelope technique was robust when compared to alternative modelling techniques.

BIOCLIM

BIOCLIM is a simple methodology, which describes the bioclimatic niche of the species as all the range within the bounds marked by the extreme values (maximum and minimum) with species presences in a number of environmental variables (i.e., environmental envelope [S3-S4]). Due to its simplicity, it makes very few assumptions about the relationship of the species with the environment, so it has been used to project the distribution of species to both the past and the future [S5-S7].

We used a slight modification of BIOCLIM to generate estimates not only for the presence of adequate conditions for the species, but also for the adequacy of these conditions in the form of quartiles of similar interpretation as the ones developed using MD. Quartile values for the presence of the species in the three predictors were mapped, and each geographic cell was assigned to the less-fit quartile (i.e., if a given cell was Q2 for R_ann_, Q1 for T_cm_ and Q4 for T_wm_, then it is assigned to Q4 in the final map).

Maxent

We also modelled the response of the woolly mammoths to climatic gradients with Maxent [S8]. Briefly, this method aims to find the probability distribution of maximum entropy – the closest to uniform – in the environmental conditions occupied by the species, subject to constraints imposed by the information available on the observed distribution of the species and the environmental conditions across the study area. We split the suitability scores (ranging from 0 [maximum suitability] to 100 [minimum suitability]) obtained for each time projection into quartiles.

Model Evaluation

The term ‘model evaluation’ describes the assessment process required to justify the acceptance of a model – usually from different competing models – as being accurate enough to serve for an intended purpose [S9]. Discrepancies between model results have been assessed and discussed elsewhere [S10-S11], and the consensus is that model choices should be made considering the purpose of a particular study and the quality of their predictions. The most appropriate approach for evaluating climatic niche projections is independent validation, which requires that the data used to train the models is evaluated against statistically independent data [S12-S13]. Our evaluation of the niche conservatism hypothesis using Mahalanobis Distance (see main text) provides a (successful) independent validation of the MD predictions, Also, we performed a bootstrap with *N*=1000 resampling runs to assess the stability of MD projections to the initial conditions. This was important because it allowed testing the robustness of models to the quality of the fossil record.

In addition to these model evaluation exercises, we use the past distribution of tundra – an independent data set (treeline reconstruction for northern Eurasia [S1] that delimits forests and open tundra ) – to evaluate the results from the three different techniques used to model the climatic niche of the woolly mammoth: Mahalanobis Distance, BIOCLIM and Maxent. Firstly, we used the Holocene (6kyr BP) treeline reconstruction for northern Eurasia [S1] that delimits forests and open tundra. We geo-referenced this information and converted it into a binomial variable (grid cells covered by trees were classified as 0; grid cells covered by open tundra were classified as 1). Secondly, assuming that the woolly mammoth – typically described as a tundra species – was living in open tundra, we consider that coincidence between highly suitable conditions for the woolly mammoth, as modelled by climate envelope techniques, and the occurrence of open tundra provides a fair evaluation of the quality of our models. We used each of the three first quartiles, Q1, Q2 and Q3, as the thresholds above which the woolly mammoth populations are more likely to be considered present. We restricted analyses to the same area studied by MacDonald and colleagues [S1] to map the reconstruction of the treeline. To evaluate how well predicted ranges correspond to those of the independent dataset, we assess commission, Ec and omission errors, Eo [S14]:

Ec = (c - o)/m

Eo = (m – o)/m

where m is (the size of) the area of tundras at 6kyr BP period, c is the area where the climatic envelope models predict the presence of woolly mammoths and o is the area where m and c overlap.

Error of commission is a measure of model overprediction, i.e., the model predicts that suitable conditions are present in places where the species is not expected to be present (in this case, that the species is present in forest areas). Error of omission is a measure of underprediction, i.e., the model fails to predict suitable conditions in places where the species is expected to be present (in this case, in open tundra areas). In our analysis, a perfect model would have Ec=0 and Eo=0.

Models of the woolly mammoth’s niche created with Mahalanobis Distance predict better than BIOCLIM and Maxent the location of the habitat of the woolly mammoth, open tundra (6 kyr BP period), in inland Siberia. Errors of omission are similar for the three techniques, but errors of commission, (overprediction) are six times larger for BIOCLIM and Maxent than for MD (see histograms below). These results indicate that Mahalanobis distance is the best technique to predict the climatic niche of woolly mammoths in our study.

Area reduction

BIOCLIM predicts a reduction of 96.7% of the area of the most suitable conditions, Q1, in Eurasia between 42 kyr BP BP and 6 kyr BP BP periods, which is quite consistent with the area reduction projected by Mahalanobis Distance for Q1 (89%). Maxent predictions are more optimistic, predicting a reduction of 36.5% of the area with the most suitable conditions represented by Q1.

**Predicted distribution for woolly mammoth’s niche at 6kyr BP BP period by Maxent (upper map), BIOCLIM (centre map) and Mahalanobis distance (lower map)**. Green line represents treeline reconstruction (*Betula* spp., birch forest) for northern Siberia for the same period.

**2. Sensitivity tests to identify which climatic variable most contributed to the Holocene range collapse of the woolly mammoth**

We measure the importance of each climatic variable to clarify which climatic variable contributed most to explain the distribution of the woolly mammoth at the 6 kyr BP period. We followed the following protocol: a model is calibrated using each variable in isolation using the same approach described in the methods section of the main text. We projected the resulting niche to the 6 kyr BP period and then we obtained the Mahalanobis Distance scores projected for each of the occurrences of woolly mammoth dated within the 6 kyr BP period. Then we averaged these values. We assume that lower average MD scores correspond to a better prediction of the occurrences of woolly mammoth dated within the 6 kyr BP period. Results show that minimum temperature and precipitation, 2.54 and 2.55 averaged scores of MD respectively, predict better the occurrences of woolly mammoth in the 6 kyr BP period than maximum temperature, which gave average MD scores of 4.32.

**3. Hunting intensity values, HI_t,_ through time**.

Irrespectively of the Cull Rate, CR, included in the model, a clear pattern of change in the HI_t_ (Hunting Intensity) values through time arises (Suplementary Table 3). The number of woolly mammoths that is necessary to be killed per person per year to drive them to extinction is rather similar for the 45-39 kyr BP and 33-27 kyr BP time intervals (these intervals were called 42 kyr BP and 30 kyr BP periods across the main text), it decreases for the 24-18 kyr BP time interval (21 kyr BP period) and is very low at 6 kyr BP. These results lend to the conclusion that a vigorous woolly mammoth population with a high density (4 ind/km^2^) could be driven to extinction 6 kyr BP with a hunting intensity of 0.37 ind/per/yr. Put in other words, a woolly mammoth killed every three years by each human being. The value of HI*_t_* is as low as 0.0049 ind/per/yr for a less vigorous population with a very low density (roughly a woolly mammoth killed by each person every 200 yr). These results also lead to the conclusion that a suboptimal woolly mammoth population with a low density (4 ind/km^2^) could be driven to extinction at 6 kyr BP with a very low hunting intensity of 0.0049 ind/per/yr.

**Supplementary Online Material: References**

S1. MacDonald GM et al. (2000) Holocene treeline history and climate change across northern Eurasia. Quaternary Res 53: 302-311

**S2. Araújo MB**, New M (2007) Ensemble forecasting of species distributions. Trends Ecol Evol 22: 42-47

S3. Busby JR (1986) A biogeographical analysis of *Notophagus cunninghamii* (Hook.) in south-eastern Australia. Aust J Ecol 11: 1–7.

S4. Busby JR (1991) BIOCLIM - A bioclimate analysis and prediction system. In: Margules CR, Austin MP, editors. Nature Conservation: Cost effective biological surveys and data analysis. Melbourne: CSIRO. pp 64-68

S5. Eeley HAC, Lawes MJ, Piper SE The influence of climate change on the distribution of indigenous forest in KwaZulu-Natal, South Africa. J Biogeog 26: 595-617 (1999)

S6. Dimitriadis S, Cranston PS (2001) An Australian Holocene climate reconstruction using Chironomidae from a tropical volcanic maar lake. Palaeogeog Palaeoclim Palaeoecol 176: 109-131.

S7. Beaumont LJ, Hughes L, Poulsen M (2005) Predicting species distributions: use of climatic parameters in BIOCLIM and its impact on predictions of species' current and future distributions. Ecol Model 186: 251-270.

S8. Phillips SJ, Anderson RP, Schapire RE (2006) Maximum entropy modeling of species geographic distributions. Ecol Model 190: 231-259.

S9. Araújo MB, Guisan A (2006) Five (or so) challenges for species distribution modelling. J Biogeogr 33: 1677-1688

S10. Araújo MB, Whittaker RJ, Ladle RJ, Erhard M (2005) Reducing uncertainty in extinction risk from climate change. [Global Ecol Biogeogr](http://www.mncn.csic.es/pdf_web/maraujo/Araujo_et_al_2005GEBuncertainty.pdf) 14: 529-538

S11. Pearson RG, Thuiller W, Araújo MB, Martinez E, Brotons L, McClean C, Miles L, Segurado P, Dawson T, Lees D (2006) Model-based uncertainty in species’ range prediction. J Biogeogr 33: 1704-1711

S12. Araújo MB, Pearson RG, Thuiller W, Erhard M (2005) Validation of species-climate impact models under climate change. Glob Change Biol 11: 1504-1513

S13. Araújo MB, Rahbek C (2006) How does climate change affect biodiversity? [Science](http://www.mncn.csic.es/pdf_web/maraujo/Araujo_Rahbek2006Science.pdf) 313: 1396-1397

S14. Hijmans RJ, Graham CH (2006) The ability of climate envelope models to predict the effect of climate change on species distributions. Global Change Biol 12: 2272-2281.

S15. Bocquet-Appel J-P, Demars P-Y, Noiret L and Dobrowsky D (2005) Estimates of Upper Paleolithic meta-population size in Europe from archaeological data. J Archaeolog Sci 32: 1656-1668.

S16. Koch PL, Barnosky AD (2006) Late Quaternary Extinctions: State of the Debate. Annu Rev Ecol Evol Syst 37: 215-250.

S17. Martinez-Meyer E, Townsend Peterson A, Hargrove WW (2004) Ecological niches as stable distributional constraints on mammal species, with implications for Pleistocene extinctions and climate change projections for biodiversity. Glob Ecol Biogeog 13: 305-314.
