# Supplementary material for: Climate Change, Humans, and the Extinction of the Woolly Mammoth
Source: PLoS Biol. 2008 Apr 1;6(4):e79. doi: 10.1371/journal.pbio.0060079 (PMC2276529; doi:10.1371/journal.pbio.0060079)
Supplement: Supplementary file 9 [file pbio.0060079.st001.doc]

Table 1a:

| Variable | *N* | *df* | *Chi-Square* | *P*(F) |
| --- | --- | --- | --- | --- |
| R_ann_ | 52 | 2 | 1.24 | 0.536 |
| T_cm_ | 52 | 2 | 1.36 | 0.504 |
| Tw_m_ | 52 | 2 | 3.36 | 0.186 |

Table 1b:

| Variable | *N* | *df* | *Chi-Square* | *P*(F) |
| --- | --- | --- | --- | --- |
| R_ann_ | 141 | 2 | 1.24 | 0.349 |
| T_cm_ | 141 | 2 | 1.36 | 0.859 |
| Tw_m_ | 141 | 2 | 3.36 | 0.219 |

Table 1c:

| Variable | *N* | *df* | *Chi-Square* | *P*(F) |
| --- | --- | --- | --- | --- |
| R_ann_ | 270 | 2 | 11.365 | 0.03 |
| T_cm_ | 270 | 2 | 8.023 | 0.018 |
| Tw_m_ | 270 | 2 | 13.852 | 0.001 |

R_ann_ - annual precipitation (mm/year)

T_cm_ – mean temperature of the coldest month

T_wm_ - mean temperature of the warmest month
