# Supplementary material for: Climate Change, Humans, and the Extinction of the Woolly Mammoth
Source: PLoS Biol. 2008 Apr 1;6(4):e79. doi: 10.1371/journal.pbio.0060079 (PMC2276529; doi:10.1371/journal.pbio.0060079)
Supplement: Supplementary file 10 [file pbio.0060079.st002.doc]

| **Cull Rate** | **Dm** | **NK** | | | | **Hi_t_** | | | |
| --- | --- | --- | --- | --- | --- | --- | --- | --- | --- |
|  |  | **42 kyr BP** | **30 kyr BP** | **21 kyr BP** | **6 kyr BP** | **42 kyr BP** | **30 kyr BP** | **21 kyr BP** | **6 kyr BP** |
|  | 0.1 | 130140 | 128723 | 140073 | 38853 | 0.041 | 0.040 | 0.044 | 0.012 |
|  | 0.4 | 520560 | 514890 | 560293 | 155412 | 0.164 | 0.150 | 0.107 | 0.038 |
|  | 0.6 | 780840 | 772335 | 840440 | 233118 | 0.245 | 0.225 | 0.160 | 0.057 |
|  | 0.8 | 1041120 | 1029780 | 1120586 | 310824 | 0.327 | 0.300 | 0.214 | 0.076 |
| 2.7 | 1.2 | 1561680 | 1544670 | 1680880 | 466236 | 0.491 | 0.450 | 0.321 | 0.114 |
|  | 1.4 | 1821960 | 1802115 | 1961026 | 543942 | 0.573 | 0.525 | 0.374 | 0.133 |
|  | 1.6 | 2082240 | 2059560 | 2241173 | 621648 | 0.655 | 0.600 | 0.428 | 0.152 |
|  | 2.2 | 2863080 | 2831895 | 3081613 | 854766 | 0.900 | 0.825 | 0.588 | 0.208 |
|  | 3 | 3904200 | 3861675 | 4202199 | 1165590 | 1.227 | 1.125 | 0.802 | 0.284 |
|  | 4 | 5205600 | 5148900 | 5602932 | 1554120 | 1.636 | 1.500 | 1.069 | 0.379 |
|  | 0.1 | 16870 | 16686 | 18158 | 5037 | 0.005 | 0.005 | 0.006 | 0.002 |
|  | 0.4 | 67480 | 66745 | 72631 | 20146 | 0.021 | 0.019 | 0.014 | 0.005 |
|  | 0.6 | 101220 | 100118 | 108946 | 30219 | 0.032 | 0.029 | 0.021 | 0.007 |
|  | 0.8 | 134960 | 133490 | 145261 | 40292 | 0.042 | 0.039 | 0.028 | 0.010 |
| 0.35 | 1.2 | 202440 | 200235 | 217892 | 60438 | 0.064 | 0.058 | 0.042 | 0.015 |
|  | 1.4 | 236180 | 233608 | 254207 | 70511 | 0.074 | 0.068 | 0.049 | 0.017 |
|  | 1.6 | 269920 | 266980 | 290522 | 80584 | 0.085 | 0.078 | 0.055 | 0.020 |
|  | 2.2 | 371140 | 367098 | 399468 | 110803 | 0.117 | 0.107 | 0.076 | 0.027 |
|  | 3 | 506100 | 500588 | 544730 | 151095 | 0.159 | 0.146 | 0.104 | 0.037 |
|  | 4 | 674800 | 667450 | 726306 | 201460 | 0.212 | 0.194 | 0.139 | 0.049 |
