# Supplementary material for: Climate Change, Humans, and the Extinction of the Woolly Mammoth
Source: PLoS Biol. 2008 Apr 1;6(4):e79. doi: 10.1371/journal.pbio.0060079 (PMC2276529; doi:10.1371/journal.pbio.0060079)
Supplement: Supplementary file 11 [file pbio.0060079.st003.doc]

| Cultural Period |  | Population density (ind./km^2^) | | | Population size (ind.) | | |
| --- | --- | --- | --- | --- | --- | --- | --- |
|  | Cal kyr BP | Average | Minimum | Maximum | Average | Minimum | Maximum |
| Aurignacian | 40.36-31.04 | 0.168 | 0.066 | 1.077 | 4424 | 1738 | 28359 |
| Gravettian | 31.04-23.50 | 0.183 | 0.072 | 1.172 | 4776 | 1879 | 30589 |
| Glacial  Maximum | 23.50-17.18 | 0.257 | 0.101 | 1.646 | 5885 | 2313 | 37693 |
| Late Glacial | 17.18-11.36 | 0.722 | 0.285 | 1.825 | 28736 | 11343 | 72635 |
